# Role of Amorphous Phases in Mixed Conduction of Conjugated Regioblock Copolymers for Organic Electrochemical Synaptic Transistors

**DOI:** 10.1002/adma.202502133

**Published:** 2025-08-30

**Authors:** Kwang‐Hun Choi, Seongil Im, Aaron Plant, Carlos Neri Soto, Hanna Lee, Changsoon Choi, Ho Won Jang, Jeong Ho Cho, Hyunsu Ju, Youngmin Lee, Jung Ah Lim

**Affiliations:** ^1^ Electronic and Hybrid Materials Research Center Korea Institute of Science and Technology Seoul 02792 Republic of Korea; ^2^ Center for Quantum Technology Korea Institute of Science and Technology Seoul 02792 Republic of Korea; ^3^ Department of Materials Science and Engineering Research Institute of Advanced Materials Seoul National University Seoul 08826 Republic of Korea; ^4^ Department of Chemical Engineering New Mexico Tech New Mexico 87801 USA; ^5^ Division of Nano and Information Technology KIST School University of Science and Technology of Korea (UST) Seoul 02792 Republic of Korea; ^6^ Department of Chemical and Biomolecular Engineering Yonsei University Seoul 03722 Republic of Korea; ^7^ Advanced Institute of Convergence Technology Seoul National University Suwon 16229 Republic of Korea; ^8^ Department of Materials Science and Engineering YU‐KIST Institute Yonsei University Seoul 03722 Republic of Korea

**Keywords:** artificial synaptic transistor, conjugated polymer, crystallinity dependent redox properties, neuromorphic devices, organic electrochemical electronics, organic mixed ionic‐electronic conductors, *regioblock* copolymer

## Abstract

Growing interest in organic electrochemical synaptic transistors (OECT‐STrs) based on conjugated polymer mixed ionic‐electronic conductors (CP‐MIECs) has intensified, leading to the need to establish clear design rules and fundamentally understand the distinct roles of crystalline and amorphous domains in the electrochemical doping behavior of CP‐MIEC films. Here, OECT‐STrs based on regioregular‐block‐regiorandom (regioblock) conjugated copolymers with precisely controlled crystallinity are demonstrated. The crystallinity of a poly(3‐hexylthiophene) regioblock copolymer is systematically tuned by varying the fraction of regiorandom blocks without altering the geometry or orientation of the crystalline phase. It is shown that incorporating an amorphous phase into the active channel of OECT‐STrs significantly enhances the neuromorphic learning efficiency by improving modulation uniformity. This improvement results from sequential polaron formation in the amorphous regions and bipolaron formation in the crystalline domains during potentiation. Additionally, crystalline phases provide better state retention at low potentiation states, while amorphous phases contribute to improved long‐term retention at high potentiation states by enhancing charge carrier localization through stronger Coulombic interactions. Neural network simulations based on actual device conductance demonstrate that OECT‐STrs with a high amorphous fraction consume only 18% of the power required by devices with a highly crystalline film, owing to the need for fewer repetitive learning cycles.

## Graphical abstract

Critical roles of amorphous and crystalline phases in polymer‐based mixed ionic‐electronic conductors for organic electrochemical synaptic transistors using conjugated regioregular‐block‐regiorandom copolymers are demonstrated. Amorphous phases contribute to gradual polaron formation and strong ion trapping by bipolarons, leading to improved synaptic state modulation. The synaptic transistors with appropriate amorphous content exhibited low power consumption in the neuromorphic learning process.

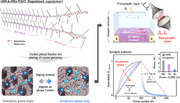

## 1 Introduction

Given the rapid growth in demand for artificial intelligence and machine learning technologies, neuromorphic computing has emerged as a cutting‐edge approach for energy‐efficient parallel information processing.^[^ ^1^, ^2^, ^3^ ^]^ This computing paradigm mimics biological computation systems based on neural networks. Artificial synapse devices, which can simultaneously control electrical response and memory states using instantaneous potential stimuli, are essential for neuromorphic computing.^[^ ^4^, ^5^, ^6^, ^7^ ^]^ Among the various types of artificial synaptic devices, electrolyte‐gated synaptic transistors (EGSTs) have emerged as promising candidates for artificial synapses. The ability of EGSTs to emulate both short‐ and long‐term plasticity through coupled ionic and electronic dynamics closely resembles the behavior of biological neural networks.^[^ ^8^, ^9^, ^10^, ^11^ ^]^ In particular, EGSTs based on organic electrochemical transistors (OECT‐STrs), which consist of conjugated polymer‐based mixed ionic‐electronic conductor films (CP‐MIECs) and ion‐containing gate dielectrics (e.g., electrolytes or ion‐gels), have shown promising behavior in terms of low‐power manipulation of synaptic plasticity through an electrochemical doping mechanism.^[^ ^12^, ^13^, ^14^, ^15^, ^16^, ^17^ ^]^ Recent advancements in OECT‐STrs have shown the compatibility of these synaptic transistors with biological neural systems, extending their potential applications to implantable artificial neural systems.^[^ ^18^, ^19^ ^]^ However, despite these significant advances, the fundamental mechanisms governing the electrochemical behaviors of CP‐MIEC channels under synaptic stimuli and their correlation with synaptic functions remain incompletely understood.

The synaptic responses of OECT‐ STrs, including spiking‐relaxation, potentiation/depression, paired‐pulse facilitation, and retention behaviors, are governed by the electrochemical doping kinetics within the CP‐MIECs channel when the pulse bias is applied through the ionic gate dielectric. Studies on the synaptic properties of OECT‐STrs have mainly been fundamental investigations of the underlying OECT devices.^[^ ^20^, ^21^, ^22^, ^23^ ^]^ Previous studies have shown that the structural parameters of CP‐MIEC films, such as crystallinity, molecular orientation, and compatibility with the electrolyte, are intimately involved in the generation of doped charge carriers and their transconductance states during device operation.^[^ ^24^, ^25^, ^26^, ^27^ ^]^ Moreover, other studies showed that, in addition to influencing the infiltration of ionic species from the electrolyte into the CP‐MIEC layer, these structural parameters also exert complex effects on electrochemical reactions and charge carrier mobility.^[^ ^28^, ^29^, ^30^ ^]^

Among the structural parameters of CP‐MIEC films, film crystallinity, in particular the fraction of amorphous domains within the film, has been shown to have conflicting effects on OECT device performance. The semi‐crystalline nature of CP‐MIECs presents this critical area of investigation, focusing on the crystallinity‐dependent mixed ionic‐electronic conduction properties of the CP‐MIEC films. In general, crystalline domains with ordered chain stacking facilitate electronic charge carrier transport, leading to a high conductivity of the CP‐MIEC channel.^[^ ^31^ ^]^ Conversely, highly crystalline regions may impede the infiltration and diffusion of ionic species into CP‐MIEC channels.^[^ ^24^ ^]^ This can restrict the electrochemical reactions necessary for regulating the electrical states of CP‐MIEC channels, disrupting the overall OECT performance. These conflicting effects highlight the need to establish design rules for optimizing the balance between amorphous and crystalline phases in CP‐MIEC channels to achieve high‐performance OECT‐STrs.

Various crystalline engineering methods have been applied to the synthesis of CP‐MIEC films with a view to achieving high transconductance in OECTs.^[^ ^32^, ^33^, ^34^, ^35^, ^36^ ^]^ Flagg et al. demonstrated that thermal annealing of poly(3‐{[2‐(2‐methoxyethoxy)ethoxy]methyl}thiophene‐2,5‐diyl) (P3MEEMT) film increased the crystalline ordering, resulting in water uptake that disrupted the intergrain conductivity and hence lowered the transconductance.^[^ ^33^ ^]^ By contrast, in a study on the influence of crystallinity on OECT device performance, Wang et al. found that thermal annealing of PTBT‐p films enhanced both charge transport and memory retention.^[^ ^37^ ^]^ As another approach, Wu et al. examined the effect of regulating the molecular weight of poly(benzimidazobenzophenanthroline) (BBL) in n‐type CP‐MIEC channels. They observed improved crystallinity in a certain molecular weight range, which led to strong *π–π* interactions and hence high transconductance.^[^ ^35^ ^]^ In a more recent in situ spectro‐electrochemistry study of poly(3‐hexylthiophene) (P3HT) films whose crystallinity was controlled by blending its regio‐isomer, Jackson et al. suggested that the infiltrated ions and generated polarons tend to be localized to the locally ordered domains.^[^ ^38^ ^]^ Although this spectroscopic study provided fundamental insights into charge carrier formation, the direct correlation between crystallinity and OECT characteristics was not explored. Thus, these previous studies yielded varying conclusions regarding the impact of crystallinity on OECT performance, mainly because the crystallinity engineering methods employed simultaneously alter multiple structural parameters, for example, crystal size and orientation, making it challenging to isolate the specific role of crystallinity.

Previous studies have also sought to improve the synaptic responses of OECT‐STrs, in particular the long‐term plasticity and retention, by engineering the crystallinity of CP‐MIEC channels. While thermal annealing has been employed to enhance the crystallinity of CP‐MIEC channels for synaptic transistors, using P3HT and PTBT‐p polymer semiconductors, this approach has the unwanted effect of altering the size and orientation of crystal domains.^[^ ^20^, ^37^ ^]^ In a different approach, improved molecular packing of PDPP3T‐based polymer through control of alkyl side‐chain length was shown to improve long‐term plasticity; however, the observed improvements were primarily attributed to changes in lamellar stacking distance and the size and orientation of crystals rather than quantitative variations in crystallinity.^[^ ^39^ ^]^ The approaches used to date (e.g., thermal annealing, side‐chain engineering, and blending of isomeric molecules) have successfully demonstrated that CP‐MIEC channel crystallinity can be modified and impacts device performance. However, in addition to changing the overall crystallinity, these methods often introduce complex structural variations, making it challenging to establish clear links between crystallinity and synaptic characteristics or to distinguish the individual roles of crystalline and amorphous regions in the electrochemical doping kinetics of OECT‐STr devices. To develop clearer design principles for CP‐MIEC channels and gain deeper insights into the distinct contributions of crystalline and amorphous domains to electrochemical doping behavior, a strategy that enables precise control of crystallinity while preserving geometric properties such as crystal size and orientation is needed.

In the present study, we elucidate the critical roles of amorphous and crystalline phases in CP‐MIEC channels for organic electrochemical synaptic transistors using conjugated block copolymers. Poly(3‐hexylthiophene) (P3HT) regioblock copolymers (RR‐b‐RRa P3HT), consisting of a crystalline regioregular (RR) segment and an amorphous regiorandom (RRa) segment, provide an ideal platform for controlling the fraction of crystalline or amorphous phases while maintaining the characteristics of the crystalline domains.^[^ ^40^, ^41^ ^]^ The chemical homogeneity of the polythiophene chain backbone enables a focused investigation into the impact of crystallinity on the device's electrical properties. Previously, conjugated block copolymers have been used in organic thin‐film transistors and photovoltaic solar cells for optimizing the optical, electrical, and mechanical robustness of the semiconductor active layer.^[^ ^41^, ^42^ ^]^ However, to our knowledge, the present study is the first to apply such copolymers in OECTs. In this work, we systematically investigate the impact of crystallinity on synaptic characteristics by using P3HT regioblock copolymers as CP‐MIEC channels in OECT‐STrs. Through a comprehensive analysis of morphological properties and electrochemical characteristics, we aim to establish fundamental design principles for optimizing the crystalline‐amorphous balance in CP‐MIEC channels to achieve synaptic devices with enhanced signal uniformity and energy efficiency.

## 2 Results and Discussion

**Figure 1:**
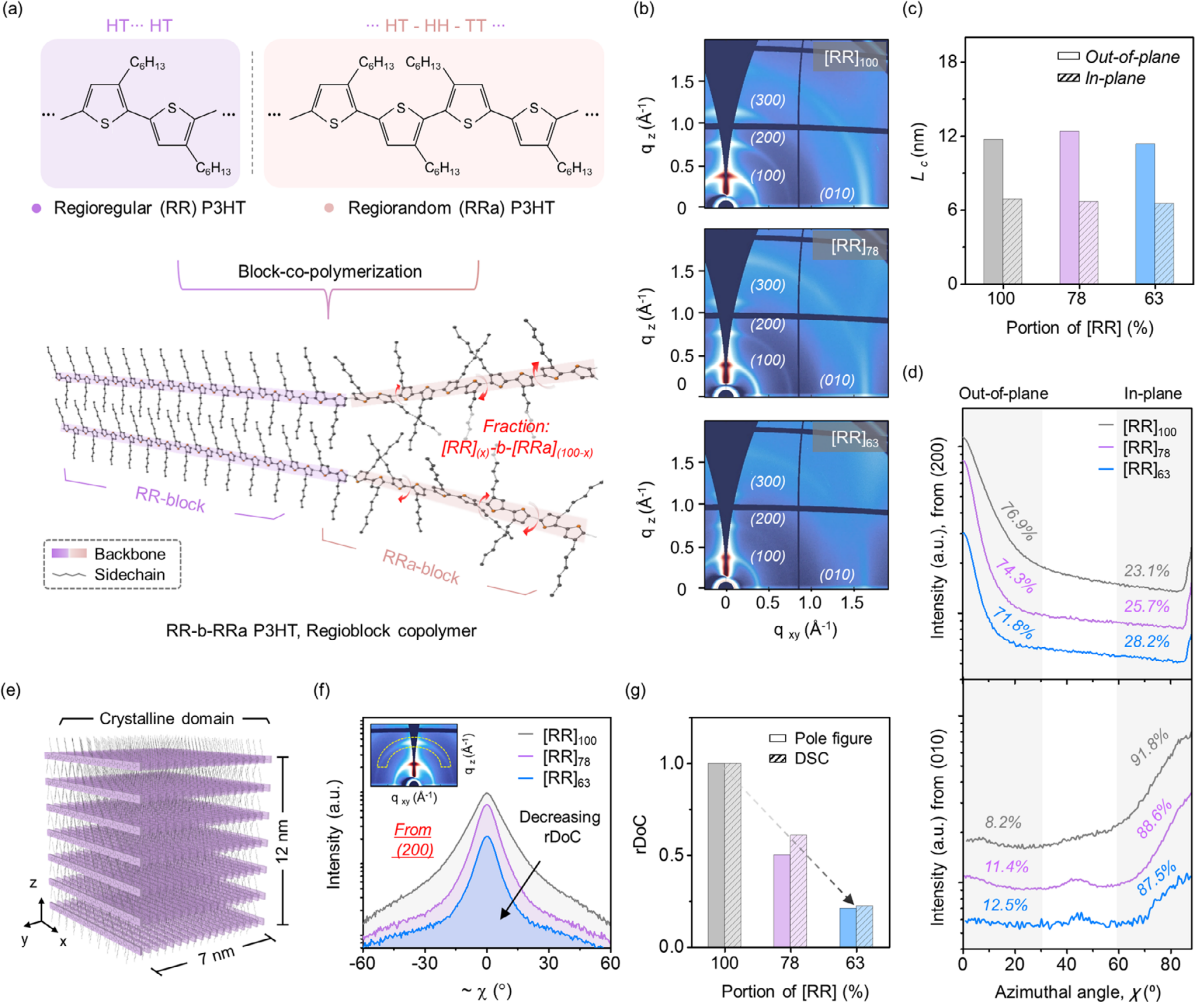
Characterization of the microstructure of RR‐b‐RRa P3HT thin films. a) Molecular structures of the RR‐ and RRa‐blocks in P3HT block copolymers. b) 2D‐GIWAXS images of RR‐b‐RRa P3HT films with varying compositions. c) Crystalline domain size (L_c_) determined from 1D line‐cut profiles of the GIWAXS images. d) Azimuthal profiles at the (200) and (010) scattering peaks of the GIWAXS images. Some profiles have been vertically shifted for display purposes. e) Illustration of a crystalline domain. f) Pole figures from the (200) scattering peaks in the angular range of ±60°. g) Relative degree of crystallinity (rDoC) of RR‐b‐RRa P3HT films, with values expressed relative to the [RR]_100_ value.

Regioregular*‐block‐*regiorandom poly(3‐hexylthiophene) block copolymers, referred to as RR‐b‐RRa P3HTs, were synthesized; the molecular structure is shown in Figure **1**a. The regioregularity of a conjugated polymer is defined as the fraction of the regioregular (RR) configuration, i.e., *Head*‐to‐*Tail*, along the chain backbone. The regiorandom (RRa) configuration can be prepared by incorporating *Head*‐to‐*Head* dithiophene units with thiophene monomers during polymerization. The composition of the RR‐b‐RRa P3HTs was systematically regulated by varying the length of the RRa block. The detailed synthetic procedure is described in the Experimental section (Supporting Information), and the characterization of the RR‐b‐RRa P3HTs (M_n_ 14–17 kg mol^−1^, Đ < 1.6) is described in Figure S1, Table S1, and Note S1 (Supporting Information). The compositions of the RR‐b‐RRa P3HTs were analyzed by ^1^H nuclear magnetic resonance (NMR), Figure S2 (Supporting Information), by comparing the α‐CH_2_ peak from the *RR* block at 2.8 ppm and that from the *RRa* block at 2.6 ppm. Two RR‐b‐RRa P3HT samples were selected for further study, [RR]:[RRa] 63:37 and 78:22, along with RR P3HT homopolymer as a control. For simplicity, hereinafter, these polymers are denoted as [RR]_63_, [RR]_78_, and [RR]_100_.

The microstructure of the RR‐b‐RRa P3HT thin films was investigated using grazing incidence wide‐angle X‐ray scattering (GIWAXS). As shown in Figure 1b, all of the thin films exhibit (*h*00) scattering peaks corresponding to lamellar stacking of polymer backbones along the q_z_ direction (i.e., out‐of‐plane), as well as a (010) scattering peak corresponding to *π–π* interaction along the q_xy_ direction (i.e., in‐plane). The lamellar spacing and *π–π* stacking distance were 1.66 and 0.384 nm, respectively (Figure S3c, Supporting Information). These spacings are the same for the [RR]_63_, [RR]_78_, and [RR]_100_ films, indicating that they are unaffected by the introduction of the RRa block. Next, the average size of crystalline domains (L_c_) was extracted from 1D line‐cut profiles of the GIWAXS results by determining the full width at half maximum of scattering peaks and applying Scherrer's equation (Figure S3a and b).^[^ ^43^, ^44^ ^]^ Notably, the L_c_ values of all of the thin films fall within a small deviation of ±7% (Figure 1c), with the average L_c_ for the three film types being 12.08 ± 0.90 nm from the (100) peak and 6.70 ± 0.15 nm from the (010) peak. Lastly, the orientation of the crystalline domains (e.g., either edge‐on or face‐on) was extracted from azimuthal profiles at the (200) and (010) scattering peaks in the GIWAXS maps (Figure 1d). The out‐of‐plane scattering at the (200) peak and the in‐plane scattering at the (010) peak represent the edge‐on orientation. The fraction of the edge‐on orientation was similar for the three films, with the average values across the three films for the (200) and (010) peaks being 74.3 ± 2.6% and 89.7 ± 2.1%, respectively. Similar to the L_c_ results, introducing the RRa block had a negligible effect on the crystalline domain orientation. Therefore, the analysis of the microstructure of the RR‐b‐RRa P3HT thin films indicates that incorporation of the RRa block does not noticeably impact the size or orientation of the crystalline domains, as depicted in Figure 1e.

To quantitatively analyze the crystalline phase fraction in the films, we compared the relative degree of crystallinity (rDoC) of the RR‐b‐RRa P3HTs. The rDoC was determined by comparing the integrated area of the (200) peak in pole figures over the angular range of ± 60°,^[^ ^41^, ^44^ ^]^ as shown in Figure 1f. The rDoC values of the [RR]_78_ and [RR]_63_ films were 0.50 and 0.21, respectively. These values were in good agreement with the rDoC values determined using differential scanning calorimetry (DSC), shown in Figure 1g. A detailed explanation of the DSC analysis is provided in Figure S4 and Note S2 (Supporting Information). Therefore, the results of the microstructure and rDoC analyses of the RR‐b‐RRa P3HT films show that varying the RRa fraction in RR‐b‐RRa P3HTs enables systematic control of a proportion of the amorphous phase in P3HT thin films while preserving the characteristics of the crystalline phase.

**Figure 2:**
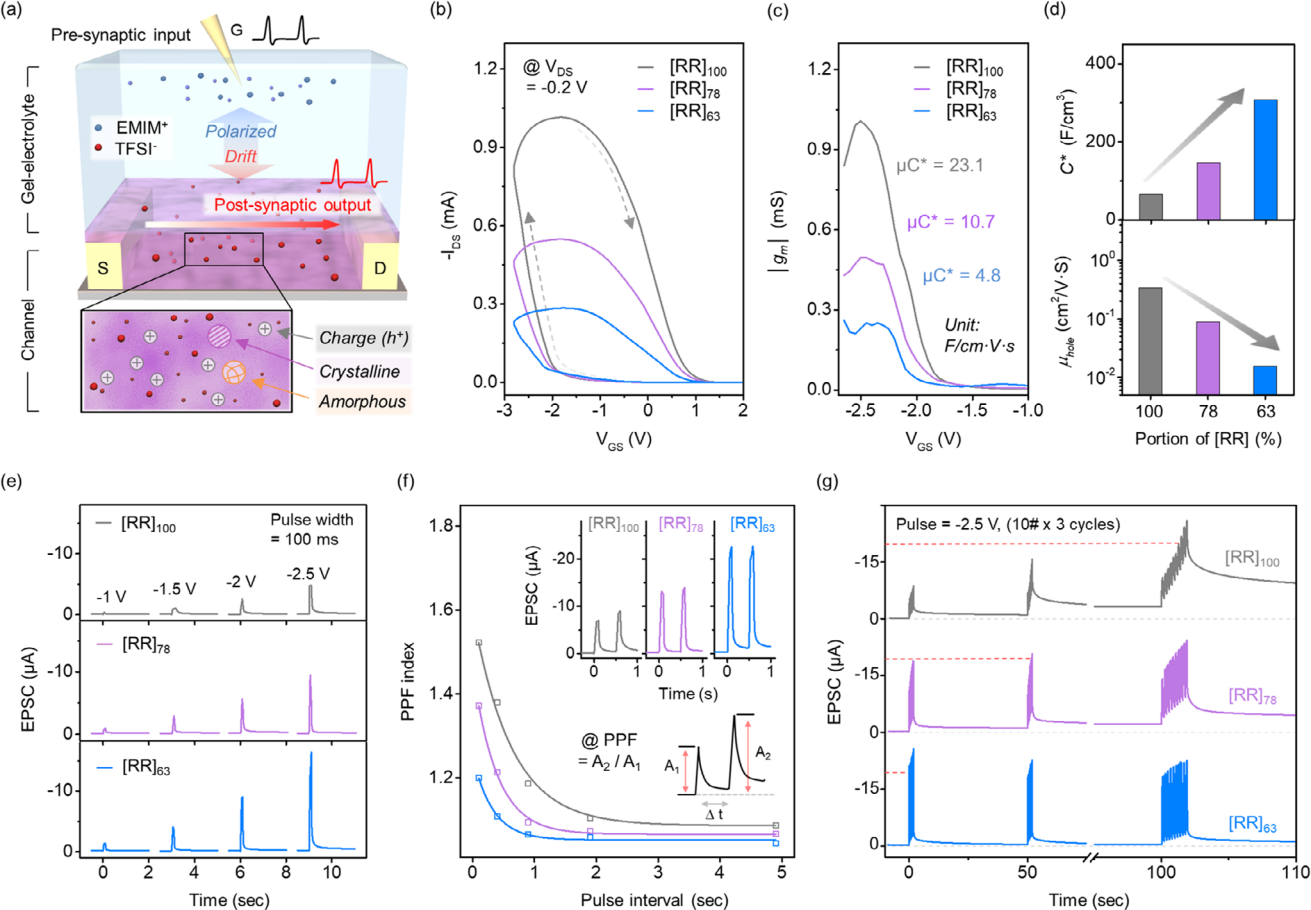
Performance of RR‐b‐RRa P3HT synaptic transistors (STrs). a) Schematic illustration of RR‐b‐RRa P3HT STrs. b) Transfer curves and c) transconductance (g_m_) for RR‐b‐RRa P3HT STrs. d) Volumetric capacitance (C^*^) and hole mobilities (µ _hole_) of RR‐b‐RRa P3HT channels, with values extracted by decoupling the µC^*^ of STrs. e) Spike‐stimulus strength‐dependent EPSC responses of RR‐b‐RRa P3HT STrs. f) Paired‐pulse facilitation (PPF) characteristics as a function of spike‐stimulus interval time. g) Leaky‐integration behaviors of RR‐b‐RRa P3HT STrs under repetitive spike‐train presynaptic stimuli.

Organic electrochemical synaptic transistors (STrs) based on [RR]_100_, [RR]_78_, and [RR]_63_ P3HT films were fabricated using an ion‐gel gate dielectric consisting of poly(vinylidene fluoride‐co‐hexafluoropropylene) (P(VDF‐HFP)) and 1‐ethyl‐3‐methylimidazolium bis (trifluoromethyl sulfonyl) imide [EMIM]^+^ [TFSI]^−^ ionic liquid, as shown in Figure **2**a. Under a negative gate bias (V_GS_), TFSI anions in the ion‐gel layer drift into the RR‐b‐RRa P3HT semiconductor channel, inducing electrochemical doping reactions that result in an increase in the source‐drain current (I_DS_).^[^ ^15^, ^33^ ^]^ Figure 2b shows typical transfer curves of the [RR]_100_, [RR]_78_, and [RR]_63_ STrs. All devices exhibit p‐type accumulation mode operation, with turn‐on near the gate voltage (V_GS_) = ‐2 V, resulting in a large drain current (I_DS_) during the forward gate bias sweep. This increase in I_DS_ is not due to the redox behavior of the EMIM^+^ cation and TFSI^−^ anion, which typically occurs at voltages over ±3 V (Figure S5, Supporting Information). The emergence of a large clockwise hysteresis during gate bias sweeps indicates a non‐monotonic variation in chemical potential with gate‐source voltage, which can be attributed to electrochemical doping due to ion infiltration and trapping within the RR‐b‐RRa channel.^[^ ^45^ ^]^ Figure 2c presents the transconductance (*g_m_*) of the devices estimated from forward transfer characteristics based on the following equation:^[^ ^46^ ^]^

(1) $$g_{m}=\partial I_{DS}/\partial V_{GS}=\left(Wd/L\right)\;\mu C^{*}\left(V_{TH}-V_{GS}\right)$$

where *µ* is the electronic carrier mobility, C^*^ is the volumetric capacitance of the channel, and *W*, *L*, and *d* are the channel width, channel length, and thickness of the active layer, respectively. The W, L, and d of the three STrs were set to 500 µm, 50 µm, and 50 nm, respectively. The maximum *g_m_* decreases more than 4‐fold with increasing the amorphous fraction (i.e., RRa ratio), from 1.01 mS cm^−1^ for [RR]_100_ to 0.25 mS cm^−1^ for [RR]_63_. The quantitative µC^*^ values for the three STrs, which indicate the steady‐state mixed ionic‐electronic transport properties of the channels, were calculated to be 23.1, 10.7, and 4.8 F cm^−1^ V^−1^ s^−1^ for the [RR]_100_, [RR]_78_, and [RR]_63_ films, respectively (Figure 2c). This result is comparable to previously reported µC^*^ values for OECT channels (of 10^0^–10^2 ^cm^−1^ V^−1^ s^−1^).^[^ ^26^, ^32^, ^33^, ^34^, ^35^, ^47^, ^48^, ^49^, ^50^, ^51^ ^]^

To clarify the origin of the rDoC‐dependent g_m_ of the devices, the contributions to C* were decoupled and estimated using electrochemical impedance spectroscopy (EIS), as shown in Figure S6 (Supporting Information). Under a gate bias of 2.5 V, the C^*^ of all films was significantly enhanced compared to that measured at 0 V condition, as a result of the pronounced infiltration of ions within the film. The shift in phase angle to a lower value suggests the emergence of resistive behavior stemming from the electrochemical redox behaviors of the channels.^[^ ^50^ ^]^ The extracted values of C^*^ in Figure 2d show an increase from 65.7 for the [RR]_100_ film to 146.3 and 307.8 F cm^−3^ for the [RR]_78_, and [RR]_63_ films, respectively. Correspondingly, the hole mobility (µ_h_) of the films was estimated to be 0.34, 0.09, and 0.015 cm^2^ V^−1^ s^−1^. This result indicates that, although the capacitance contribution from infiltrated ions in the P3HT channels increases as the amorphous fraction increases, the resulting degradation in charge carrier mobility has a more significant effect on the transconductance of the RR‐b‐RRa P3HT STrs. At this point, g_m_ is merely one of the parameters used to evaluate the performance of OECT devices. It is more important to understand how the rDoC‐dependent changes in both capacitive and charge‐carrier transport behaviors under transient gate pulses influence the synaptic characteristics of the devices.

Next, we examined whether the STrs can function effectively as artificial synapses. To achieve this, time domain‐temporal spike inputs were applied through the gate (presynaptic neuron), and the resulting current responses in the I_DS_ (postsynaptic neuron) were integrated to determine the various synaptic state levels.^[^ ^52^ ^]^ Figure 2e presents the excitatory postsynaptic current (EPSC), I_DS,_ responses of the RR‐b‐RRa P3HT STr when various presynaptic biases, ranging from −1 to −2.5 V, were applied at a fixed pulse duration of 100 ms. All of the STrs exhibited a typical EPSC strengthening trend as the presynaptic pulse strength was increased. Notably, the EPSC amplitude evoked by the spike stimuli increased substantially with increasing the amorphous fraction in the RR‐b‐RRa P3HT channel, despite the transconductance of the channel with a larger amorphous fraction being lower.

For RR‐b‐RRa P3HT STrs to function effectively as artificial synapses, the EPSC spike responses of RR‐b‐RRa P3HT STrs must be both integrated and modulated to various current levels. This process relies on the synaptic plasticity of the device, which is influenced by the strength of the bias stimuli and the pulse‐spike interval (∆t) between incoming presynaptic spikes.^[^ ^53^ ^]^ To evaluate the spike‐driven potentiation characteristics of the RR‐b‐RRa P3HT STrs, the paired‐pulse facilitation (PPF) index, the ratio of the peak amplitude of the second EPSC to that of the first (A_2_/A_1_), was investigated as a function of pulse interval, as shown in Figure 2f. The observed exponential increase in PPF index with decreasing ∆t is attributed to incomplete relaxation of the spike EPSC.^[^ ^54^ ^]^ Interestingly, while the amplitude of a single EPSC spike response increased with increasing RRa‐fraction in the RR‐b‐RRa P3HT channel, the degree of paired‐pulse potentiation tended to decrease. This suggests that the spike response and the integrated potentiation of EPSC under continuous synaptic spike stimuli depend on the rDoC of the channel. This rDoC dependency of EPSC spike responses leads to pronounced discrepancies in leaky integration behavior under spike‐train presynaptic stimuli, as shown in Figure 2g. The 10 presynaptic pulses of −2.5 V (100 ms width and interval) were applied to individual RR‐b‐RRa P3HT STrs across 3 cycles with a relaxation time of 48 s between cycles. The [RR]_100_ STr, with a high rDoC, exhibited a gradual increase in resting current after each cycle, rising from −1.06 to −3.15 µA. After 30 presynaptic pulses, the peak current eventually reached the threshold current level (arbitrarily set at −20 µA, indicated by the dashed red line), which was the value set for triggering signal firing to neighboring neurons in the synaptic neural network process. In contrast, the [RR]_63_ STr, with low rDoC, reached the threshold after only one pulse due to its larger EPSC amplitude. The gradual increase in the EPSC peak amplitude was smaller, and the resting current level after three presynaptic cycles remained lower (from −0.43 to −0.44 µA) compared to that of [RR]_100_. This result demonstrates that optimizing rDoC is not only a key factor in achieving precise control of EPSC amplitude but is also crucial for regulating synaptic state levels.

Additionally, we measured the EPSC responses of the RR‐b‐RRa P3HT STrs when exposed to various presynaptic pulse strengths from −1 to −2.5 V, with ∆t ranging from 0.1 to 9.9 s over a total 25‐s span (Figure S7, Supporting Information). At larger ∆t, all STrs exhibited temporary and reversible increases in EPSC, mimicking biological short‐term potentiation (STP). As ∆t decreased, the EPSC spikes were integrated, transitioning into more persistent long‐term potentiation (LTP). The detailed conditions for the STP‐to‐LTP transitions are summarized in Table S2 (Supporting Information). The results show that all three RR‐b‐RRa P3HT STrs are capable of mimicking both STP and LTP behaviors by adjusting the presynaptic bias conditions. As expected, EPSC amplitude increased with decreasing rDoC of the channel, while LTP behavior could be emulated under weaker and sparser presynaptic pulse conditions when rDoC was higher. These results highlight the need for a fundamental understanding of how ions infiltrate and dope the P3HT active channel at different amorphous fractions, and how these changes influence the EPSC behaviors of the STr device.

**Figure 3:**
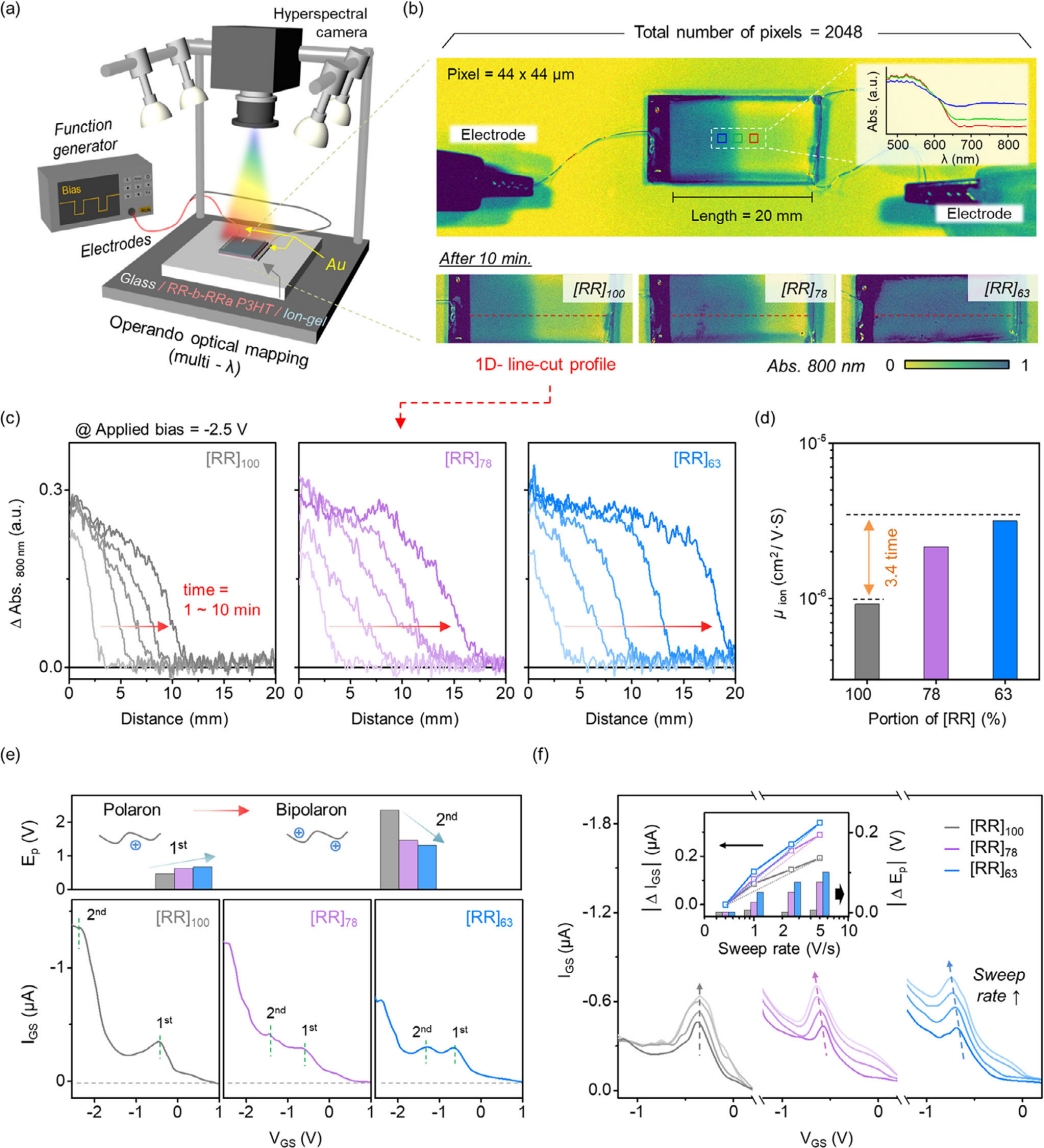
Dynamic ionic motions and electrochemical oxidation in RR‐b‐RRa P3HT films and STrs. a) Schematic of the hyperspectral imaging system used for in situ profiling of electrochemical doping behavior in RR‐b‐RRa P3HT channels. b) Method for quantifying ion drift distance based on doping time (top); the inset shows absorbance spectra for three regions (blue, green, and red squares in the mapping images at 800 nm). Mapping images of three different RR‐b‐RRa P3HT films after 10 min of doping (bottom). c) 1D‐profiles of polaron state absorbance intensity as a function of distance. d) Calculated ionic mobility in RR‐b‐RRa P3HT films. e) V_GS_ versus I_GS_ curves and oxidation peak potentials (E_p_) of RR‐b‐RRa P3HT STrs. f) Sweep rate‐dependent I_GS_ curves of RR‐b‐RRa P3HT STrs, the inset shows the calculated differences in I_GS_ and E_p_ as a function of sweep rate.

To investigate the rDoC‐dependent, dynamic ionic motion in the RR‐b‐RRa P3HT films, we used hyperspectral imaging to examine the in situ positional profiles of the doped states generated in the films (Figure **3**a). It is well established that the neutral and charged doping states of P3HT can be analyzed using the absorption spectrum.^[^ ^29^, ^30^ ^]^ The spectral components observed ≈500, 800, and 1600 nm were assigned to neutral, polaron (singly doped), and bipolaron (doubly doped) charged species, respectively.^[^ ^29^ ^]^ Thus, by focusing on absorption at 800 nm, we monitored the time evolution of polaron generation in the P3HT films through electrochemical doping with the infiltrated TFSI^−^ anions. This allowed comparison of the ionic drift speeds across different rDoC levels in the RR‐b‐RRa P3HT films. Figure 3b shows the hyperspectral images, recorded over time for the RR‐b‐RRa P3HT films, while applying −2.5 V to electrodes that were laterally positioned on 20 mm samples. The results clearly demonstrate that polaronic doping propagation is much faster in the RR‐b‐RRa P3HT films with higher amorphous fractions. This behavior can be attributed to a high fraction of crystalline phases with densely packed molecules in the high‐crystallinity channel, which restricts ion transport and hence extends the pathway that must be traversed by TFSI− anions.^[^ ^24^ ^]^ Figure 3c displays the variation in absorption intensity as a function of the propagation distance extracted from the hyperspectral analysis. From the x‐intercept distance at a fixed time, the ionic velocity (*v _ion_*) in the RR‐b‐RRa P3HT films can be calculated;^[^ ^24^ ^]^ the detailed procedure is described in Figure S8 and Note S3 (Supporting Information). The ionic mobility (*µ*_ion_) in each film was then estimated using the equation, *v*_ion_ = *µ*_ion_ ·E, where the E is the applied electric field. The results show that the *µ_ion_* of [RR]_63_ is 3.4 times that of [RR]_100_, indicating that the presence of the amorphous phase significantly enhances ion drift within the RR‐b‐RRa P3HT films under an applied bias (Figure 3d). These findings support the conclusion that the higher C^*^ observed in RR‐b‐RRa P3HT STrs with lower rDoC, as shown in Figure 2d, is due to the larger quantity of infiltrated anions in the low rDoC film, resulting from the higher *µ*_ion_.

Next, to clarify how the infiltrated ions contribute to EPSC behavior, we examined the effects of the crystalline and amorphous phases on the formation of charging states from the electrochemical reaction with the ions. Figure 3e shows the I_GS_‐V_GS_ curves obtained from the RR‐b‐RRa P3HT STrs. Previous studies have established that the gate voltage corresponding to the peak I_GS_ can be interpreted as being equivalent to the oxidation potential (E_p_) in the CV measurement.^[^ ^55^ ^]^ Highly crystalline [RR]_100_ exhibited an E_p_, _1st_ near −0.45 V, which is attributed to polaron generation in the crystalline phase.^[^ ^30^, ^38^ ^]^ In contrast, the [RR]_78_ and [RR]_63_ films showed a shift in E_p, 1st_ to higher potential, along with the appearance of distinct E_p, 2nd_ stages near −1.4 V. These E_p, 2nd_, observed at higher oxidation potential compared to E_p_, _1st_, originate from the short conjugation lengths of the chains in the amorphous phases of the semi‐crystalline P3HT films.^[^ ^31^, ^56^, ^57^, ^58^ ^]^ Therefore, these findings indicate that charge generation by oxidative doping occurs more favorably in the crystalline than in the amorphous phase. To further investigate the charge generation kinetics, we examined the changes in the E_p_, _1st_ of the RR‐b‐RRa P3HT STrs by varying the V_GS_ sweep rate from 0.5 to 5 V s^−1^. As shown in Figure 3f, all of the RR‐b‐RRa P3HT STrs exhibited a nonlinear relationship between peak current and sweep rate. This observation indicates that the current generation in RR‐b‐RRa P3HT STrs is not mainly governed by diffusion‐controlled reactions, but rather is driven by the interplay between capacitive effects and the Faradaic oxidation reaction.^[^ ^59^, ^60^, ^61^ ^]^ A key observation is that the variation in peak potential (E_p_) as a function of sweep rates differed among the RR‐b‐RRa P3HTs. Specifically, the E_p_ of [RR]_100_ remained unchanged regardless of the sweep rate, suggesting that the charge transfer kinetics in highly crystalline films are sufficiently fast to maintain electrochemical equilibrium even at higher sweep rates. In contrast, [RR]_78_ and [RR]_63_ exhibited a shift of E_p_ to higher potentials as the sweep rate increased, indicating kinetic limitations in the charge transfer process.

These results can be interpreted as follows: In the highly crystalline [RR]_100_ film, the ordered molecular packing in the crystalline domains facilitates rapid charge transfer, allowing the oxidation reaction to proceed efficiently even at higher sweep rates. This efficient charge transfer process maintains the E_p_ at a constant value. On the other hand, in the [RR]_78_ and [RR]_63_ films with higher amorphous fractions, the oxidation initially occurs in the available crystalline regions, but as the sweep rate increases, these limited crystalline domains become insufficient to accommodate the faster charging process. Consequently, the oxidation extends into the amorphous regions, where higher potentials are required for charge generation due to the disordered structure, resulting in the observed shift of E_p_ to higher potential.

Considering the low field‐effect mobilities and large degree of ions infiltration in the [RR]_63_ films with a high amorphous fraction, the high amplitude and rapid decay of the EPSC response observed in the [RR]_63_ STr can be reasonably attributed to a dominant capacitive switching effect. In contrast, although ion infiltration into the highly crystalline [RR]_100_ film is lower than that observed for films with a high RRa ratio, the efficient generation of polarons in the crystalline phase through electrochemical reactions and their subsequent transport lead to strong paired‐pulse integration and higher resting currents in the EPSC response.

**Figure 4:**
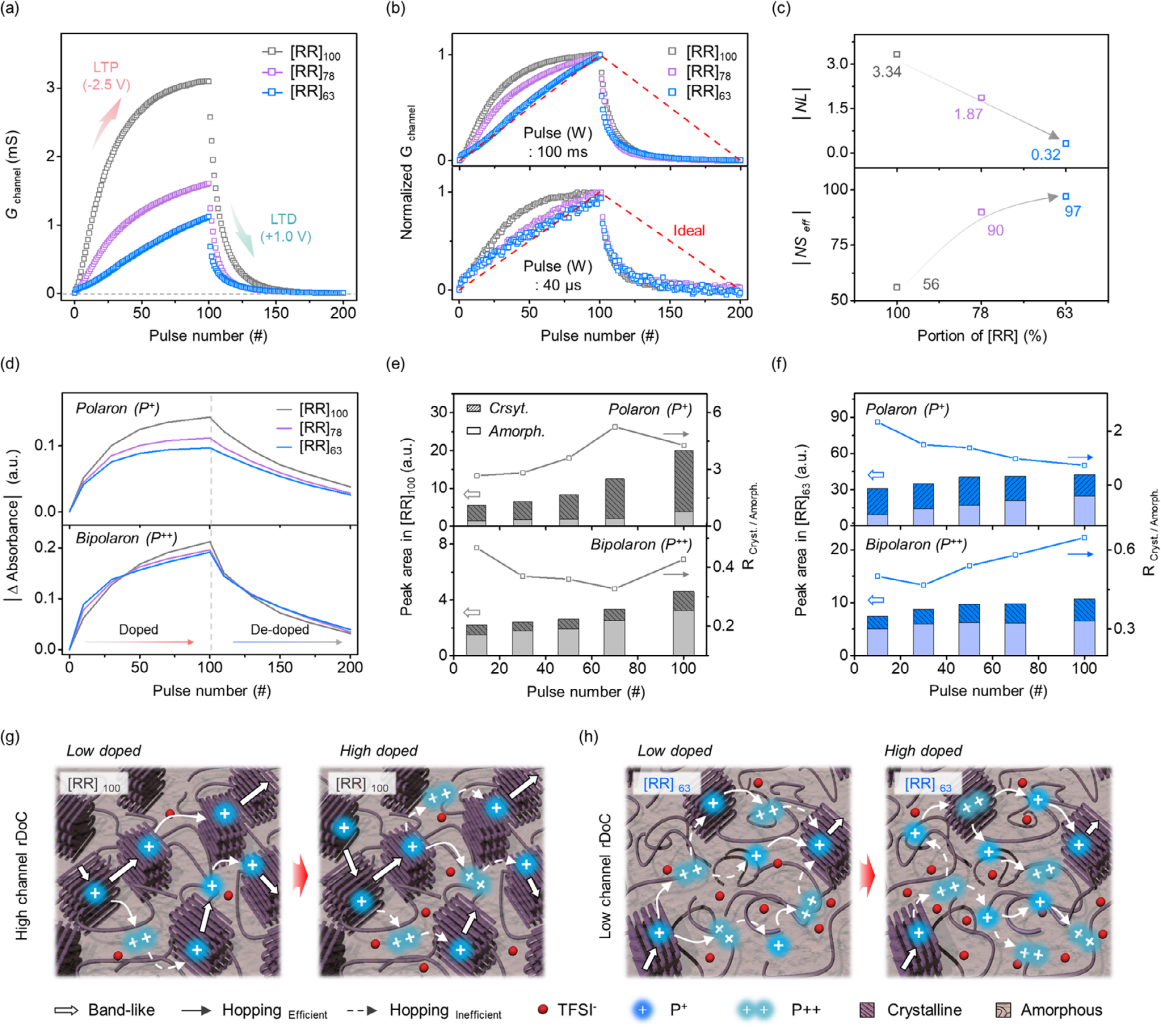
Correlation of synaptic weight update behaviors of STrs with electrochemical doping kinetics in RR‐b‐RRa P3HT channels. a) Long‐term potentiation/depression (LTP/LTD) curves of RR‐b‐RRa P3HT STrs, with 100 potentiation and depression pulses. b) Normalized LTP/LTD curves with 100 ms (top) and 40 µs (bottom) potentiation/depression pulse condition, respectively. c) Nonlinearity (NL) and effective number of state (NS_eff_) of RR‐b‐RRa P3HT STrs, as a function of [RR] fraction. d) Development and diminishment of polaron (P^+^) and bipolaron (P^++^) states, where ∆Absorbance was extracted from UV—Vis–NIR spectra. e,f) Comparison of the integrated peak area of (P^+^) and (P^++^) states for [RR]_100_ and [RR]_63_ as a function of potentiation pulse number, with values extracted from Gaussian‐deconvoluted Raman spectra. The line plots show the crystalline/amorphous charge state ratio. g,h) Schematic illustration of rDoC‐dependent doping kinetics and conductance variations in RR‐b‐RRa P3HT STr channels.

In principle, the neural network‐based learning process is based on the repetitive correction of synaptic weights according to incoming stimuli.^[^ ^1^, ^2^, ^3^ ^]^ In synaptic devices, the synaptic weights are represented by the channel conductance (G_channel_), and G_channel_ is repeatedly increased or decreased for synaptic weight correction. Figure **4**a shows the gradual LTP and long‐term depression (LTD) behaviors of the RR‐b‐RRa P3HT STrs as a function of pulse number. All of the STrs exhibited a dynamic range (G_max_/G_min_) exceeding 10^2^ under 100 presynaptic gate pulses, as shown in Figure S9 (Supporting Information). To further investigate the operational characteristics of the RR‐b‐RRa P3HT STrs, we conducted a systematic study of LTP/LTD behavior under programming pulse frequencies ranging from 5 Hz to 12.5 kHz, as shown in Figure S10 (Supporting Information). Interestingly, the RR‐b‐RRa P3HT STrs exhibited distinct LTP/LTD behavior even at 12.5 kHz (programming pulse width of 40 µs), indicating their ability to operate under high‐speed conditions. Furthermore, all of the RR‐b‐RRa P3HT STr devices exhibited a dynamic range of 10–10^2^ across this frequency range, regardless of the amorphous fraction in the channel (Figure S10f, Supporting Information). Given that the minimum required dynamic range for a successful pattern recognition task is 10, our RR‐b‐RRa P3HT STrs should be applicable to various neuromorphic computing units, utilizing their own dynamic ranges.

Notably, the progressive G_channel_ potentiation differed depending on the rDoC of the RR‐b‐RRa P3HT channel. For the [RR]_100_ STr, a relatively rapid increase in G_channel_ was observed, followed by saturation after 40 potentiation pulses. For the [RR]_78_ and [RR]_63_ STrs, by contrast, G_channel_ increased gradually without saturation, reaching lower maximum levels as rDoC decreased. The normalized LTP/LTD curves shown in Figure 4b clearly exhibit enhanced linearity in progressive potentiation as the RRa ratio increases in the RR‐b‐RRa P3HT channel, and this behavior is maintained not only at pulse widths of 100 ms but also during high‐speed operation with short pulses of 40 µs. For precise and energy‐efficient updates to G_channel_, linear modulation with a large number of separable conductance states is important.^[^ ^3^, ^13^ ^]^ Figure 4c shows the nonlinearity (NL) values and effective number of states (NS_eff_) extracted from the normalized LTP/LTD curves. The results confirm that as the amorphous fraction in the RR‐b‐RRa P3HT channel increases, NL approaches the ideal value of zero, and NS_eff_ improves. This trend is independent of the specific conductance range used for evaluation, as shown in Figures S11 and S12 (Supporting Information). Details of the performance evaluation procedures are provided in Note S4 (Supporting Information). These findings indicate that incorporating an amorphous phase into the active channel of STrs enhances the neuromorphic learning‐efficiency by ensuring a greater number of practical conductance states with improved modulation uniformity.

To understand the origin of the enhanced NL in RR‐b‐RRa P3HT STrs with a higher amorphous fraction in the channel, the electrochemical doping kinetics during the LTP and LTD processes were analyzed using in situ electro‐spectroscopic analysis. The UV‐Vis‐NIR spectra of the [RR]_100_, [RR]_78_, and [RR]_63_ films (Figure S13, Supporting Information) confirm that, as the potentiation progresses, the neutral peaks decrease, whereas the two peaks characteristic of polaronic and bipolaronic states at ≈800 and 1600 nm, respectively,^[^ ^29^ ^]^ increased significantly. Figure 4d shows a plot of the polaron and bipolaron peak intensities as a function of pulse number. The results show that, under the same potentiation conditions, polarons form preferentially in the RR‐b‐RRa P3HT channels with higher rDoC. This behavior can be attributed to the lower oxidation potential of the crystalline phase compared to the amorphous phase, as mentioned above. Interestingly, although bipolarons formed more rapidly in films with lower rDoC during the early stages of the LTP process, a greater number of bipolarons were generated in the high rDoC channel, [RR]_100_, after 30 pulses. This indicates that in the crystalline phase, polarons are preferentially formed in the initial stage, but then, with continued potentiation, bipolarons begin to form. In the low rDoC channel, the greater production of bipolarons, which is attributed to an increased likelihood of polarons coming into close proximity and interacting with each other, can be explained by the large amount of TFSI^−^ dopant that has infiltrated into the amorphous phase. This higher concentration of trapped TFSI^−^ anions in the RR‐b‐RRa P3HT channel with a high RRa fraction during the same LTP process was further supported by the results of XPS analysis (Figure S14 and S15, and Note S5, Supporting Information).

To gain deeper insight into the contributions of the crystalline and amorphous phases to the formation of doped charge states and the resulting channel conductance during the potentiation process, in situ electrochemical Raman analysis was performed. It has been reported that the intense Raman band at 1455 cm^−1^, corresponding to the C_α_‐C_β_ vibrational mode of the P3HT backbone, shifts to a lower wavenumber upon the electrochemical doping‐induced formation of polaronic states. Following the procedure suggested by Nightingale et al. and Cavassin et al., the C_α_‐C_β_ stretching peak can be deconvoluted to distinguish the contributions of the crystalline and amorphous phases in the different doped states.^[^ ^56^, ^57^ ^]^ Detailed descriptions of the sorting process for signature peaks can be found in Note S6 (Supporting Information). Figures S16 and S17 (Supporting Information) show the deconvoluted Raman spectra of RR‐b‐RRa P3HT films subjected to a sequential potentiation pulse. The relative ratio (R _Cryst./Amorph._)of the integrated areas of the polaron and bipolaron peaks originating from crystalline and amorphous regions was plotted as a function of the number of potentiation pulses, as shown in Figure 4e,f. In the case of the [RR]_100_, R _Cryst./Amorph._ value of polarons is >1.0, indicating that polarons were dominantly generated in the crystalline phase (Figure 4e). As the number of potentiation pulses increased, polaron formation in the crystalline phase increased dramatically. These polarons facilitated efficient charge transport through a combination of band‐like transport and hopping mechanisms, resulting in a significant increase in channel conductance.^[^ ^32^, ^62^ ^]^ Meanwhile, bipolarons preferentially formed in the amorphous phase, with the bipolaron R _Cryst./Amorph._ value being < 1.0. With additional potentiation up to 70 potentiation pulses, bipolaron formation in the amorphous phase gradually increased. As the number of potentiation pulses increased beyond 70 pulses, an increase in R _Cryst./Amorph._ value was observed, indicating enhanced bipolaron formation in the crystalline phase. Notably, excessive formation of bipolarons in the amorphous phase can limit charge transport between crystalline phases, due to unfavorable mixed‐valence conduction and hole‐hole repulsion.^[^ ^56^, ^63^ ^]^ Therefore, as illustrated in Figure 4g, this analysis suggests that while the polarons dominantly generated in the crystalline phase enable efficient charge transport in the channel during the early stages, progressive potentiation leads to increased bipolaron formation in the amorphous phases. Therefore, the rapid increase in localized charge carriers in the amorphous regions leads to inefficient charge transport, limiting the continuous increase in channel conductance and resulting in saturation behavior.

Interestingly, the [RR]_63_ film with a high amorphous phase fraction exhibited different doping behavior during potentiation. Because the free‐volume‐rich microstructure of the [RR]_63_ film allows a large amount of TFSI^−^ anion infiltration even at an early stage of potentiation, most of the crystalline phases in these films were oxidized to form polarons, as shown in Figure 4f. However, with further potentiation, no significant enhancement of polarons in the crystalline phase was observed, while the amorphous phases were consistently oxidized to form polarons. This increase in polarons in the amorphous phase contributes to the enhanced conductivity of the film by improving the hopping transport pathway.^[^ ^64^, ^65^ ^]^ Furthermore, it is notable that as potentiation progresses, no distinct increase in bipolarons was observed in the amorphous phase, whereas the crystalline phase continued to become doubly charged. Unlike the bipolarons formed in the amorphous phase, those in the crystalline phase are known to efficiently contribute to charge transport due to the enhanced delocalization in well‐ordered π‐conjugated systems, ultimately resulting in increased conductivity of the channel.^[^ ^29^, ^56^ ^]^ Consequently, as illustrated in Figure 4h, these results demonstrate that in [RR]_63_ films rich in amorphous phases, the gradual generation of polarons in the amorphous phase and the spread of bipolarons in the crystalline phase during potentiation progressively facilitate consistent growth of the charge transport pathway in the channel. Therefore, the G_channel_ of [RR]_63_ STrs exhibits improved NL along with LTP behavior without saturation.

**Figure 5:**
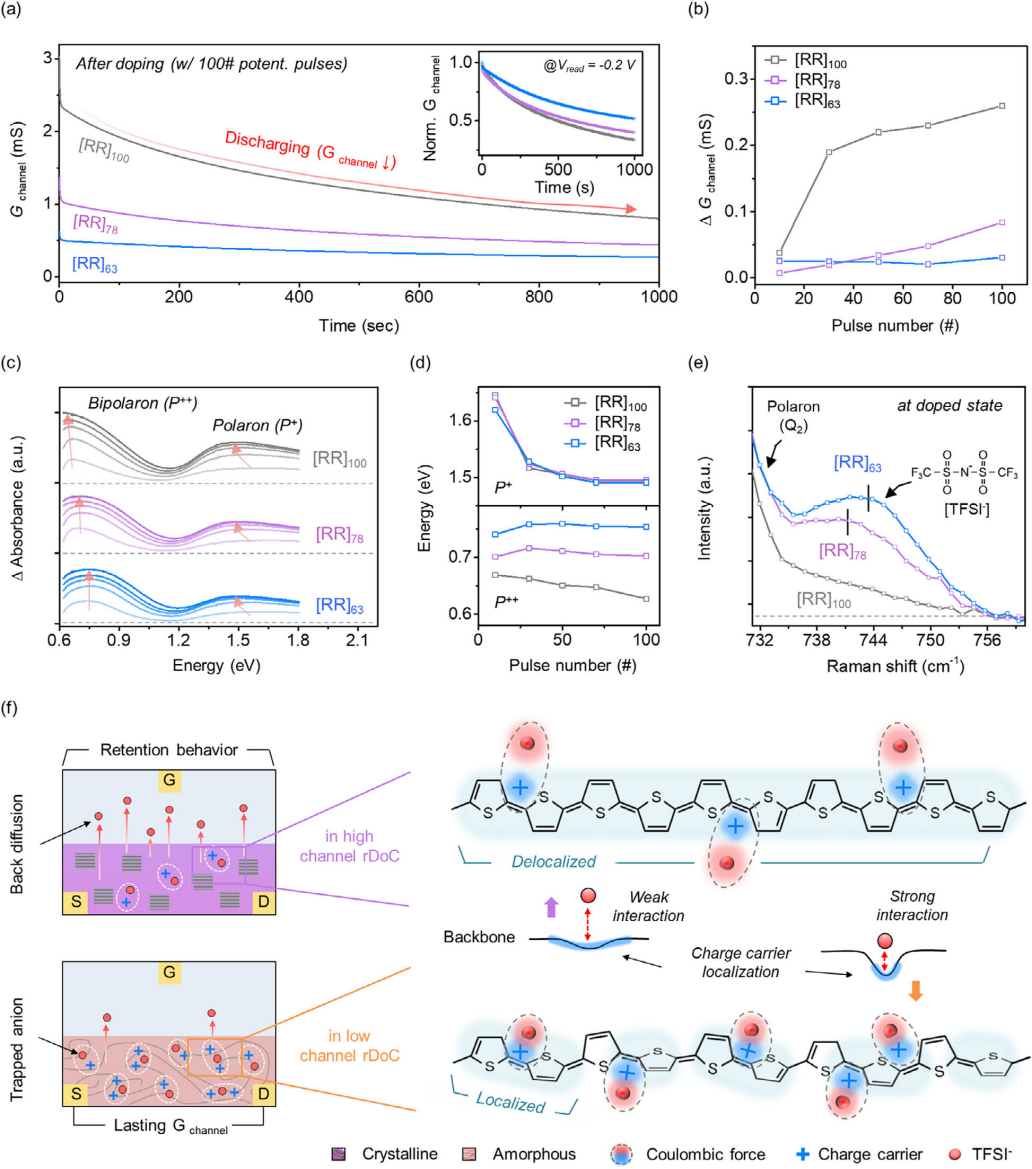
Long‐term state retention in RR‐b‐RRa P3HT STrs. a) Retention curves of RR‐b‐RRa P3HT STrs over 1000 s. b) Calculated ∆G_channel_ during 200 sec, as a function of potentiation pulse number. c) Optical energy versus absorbance differences of charge states, under gradual potentiation condition. d) Variation in charge state peak position with potentiation pulse number. e) Signature Raman peaks of trapped TFSI^−^ anions in doped RR‐b‐RRa P3HT channel. f) Schematic illustration of channel rDoC‐dependent back‐diffusion behavior of trapped TFSI^−^ anion.

Regarding the LTD condition, all of the RR‐b‐RRa P3HT STrs exhibited an abrupt and asymmetrical reduction in G_channel_ compared to the LTP curves. Although this asymmetrical depression behavior has often been observed in OECT‐STrs in previous studies, the underlying cause remains unclear. One possible explanation is the easier extraction of penetrated TFSI^−^ anions from the RR‐b‐RRa P3HT films at the interface between the active channel and ion‐gel electrolyte under a depression pulse bias, facilitated by the increase in free volume resulting from the structural deformation following TFSI^−^ infiltration under the potentiation gate pulses. This hypothesis is supported by XPS analysis, which revealed a significant decrease in TFSI^−^ anion content of the RR‐b‐RRa P3HT films after the first 10 gate pulses at +1 V (Figure S15, Supporting Information), as well as in situ electro‐spectroscopic analysis, which showed an exponential decay of polaronic and bipolaronic peak intensities under depression conditions (Figures **5**d and S13). The present findings suggest that the rDoC of the channel has a more significant effect on the LTP than on the LTD behavior. Further investigation is required to clarify and improve the NL value of the OECT‐STrs during LTD modulation.

To ensure that the RR‐b‐RRa P3HT STrs are capable of stable neuromorphic computation, we investigated the long‐term memory retention of the STrs at the specified G_channel_. Previous studies established that the decrease in the G_channel_ of an OECT‐STr arises from discharging of the channel accompanied by spontaneous back‐diffusion of doped ions and resultant charge stabilization.^[^ ^23^, ^66^ ^]^ Figure S18 and Note S7 (Supporting Information) show the state‐retention behaviors of the three RR‐b‐RRa P3HT STrs as a function of the doped states of the channel, as well as a comparison of the decay time constants. At low doped states (G_channel_ ≈0.25 mS), the [RR]_100_‐STr exhibited a smaller current decay compared to the [RR]_78_ and [RR]_63_‐based devices. This is consistent with expectations based on the ion velocity measurements shown in Figure 3d. At this early stage, polarons that weakly interact with TFSI^−^ anions through Coulombic interactions form in both the crystalline and amorphous phases. The TFSI^−^ anions can easily overcome these weak interactions and back‐diffuse, making ionic diffusion the main contributor to channel discharging.

Interestingly, the retention behavior changes dramatically as the potentiation level increases. As the number of potentiation pulses increases, the devices with a higher amorphous fraction demonstrate superior retention (Figure S18, Supporting Information). Figure 5a clearly shows that the highly doped [RR]_63_‐STr with lower rDoC exhibited more stable current retention over 1000 s, whereas the high‐rDoC [RR]_100_‐STr showed significant G_channel_ decay. Figure 5b shows plots of the change in G_channel_ (∆G) for the three RR‐b‐RRa P3HT STrs as a function of the number of potentiation pulses. As the device was programmed to a high G_channel_ with an increased number of pulses, the highly crystalline [RR]_100_‐STr displayed more significant G_channel_ decay. In contrast, the [RR]_63_‐STr with low rDoC showed a notably smaller decay, even at a high current programming level. The decay time constants of the STrs, over 1000 s were determined from the retention curves (Figure S19, Supporting Information). Given that [RR]_100_ exhibited stronger pulse‐paired integration compared to [RR]_78_ and [RR]_63_, this outcome is unexpected and highlights the complex interplay between crystallinity and charge state formation during potentiation.

To investigate the correlation between TFSI^−^ anion retention and device performance, we conducted GIWAXS and XPS analyses after programming samples with 100 pulses followed by 15 min of self‐discharge at open circuit (Figures S20 and S21, Supporting Information). GIWAXS 1D line‐cut profiles revealed that all films maintained varied d‐spacing after self‐discharge, with increased inter‐lamellar distance and reduced *π–π* stacking distance. Notably, [RR]_63_ exhibited a more pronounced structural deformation than did [RR]_100_. Complementary XPS analysis of the samples confirmed a higher residual TFSI^−^ content in samples with a greater amorphous fraction (Figure S21, Supporting Information). This implies that the enhanced ion trapping in amorphous‐rich channels is related to their improved retention characteristics at high potentiation.

To evaluate the stability of the charge states in the doped RR‐b‐RRa P3HT channel, the decay in light absorption of the charge states over time was observed during potentiation. Figure S22 (Supporting Information) shows the time‐resolved decay behaviors of the polaron and bipolaron states electrochemically generated in the RR‐b‐RRa P3HT channels. Consistent with the retention properties, [RR]_100_ showed a faster decay of polaron and bipolaron absorption compared to [RR]_63_, indicating that oxidized charge carriers were more stably maintained in the films with a higher amorphous fraction. Notably, we found that the polaron and bipolaron absorption peaks exhibited different shifting behavior as a function of the number of potentiation pulses, reflecting differences in the energy levels of the oxidized states depending on the amorphous fraction in the film. As shown in Figure 5c and d, all of the RR‐b‐RRa P3HTs exhibited a similar initial polaron energy level with a shift to lower values during potentiation. In contrast, more stable, higher‐energy bipolaron states were observed as the rDoC of the RR‐b‐RRa P3HT film decreased. It is known that the high‐energy bipolaron state is attributed to the localization of charge carriers along the polymer chain. This suggests that the stability of bipolaron states is significantly influenced by the amorphous nature of the films, as a higher amorphous fraction will lead to greater localization of bipolarons along the polymer chains.

The localization of bipolarons in the amorphous phase can be attributed to the Coulombic binding interactions between the bipolarons and the penetrated TFSI^−^ anions.^[^ ^28^, ^67^ ^]^ To investigate the molecular interactions of the TFSI^−^ anions, the Raman spectra of doped RR‐b‐RRa P3HT films were analyzed. As shown in Figure 5e, the maximum peak position of the TFSI^−^ anion shifts to a higher wavenumber as the channel rDoC decreases. This indicates that the trapped TFSI^−^ anions in low rDoC channels experience more intense Coulombic interactions, with coordination to positive charges.^[^ ^68^ ^]^ In summary, as depicted in Figure 5f, TFSI^−^ anions in the amorphous regions of the low rDoC channel can approach the polymer chains more closely. This proximity leads to the formation of strong contact ion pairs between oxidized charge carriers and counterions, thereby slowing back‐diffusion of TFSI^−^ anions from channel to electrolyte. Consequently, G_channel_ remains more stable, enhancing the retention time. These findings demonstrate for the first time that increasing the amorphous fraction in the channel enhances the memory characteristics of synaptic devices by improving the localization and stabilization of charge carriers in the amorphous regions.

To examine whether our findings can be broadly generalized, we further validated the crystallinity‐dependent OECT‐STr performance by examining RR:RRa P3HT blends. As shown in Figure S23 (Supporting Information), we prepared four blend films in which the weight fractions of RR P3HT were 100%, 80%, 60%, and 40%, and analyzed their microstructures using GIWAXS. Unlike the RR‐b‐RRa P3HT regioblock system, the L_c_ values of the crystalline domains in the blend films varied substantially, by ≈35%; however, their rDoC changed from 1 to 0.18 as the blend ratio of RR P3HT decreased from 100% to 40%. The electrical properties of the OECT‐STrs based on these blends are shown in Figure S24 (Supporting Information). Notably, the blend film‐based STrs showed a clear performance difference depending on the rDoC, consistent with the findings for the RR‐b‐RRa P3HT STrs. As the amorphous content in the active channel increased, the degree of G_channel_ modulation and dynamic range diminished. However, the linearity of the LTP curves improved significantly, with the NL value approaching the ideal value (0) and NS_eff_ exceeding 90 when rDoC approached 0.2. Furthermore, the long‐term state retention performance also improved, as shown in Figure S25 (Supporting Information). These results further confirm that incorporating a certain fraction of the amorphous phase into the channel is advantageous for achieving high‐performance synaptic devices.

**Figure 6:**
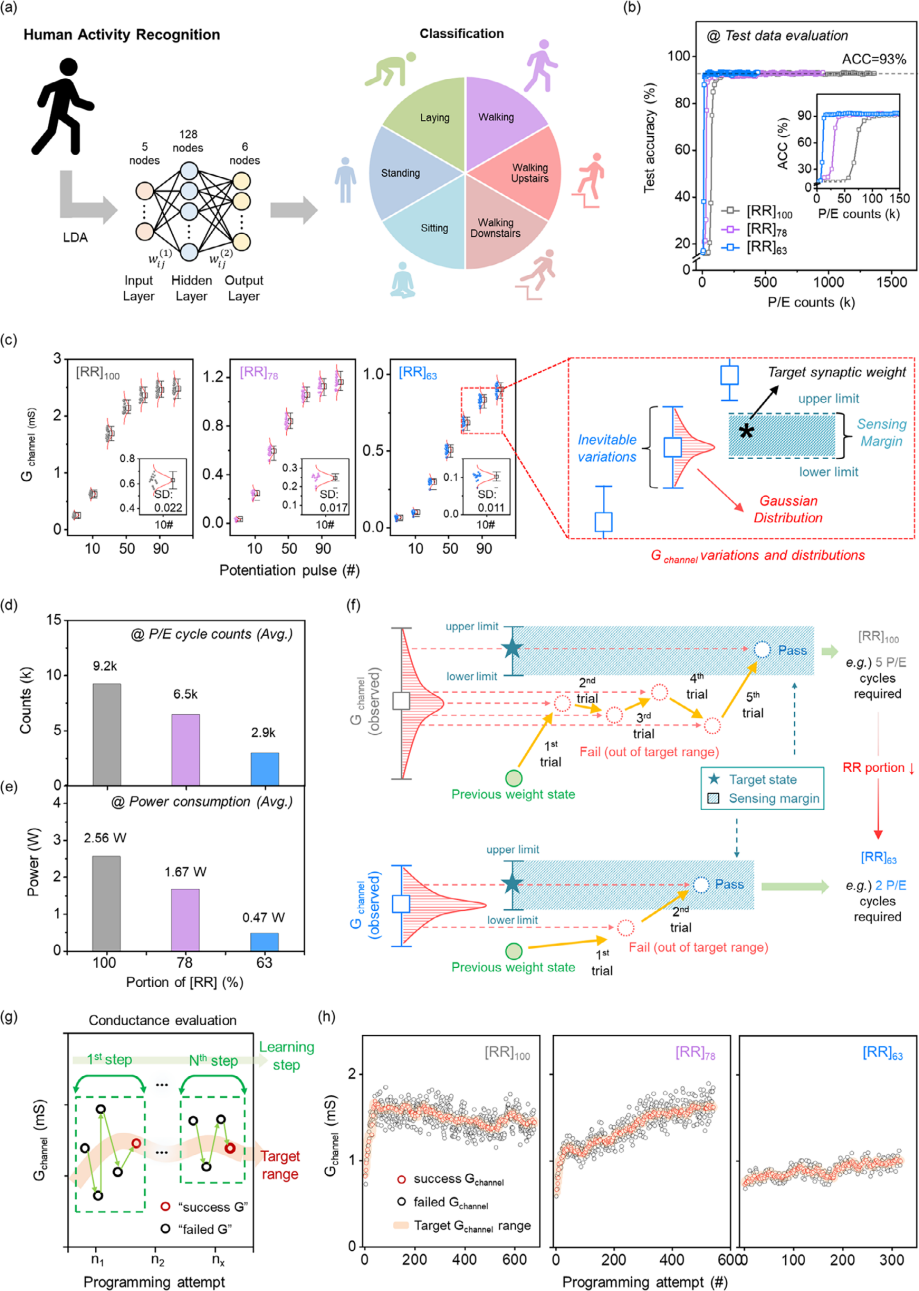
RR‐b‐RRa P3HT STrs for Deep Neural Network (DNN) applications. a) Configuration of a MLP for the HAR dataset classification using RR‐b‐RRa P3HT for synaptic weights. b) Classification accuracy of the RR‐b‐RRa P3HT STrs. c) Scatter plots of actual G _channel_ values during potentiation as a function of the number of potentiation pulses. The distribution was obtained from 15 repeated LTP/LTD measurements. The inset shows a magnified view of the data after 10 potentiation pulses, where SD denotes the standard deviation of the 15 individual G _channel_ values. Schematic of modeled target G_channel_ values with sensing margins, accounting for the characteristic variation in RR‐b‐RRa P3HT STrs. d) Reduction in program/erase cycles and e) decreased power consumption, with decreasing [RR] fraction. f) Effect of G_channel_ distribution on program/erase cycle efficiency. g) Schematic illustration of conductance evaluation procedure during neural network training, showing successful and failed programming attempts. h) Tracking of individual synaptic conductance programming attempts throughout DNN training. Black points represent failed programming attempts, while red points show successful programming attempts that achieved the target conductance within the sensing margin. The orange shaded zone indicates the target conductance ranges considering hardware sensing margins.

Through these findings, we demonstrated that crystallinity engineering, specifically by increasing the amorphous content in the channel, provides advantageous enhancements of the NL of synaptic devices with stable retention behavior, which is a key factor for neural network learning. Next, we performed simulations to explore the performance and characteristics of applications using RR‐b‐RRa P3HT STrs as synapses in deep neural networks (DNNs). As shown in Figure **6**a, a multi‐layer perceptron (MLP) was used, with the synaptic weights modeled on the properties of RR‐b‐RRa P3HT STrs. The MLP consisted of 5 input features (obtained after linear discriminant analysis (LDA) dimensionality reduction), 128 hidden nodes, and 6 output nodes to classify the UCI Human Activity Recognition (HAR) dataset.^[^ ^69^ ^]^ The HAR dataset comprises smartphone sensor data for recognizing activities such as walking, walking upstairs, walking downstairs, sitting, standing, and lying down. This dataset was preprocessed, with features normalized and reduced through LDA. The synaptic weights of the model were also clamped to a range of ‐1 to 1. The RR‐b‐RRa P3HT STrs were applied to model the synaptic weights of the MLP model. In the cross*‐b*ar array structure of RR‐b‐RRa P3HT STrs, input voltages are combined with conductance to conduct the vector‐matrix multiplication, resulting in a total current output. In a similar way, in the MLP model, inputs and weights are processed through vector‐matrix multiplication to generate output values. However, the input x of the model and the voltage input to the RR‐b‐RRa P3HT STrs belong to different domains. Hence, to align the input voltage range with the model's input range of 0 to 1, a normalization process was applied. The conductance values of the STrs, transferred as synaptic weights, were also normalized for the model simulations. This process is described by the following equation:

(2) $$y_{i}=\sum_{i}w_{ij}x_{i}\to I_{i}=\sum_{i}G_{ij}V_{i}$$

(3) $$w_{\mathrm{ij}}=2*\frac{G_{\mathrm{ij}}-G_{min}}{G_{\max}-G_{min}}-1$$

(4) $$x=\frac{V_{i}}{V_{max}}$$

To assess the model's performance using the transistor conductance values as synaptic weights, the following approach was adopted: initially, synaptic weights were updated by training on the HAR train dataset using software. The updated weights were compared with the normalized transistor conductance values to identify the nearest PSC value. These weights, selected from the transistors, were used as the weights in the MLP model, and an evaluation was subsequently conducted on the HAR test dataset. The classification accuracy obtained from this process is illustrated in Figure 6b. In tests of the conductance of the three devices with different RRa ratios, all of the devices showed test accuracies exceeding 93.0%, with [RR]_100_ achieving up to 93.31%, [RR]_78_ up to 93.41%, and [RR]_63_ up to 93.62%. Importantly, the device with the highest amorphous content ([RR]_63_) reached this accuracy level with significantly fewer programming/erase counts than the high crystallinity device ([RR]_100_). These results validate the potential of using RR‐b‐RRa P3HT STrs as synaptic weights in DNNs.

During DNN processing, it is crucial to account for the inherent variation of artificial synaptic devices when representing target synaptic weights with their corresponding conductance values. These target synaptic weights are implemented through conductance values in artificial synaptic devices, where inherent device‐to‐device variations inevitably generate deviations between target and actual conductance values. As shown in Figure 6c, the conductance, obtained from 15 repeated measurements at each conductance level, followed a Gaussian distribution characterized by specific mean and standard deviation values. Importantly, the RR‐b‐RRa P3HT STrs with higher RRa ratios exhibited narrower standard deviations and improved linearity in G_channel_. A sensing margin defines the upper and lower limits around the target G_channel_ values. To utilize the stochastically observed G_channel_, the mean and standard deviation of the G_channel_ nearest to the target G_channel_ were selected. Utilizing this mean and standard deviation, the simulated G_channel_ was stochastically generated. The synaptic weight was determined by the generated G_channel_ only if it fell within the specified sensing margin. If the newly generated G_channel_ lay outside this margin, an erase operation was triggered. This process of programming and erasing was repeated iteratively until the synaptic conductance aligned with the sensing margin. This method of transferring a single synaptic weight to synaptic conductance was repeated for all synaptic weights. Figure 6d presents the average number of P/E counts required to implement each target synaptic weight value, which was previously determined through software‐based training on the HAR train dataset, into the corresponding physical device conductance value, based on RR‐b‐RRa P3HT STrs with three different RRa fractions. Notably, the required number of P/E counts significantly decreased as the RRa ratio in the RR‐b‐RRa P3HT STrs increased. While 9.20k counts were required for the [RR]_100_ STr, the [RR]_63_‐based STr achieved the same training with only 2.97k P/E counts, less than one‐third of the counts required for the [RR]_100_ device. Furthermore, the power consumption for configuring the model weights with RR‐b‐RRa P3HT STrs can be estimated based on the calculated P/E counts. The formula for estimating the power is as follows:

(5) $$\mathrm{power}\approx V_{i}*I_{i}*P/E\;counts$$

As shown in Figure 6e, the power consumption of the [RR]_63_‐STr is only 18% of that required for the [RR]_100_‐STr, highlighting the significant energy efficiency achieved by increasing the RRa ratio. The lower power consumption by RR‐b‐RRa P3HT STrs with a higher RRa ratio is not only due to the decrease in the number of P/E counts but also because [RR]_78_ and [RR]_63_ operate at lower current compared to [RR]_100_. Figure 6f highlights the significance of controlling the standard deviations for learning accuracy and power consumption when utilizing STrs as synaptic weights in DNNs.

To obtain deeper insights into the programming efficiency, a microscopic analysis of the conductance evolution during neural network training was performed. Figure 6g illustrates the conductance evaluation procedure, demonstrating how individual conductance values evolve across the training steps. During neural network processing, each conductance update requires programming the device to a specific target conductance value within a defined sensing margin. Programming attempts that achieve conductance values within this target range are classified as “successful conductance” attempts, while those falling outside this margin are termed “failed conductance” attempts. While all device types successfully complete the same number of learning steps, the critical difference lies in the total number of programming attempts due to different numbers of “failed conductance” attempts prior to success. Figure 6h presents the detailed analysis, demonstrating that for the same training process, the [RR]_100_ and [RR]_78_ devices required 678 and 542 programming attempts, respectively, while [RR]_63_ devices needed only 317 attempts—representing a 53% reduction compared to the [RR]_100_ device.

This substantial difference in programming efficiency is directly related to the conductance variation characteristics of each device, rather than resulting from differences in conductance levels among the devices. To confirm this, we adjusted the programming pulse amplitude for each STr to reach the same G_channel_ level (∼1.3 mS) and investigated the power consumption (Figure S26, Supporting Information). Although the [RR]_100‐_ and [RR]_78_‐STrs were able to reach the target conductance with lower pulse amplitudes, the [RR]_63_‐STr exhibited the lowest power consumption (39% of that required by the [RR]_100_‐STr). To further validate this, the maximum conductance level was matched using pulse number modulation, as shown in Figure S27 (Supporting Information). Even though the number of P/E counts for the [RR]_100_‐STr decreased by ≈20% (from 9.2 to 7K) compared to the simulation results in Figure 6, the [RR]_63_‐STr consistently showed the lowest power consumption, requiring only 46% of the power needed by the [RR]_100_‐STr.

The larger standard deviation observed for the [RR]_100_‐STrs results in a sparser G_channel_, requiring more P/E trials to achieve the target G_channel_, albeit with a lower probability of success. When the probability density of the target G_channel_, is high, as for the [RR]_63_‐STr with a high RRa ratio, the likelihood of the desired conductance falling within the sensing margin increases, reducing the number of P/E counts. A similar trend in learning efficiency was observed in a more general benchmark task, the MNIST handwritten digit recognition test. As shown in Figure S28 (Supporting Information), the [RR]_63_‐STr showed clear superiority in both reduced P/E count and lower power consumption during pattern recognition. This result clearly demonstrates that the presence of an amorphous phase in the active channel of OECT‐STrs is critical to the power efficiency of these devices for DNN‐based learning applications.

## 3 Conclusion

In this work, we demonstrated the critical contribution of the amorphous phase in CP‐MIEC active channels to the synaptic properties of OECT‐STrs based on conjugated block copolymers. We showed that, by varying the length of the regiorandom segment in RR‐b‐RRa P3HTs, we could systematically modulate the rDoC without altering the geometry or orientation of the crystalline domains. This approach enabled investigation of the distinct contributions of the crystalline and amorphous phases to synaptic responses. In situ electro‐spectroscopic analysis and characterizations of OECT devices based on RR‐b‐RRa P3HT films with three different amorphous fractions revealed that a higher amorphous content enhances the ion mobility and volumetric capacitance of the channel, but lowers the charge carrier mobility in the OECT device. As a result, the device with a higher amorphous content exhibited a larger EPSC amplitude with a lower conductance level compared to the device with a content of crystalline phases. Interestingly, we observed that the linearity of LTP modulation was significantly enhanced by increasing the amorphous fraction in the RR‐b‐RRa P3HT channel. This was attributed to the gradual generation of polarons in the amorphous phase and bipolarons in the crystalline phase, which progressively induced an increase in the channel conductance. Moreover, the OECT‐STr with a high amorphous fraction exhibited improved long‐term state retention, which was attributed to the prevention of back‐diffusion of infiltrated anions and strong localization of charge carriers in the channel. To explore the generalizability of our findings, we investigated the impact of crystallinity on synaptic behaviors of OECT‐STrs based on a blend system of regioregular and regiorandom P3HTs, and found that these systems exhibit behavior analogous to that of the devices based on RR‐b‐RRa P3HT. Finally, neural network simulations using the HAR dataset confirmed that the OECT‐STrs with high amorphous fractions achieved classification accuracies exceeding 93% while consuming only 18% of the power required by the device with a high crystalline content because of improved learning efficiency. This result indicates that incorporating an amorphous phase into the active channel of OECT‐STrs is important for improving the synaptic performance and power efficiency in neural network‐based learning.

## 4 Experimental Section

### Materials

Regioregular‐block‐regiorandom P3HT copolymers (RR‐b‐RRa P3HTs) were synthesized via modified catalyst‐transfer polycondensation, adapting a literature‐reported Grignard metathesis protocol.^[^ ^70^ ^]^ The synthesis procedure is described in detail in the Supporting Information. Poly(vinylidene fluoride‐co‐hexafluoropropylene) (P(VDF‐HFP), Mn = 130 kg mol^−1^ and Mw = 400 kg mol^−1^), 1‐ethyl‐3‐methylimidazolium bis(trifluoromethylsulfonyl)imide [EMIM][TFSI], acetone, chlorobenzene, and indium tin oxide (ITO)‐coated glass were purchased from Sigma‐Aldrich.

### Characterization of the Material Properties and Doping Kinetics

The molecular composition and weight distribution of RR‐b‐RRa P3HTs were confirmed by ^1^H NMR and GPC, respectively. Crystallinity of RR‐b‐RRa P3HT films was evaluated with GIWAXS and DSC. Electrochemical doping behaviors and redox states of doped RR‐b‐RRa P3HT films were analyzed with electro‐spectroscopic measurements, including EIS, UV–Vis–NIR, XPS, and Raman spectrometers. A detailed description is provided in the Supporting Information.

### Fabrication of Synaptic Transistors (STrs) and Device Performance Characterization

Synaptic transistors were fabricated on Si/SiO_2_ substrates (300 nm oxide) with photolithographically patterned Ti/Au source and drain electrodes (5/50 nm). The channel dimensions were defined as W = 50 µm and L = 500 µm. RR‐b‐RRa P3HT thin films were formed by spin‐coating 10 mg/mL solutions in chlorobenzene. The ion‐gel electrolyte, comprising EMIM: TFSI and P(VDF‐HFP) in acetone (4:1:8 wt%), was deposited onto the channel region via drop‐casting and served as the gate dielectric. Electrical characteristics of STrs were measured under ambient conditions using a semiconductor parameter analyzer, an arbitrary function generator, and an oscilloscope. The figure‐of‐merits of STrs were calculated following the guides of previous literature.^[^ ^13^ ^]^ Further details are provided in the Supporting Information.

### Neural Network Simulation for DNN Applications

The synaptic performance of RR‐b‐RRa P3HT STrs was evaluated using a multi‐layer perceptron (MLP) model trained to classify the UCI Human Activity Recognition (HAR) dataset. Input features were reduced from 561 to 5 dimensions via Linear Discriminant Analysis (LDA) to enhance class separability and reduce computational complexity. Synaptic weights were modeled based on the measured conductance characteristics of the devices, normalized to a range of −1–1. A Gaussian distribution and sensing margin were applied to emulate device stochasticity. Performance metrics included classification accuracy, program/erase (P/E) counts, and power consumption, simulated for three representative STr configurations. Detailed experimental procedures are provided in the Supporting Information.

## Conflict of Interest

The authors declare no conflict of interest

## Supporting information



Supporting Information

## Data Availability

The data that support the findings of this study are available from the corresponding author upon reasonable request.
