# Supplementary material for: Role of Amorphous Phases in Mixed Conduction of Conjugated Regioblock Copolymers for Organic Electrochemical Synaptic Transistors
Source: Adv Mater. 2025 Aug 30;37(45):e02133. doi: 10.1002/adma.202502133 (PMC12617028; doi:10.1002/adma.202502133)
Supplement: Supplementary file 1 — Supporting Information [file ADMA-37-e02133-s001.docx]

**[Supporting Information]**

Role of Amorphous Phases in Mixed Conduction of Conjugated Regioblock Copolymers for Organic Electrochemical Synaptic Transistors

*Kwang-Hun Choi ^1, 3 †^, Seongil Im ^2, 6†^, Aaron Plant ^4^, Carlos Neri Soto ^4^, Hanna Lee ^1,6^, Changsoon Choi ^2^, Ho Won Jang ^3,7^, Jeong Ho Cho ^6^, Hyunsu Ju ^2*^, Youngmin Lee ^4*^, Jung Ah Lim ^1,5,8*^*

1. *Electronic Hybrid Materials Research Center, Korea Institute of Science and Technology, Seoul 02792, Republic of Korea*
2. *Center for Opto-Electronic Materials and Devices, Korea Institute of Science and Technology, Seoul 02792, Republic of Korea*
3. *Department of Materials Science and Engineering, Research Institute of Advanced Materials, Seoul National University, Seoul 08826, Republic of Korea*
4. *Department of Chemical Engineering, New Mexico Tech, New Mexico 87801, United States*
5. *Division of Nano and Information Technology, KIST School, Korea University of Science and Technology of Korea (UST), Seoul 02792, Republic of Korea*
6. *Department of Chemical and Biomolecular Engineering, Yonsei University, Seoul 03722, Republic of Korea*
7. *Advanced Institute of Convergence Technology, Seoul National University, Suwon 16229, Republic of Korea*
8. *Department of Materials Science and Engineering, YU-KIST Institute, Yonsei University, Seoul 03722, Republic of Korea*

*Corresponding Authors
*E-mail:* [*jalim@kist.re.kr*](mailto:jalim@kist.re.kr) / [*youngmin.lee@nmt.edu*](mailto:youngmin.lee@nmt.edu) */* [*hyunsuju@kist.re.kr*](mailto:hyunsuju@kist.re.kr)

**Experimental Section**

Synthesis of regioregular-block-regiorandom P3HT block copolymers: Regioregular-block-regiorandom P3HT block copolymers (RR-b-RRa P3HTs) were synthesized via catalyst-transfer polycondensation involving simultaneous Grignard reactions. The synthetic procedure was adapted from the literature^[1]^. All chemicals were obtained from Sigma-Aldrich except 5,5′-dibromo-3,3′-dihexyl-2,2′-bithiophene, which was purchased from TCI America.

The polymerization was conducted using two separate reactors. Reactor 1 contained 0.50 g (1.53 mmol, 0.33 mL) of 2,5-dibromo-3-hexylthiophene (HT monomer), while Reactor 2 contained a mixture of 0.32 g (0.99 mmol, 0.21 mL) HT monomer and 0.24 g (0.50 mmol, 0.18 mL) 5,5′-dibromo-3,3′-dihexyl-2,2′-bithiophene (BT monomer). To synthesize block copolymers with extended RRa segments, the quantities in Reactor 2 were doubled to include 0.64 g (1.98 mmol, 0.42 mL) HT monomer and 0.48 g (1.0 mmol, 0.36 mL) BT monomer, while Reactor 1 compositions remained unchanged. Reactor 1 was designed to form the first regioregular (RR) block, while Reactor 2 facilitated chain extension to create the second regiorandom (RRa) block. The formation of regiorandom configurations through HT and BT monomer combination was verified by ¹H NMR spectroscopy (data not shown) in preliminary experiments prior to block copolymer synthesis.

Both reactors were degassed under vacuum and backfilled with nitrogen. Grignard reagent (0.95 equivalents of isopropyl magnesium chloride lithium chloride complex, 1.3 M in THF) was added dropwise at 0 °C. After 1 hour, 5.3 mL of dry THF was added to Reactor 1, followed by the addition of 11 mg catalyst ([1,3-bis(diphenylphosphino)propane]dichloronickel(II)) to initiate polymerization of the first RR block. After an additional hour, 4 mL (7.5 mL for extended RRa blocks) of dry THF was added to Reactor 2, and subsequently, the contents of Reactor 2 were transferred to Reactor 1 to enable growth of the second RRa block. Following another hour of reaction (overnight at 40 °C for extended RRa blocks), the reaction mixture was quenched by precipitation in methanol. The crude product was purified via Soxhlet extraction with methanol. As a control, P3HT homopolymer was synthesized using an analogous procedure, with additional purification steps involving sequential Soxhlet extractions with methanol, acetone, and hexane.

**Materials:** Poly(vinylidene fluoride-co-hexafluoropropylene) (P(VDF-HFP), Mn = 130 kg mol⁻¹, Mw = 400 kg mol⁻¹), 1-ethyl-3-methylimidazolium bis(trifluoromethylsulfonyl)imide ([EMIM][TFSI]), acetone, chlorobenzene, and indium tin oxide (ITO)-coated glass substrates were purchased from Sigma-Aldrich. Gold microfibers (diameter: 50 μm) were obtained from Alfa Aesar. RR-b-RRa P3HT solutions were prepared by dissolving the polymers in chlorobenzene (10 mg/mL) under continuous stirring at 30°C for 3 hours. An ion-gel electrolyte was prepared by combining [EMIM][TFSI], P(VDF-HFP), and acetone in a 4:1:8 weight ratio. Before deposition, the ion-gel solution was stirred at 60°C for 12 hours.

**Material characterization:** The molecular weights and polydispersity indices of RR-b-RRa P3HTs were determined by gel permeation chromatography (GPC). Copolymer compositions were characterized using ¹H nuclear magnetic resonance (NMR) spectroscopy in deuterated chloroform (CDCl₃). Molecular aggregation behavior was investigated in both solution and solid states using UV-Vis-NIR spectroscopy (Lambda 750, PerkinElmer). Crystalline domain geometry and relative degree of crystallinity (rDoC) were analyzed using synchrotron radiation at beamline 3C of Pohang Accelerator Laboratory (PAL), Republic of Korea. Two-dimensional grazing-incidence wide-angle X-ray scattering (2D-GIWAXS) measurements were conducted using 10.35 keV X-rays at a fixed incident angle of 0.11°. Thermal transitions were analyzed using differential scanning calorimetry (DSC 4000, Perkin Elmer).

**Electrochemical analysis:** Electrochemical impedance spectroscopy (EIS) was performed using a potentiostat system (VersaSTAT 3, Ametek) in the frequency range of 10⁻¹ to 10⁴ Hz with an AC amplitude of 50 mVRMS. The capacitance (C) of block copolymer films was extracted from the impedance measurements using the equation:

*C = 1 / (2π f Z ^img^)*

where Z^img​^ (Ω) is the imaginary component of the impedance and f is the frequency (Hz). Volumetric capacitance (C*) was calculated by normalizing the capacitance to the active region dimensions.

The evolution of charge states was monitored via in situ electrospectroscopic analysis using UV-Vis-NIR and Raman spectroscopy (InVia Raman Microscope, Renishaw). A custom two-electrode electrochemical cell was fabricated as follows: RR-b-RRa P3HT films (thickness: 50 nm) were spin-coated onto ITO/glass substrates, followed by ion-gel deposition and attachment of two gold microfibers to the ITO and ion-gel layers as ground and working electrodes, respectively. Spectral changes under various bias conditions were analyzed while applying electrical potentials via a function generator (AFG3021, Tektronix) to induce electrochemical reactions between RR-b-RRa P3HT and the electrolyte.

Ionic mobility within RR-b-RRa P3HT films was analyzed using moving front experiments monitored by hyperspectral imaging. A hyperspectral camera (Snapscan VNIR, IMEC) captured electrochromic behavior in the 470-900 nm range, with data converted to single-wavelength images at 800 nm using specialized software (HSI Studio, IMEC). For ex situ analysis, RR-b-RRa P3HT films were electrochemically doped and subsequently analyzed by 2D-GIWAXS and X-ray photoelectron spectroscopy (XPS, Nexsa, Thermo Fisher Scientific, Al-Kα X-ray source at 1486.6 eV) after removing the ion-gel layer.

**Fabrication and performance characterization of RR-b-RRa P3HT synaptic transistors (STrs):** Source and drain electrodes consisting of Ti/Au (5/50 nm thickness) were photolithographically patterned and deposited onto cleaned Si/SiO_2_ substrates (300 nm oxide layer). The channel dimensions were defined as 50 μm width (W) and 500 μm length (L). RR-b-RRa P3HT solutions were spin-coated onto the substrates at controlled speeds of 1000-1500 rpm under ambient conditions to form 50 nm thick semiconducting channels. The ion-gel electrolyte was then drop-cast onto the defined channel region and dried in a vacuum chamber for 12 hours.

Electrical characterization of RR-b-RRa P3HT STrs was performed in a shielded probe station (MSTECH, M5VC) using a semiconductor parameter analyzer (Agilent 4155B) under ambient conditions. The gate electrode was positioned on the ion-gel layer, while source and drain electrodes contacted the Ti/Au pads. Transfer characteristics in both channel and electrolyte layers were analyzed by measuring currents from the respective electrodes. Gate voltage (V_GS_) sweep rates were varied from 0.5 to 5 V/s using device measurement software (Keysight).

Synaptic responses were recorded at the drain electrode while applying customized pulse sequences to the gate electrode using a function generator. Both long-term potentiation/depression (LTP/LTD) behaviors and retention characteristics were monitored under resting conditions with zero gate bias. The nonlinearity (NL) value of LTP curves was calculated using the following equations:^[2]^

*G_i_ = (G_max_ - G_min_) / (1-e^-vP^)*

*G_LTP_ = G_min_ + G_i_(1-e^-vP^)*

where G_max_ and G_min_ represent the maximum and minimum channel conductance, G_i_ is a fitting parameter, P is the number of potentiation pulses, and v denotes the nonlinearity factor. The effective number of states (N_Seff_) was determined by counting states with ΔG_channel_ above the noise threshold (0.5% of the dynamic range).

**Neural network simulation:** The performance of RR-b-RRa P3HT STrs as synaptic devices was evaluated using a multilayer perceptron (MLP) model to simulate deep neural networks (DNNs). The MLP architecture consisted of an input layer with 5 nodes (features obtained after Linear Discriminant Analysis dimensionality reduction), a hidden layer with 128 nodes, and an output layer with 6 nodes for classifying the UCI Human Activity Recognition (HAR) dataset.

The HAR dataset comprises smartphone sensor data collected from subjects performing six different activities: walking, walking upstairs, walking downstairs, sitting, standing, and lying down. These activities were recorded using embedded accelerometers and gyroscopes of smartphones attached to the subjects' waists. The original dataset contained 561 features extracted from raw sensor data, including time-domain features (mean, standard deviation, median absolute deviation) and frequency-domain features (frequency band energies, spectral entropy). Through Linear Discriminant Analysis (LDA), these 561 dimensions were reduced to 5 discriminative features while preserving the classification information necessary for activity recognition.

LDA was applied to reduce the original high-dimensional feature space to 5 dimensions while maximizing class separability. LDA projects data onto a lower-dimensional space by maximizing the ratio of between-class variance to within-class variance, thereby enhancing classification performance. This dimensionality reduction technique was particularly suitable for this application as it preserved discriminative information needed for activity recognition while significantly reducing computational requirements.

Synaptic weights were modeled based on the conductance properties of RR-b-RRa P3HT STrs, with values normalized to a range of -1 to 1. To account for device stochasticity, a Gaussian distribution was applied to conductance values with defined sensing margins around target values. The simulation incorporated a weight update mechanism during training while maintaining weight values within the normalized range. Performance evaluation included calculation of classification accuracy on the HAR test dataset, estimation of program/erase (P/E) cycle requirements for weight configuration, and analysis of power consumption. Three different RR-b-RRa P3HT STr configurations, represented by distinct device characteristics, were simulated to compare their performance in terms of accuracy, P/E cycles, and power efficiency.

Note S1: Characterization of the regioregularity of synthesized RR-b-RRa P3HTs

Formation of block copolymers was confirmed by GPC traces in Figure S1. Compared to the RR block collected from Reactor 1 before combining with Reactor 2, the GPC traces of block copolymers, [RR]₇₈ and [RR]₆₃, are shifted toward shorter retention times, indicating chain extension from the first RR block. P3HT homopolymer, [RR]₁₀₀, exhibits a narrower dispersity, which is attributed to additional cycles of Soxhlet extraction. Molar masses and dispersity determined by GPC are listed in Table S1.

The composition of RR-b-RRa P3HTs was analyzed by ¹H NMR in deuterated chloroform (CDCl₃) solution. The regioregularity of [RR]₁₀₀ was first estimated as 94% by comparing the α-CH₂ peak from the regioregular configuration at 2.8 ppm with that from the regio-irregular configuration at 2.6 ppm in Figure S2(a). Subsequently, the composition of RR-b-RRa P3HTs was estimated by comparing the α-CH₂ peak at 2.8 ppm from the RR block with that from the RRa block at 2.6 ppm in Figures S2(b) and (c). When the integration areas of the two peaks were compared, the RR block was assumed to have the same regioregularity as [RR]₁₀₀. As a result, the respective RR-b-RRa P3HTs have 78:22 and 63:37 ratios of [RR]:[RRa]. For simplicity, these are denoted as [RR]₇₈ and [RR]₆₃.


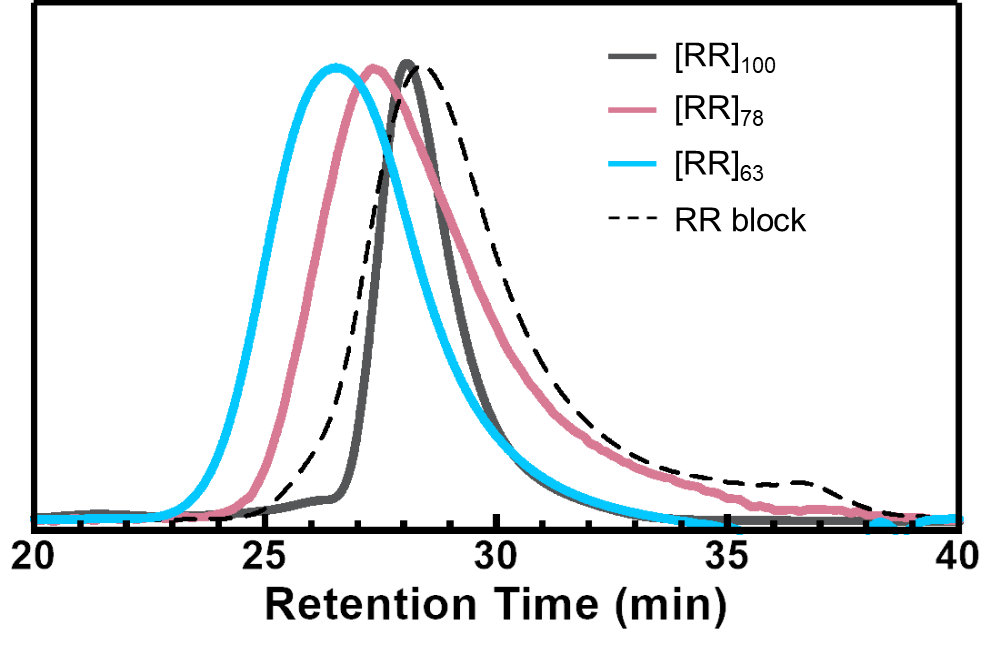


Figure S1. GPC traces of RR-b-RRa P3HT samples (solid lines) and the first block (RR Block) during block copolymer preparation (dashed line).

Table S1. Molar masses of RR-b-RRa P3HT samples

| Material | M_n_ (kg/mol) | M_w_ (kg/mol) | Ð |
| --- | --- | --- | --- |
| [RR]_100_ | 14.1 | 16.2 | 1.15 |
| [RR]_78_ | 14.3 | 21.8 | 1.52 |
| [RR]_63_ | 17.1 | 26.7 | 1.56 |
| RR Block | 8.2 | 13.2 | 1.61 |


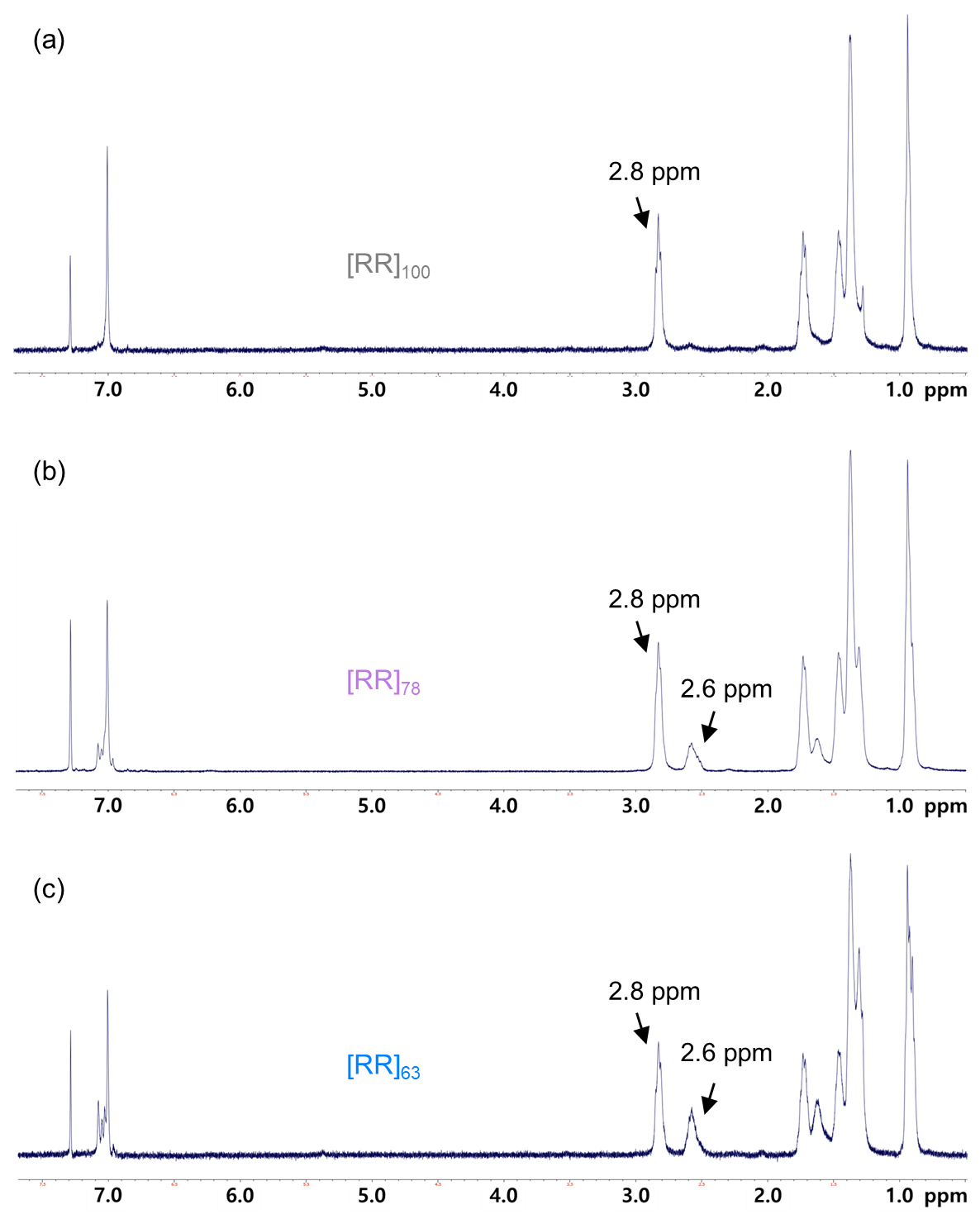


Figure S2. ^1^H NMR spectra of RR-b-RRa P3HT in CDCl_3_: (a) [RR]_100_, (b) [RR]_78_, and (c) [RR]_63._

*
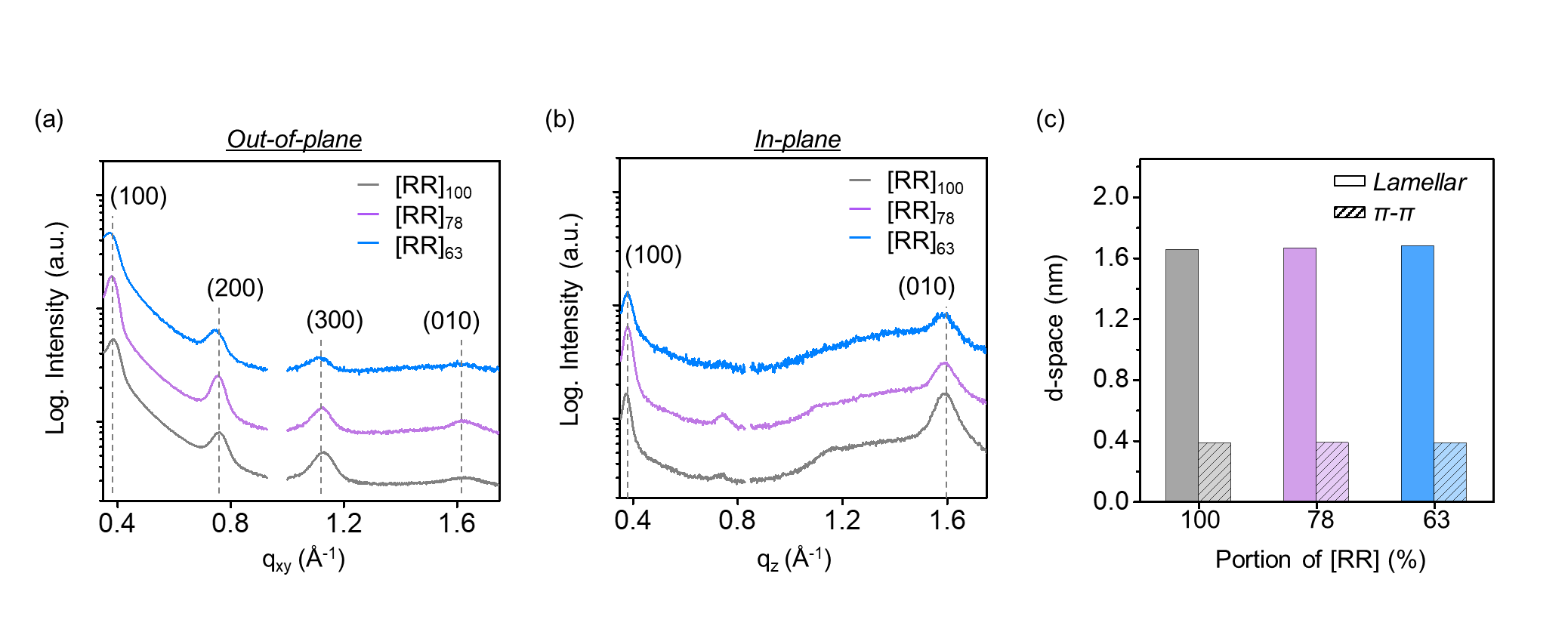
*

Figure S3. (a) Out-of-plane and (b) in-plane directional 1D line-cut profiles from GIWAXS result. (c) Lamellar spacing and π-π stacking distance for RR-b-RRa P3HT thin films.

Note S2: Analysis of the rDoC of RR-b-RRa P3HT films by DSC heating thermograms

To investigate the enthalpy for thermal transitions of crystalline domains, DSC heating thermograms were deconvoluted in Figure S4 after extracting baselines from the melting endotherms. For all samples, bimodal peaks appeared during thermal transitions. This is a well-known phenomenon called the melting-recrystallization-melting process of semi-crystalline polymers.^[3-4]^. Recrystallization occurs in a portion of the chains during the broad melting process near the first melting temperature. Melting of the recrystallized portion appears as the second melting peak (T_m,2nd_) at higher temperatures.

The overall enthalpy of melting was estimated as 2.35, 1.23, and 0.52 J/g for [RR]₁₀₀, [RR]₇₈, and [RR]₆₃, respectively. The rDoC was estimated by the ratio of the estimated values to that of [RR]₁₀₀ as the standard: 0.57 for [RR]₇₈ and 0.22 for [RR]₆₃.

For further analysis, the overall melting peak was deconvoluted by Gaussian fitting, and the discrete melting peaks were then integrated. The relative areas of discrete melting peaks (i.e., the ratio of the second peak to the first peak, Area,_2nd_ /Area,_1st_) were compared among the samples. [RR]₁₀₀, [RR]₇₈, and [RR]₆₃ present comparable values of 0.21, 0.22, and 0.21, respectively. This result implies that the partial melting and recrystallization behavior of the crystalline phase exhibits similar characteristics in all RR-b-RRa P3HT samples, regardless of RRa block length.

*
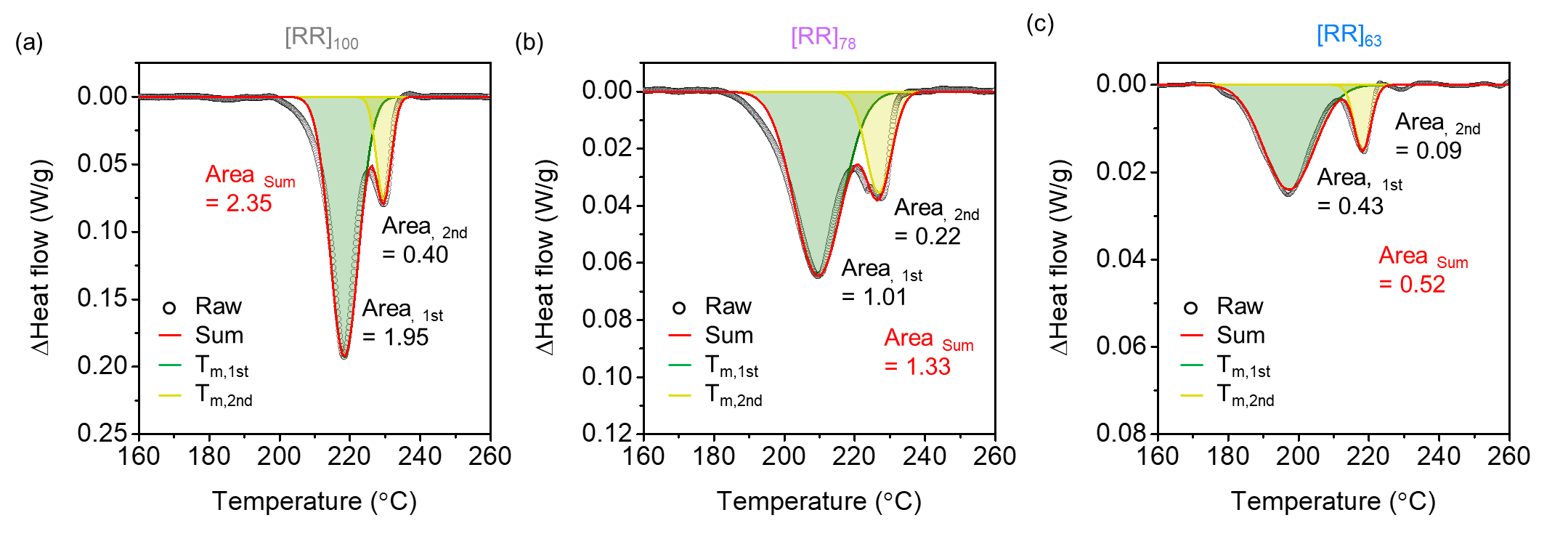
*

Figure S4. DSC thermograms (Raw) of RR-b-RRa P3HT samples and their deconvolution results: (a) [RR]_100_, (b) [RR]_78_, and (c) [RR]_63_, respectively. The enthalpy of each thermal transition was calculated by integration of the melting peaks

*
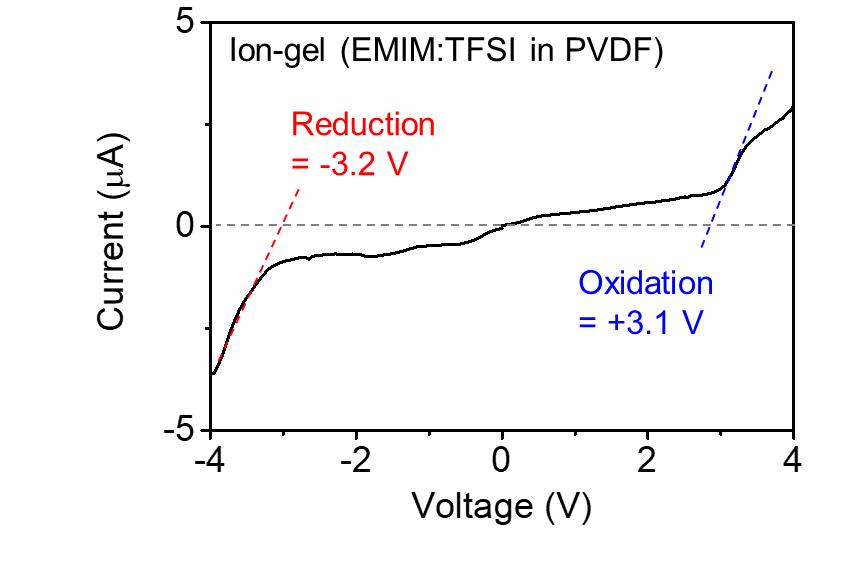
*

Figure S5. An example I-V curve of the ion-gel in the range of ±4 V.

*
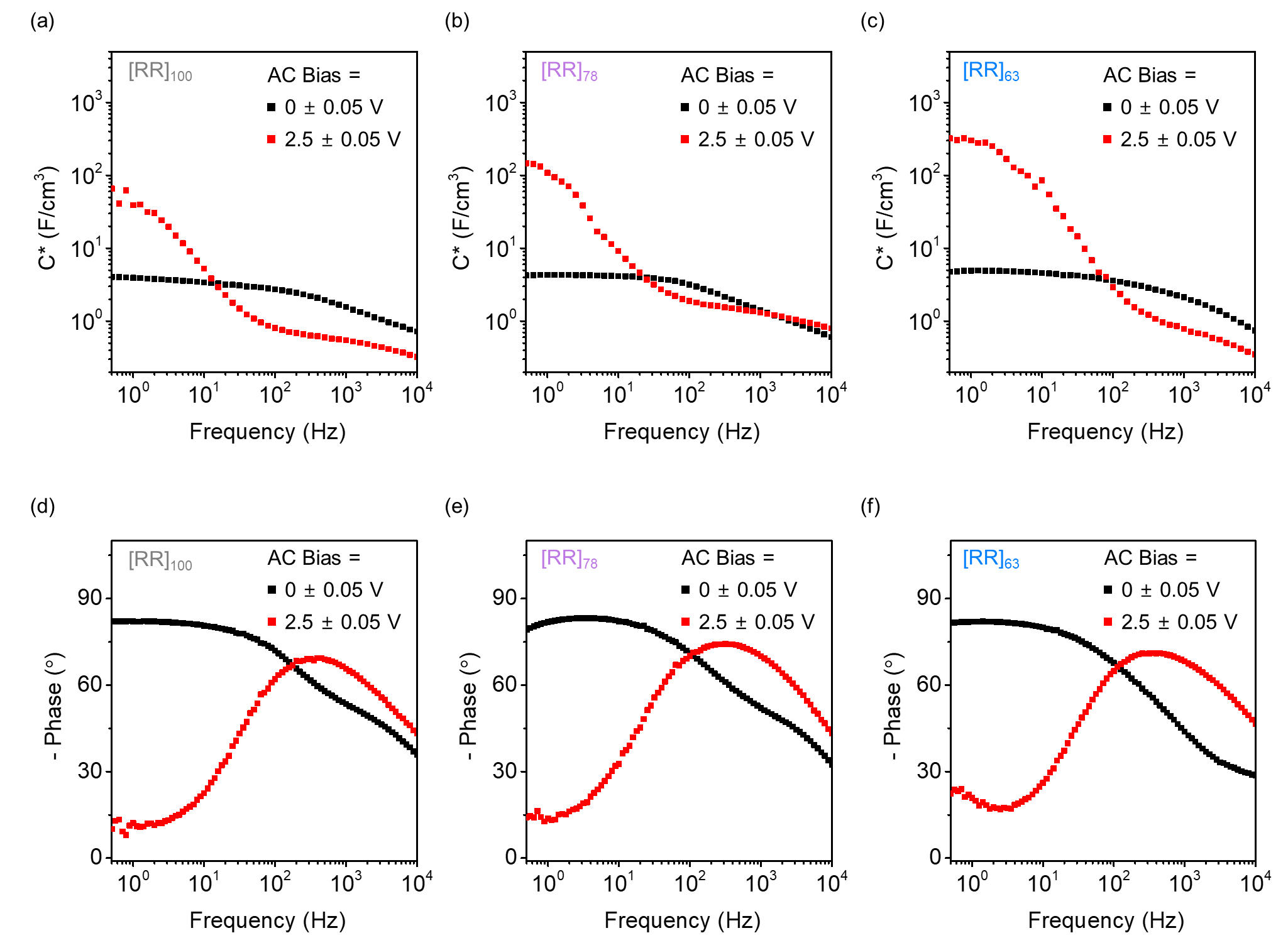
*

Figure S6. Calculated volumetric capacitances (a-c) and phase diagrams (d-f) as a function of frequency, regenerated from EIS spectroscopic results.

*
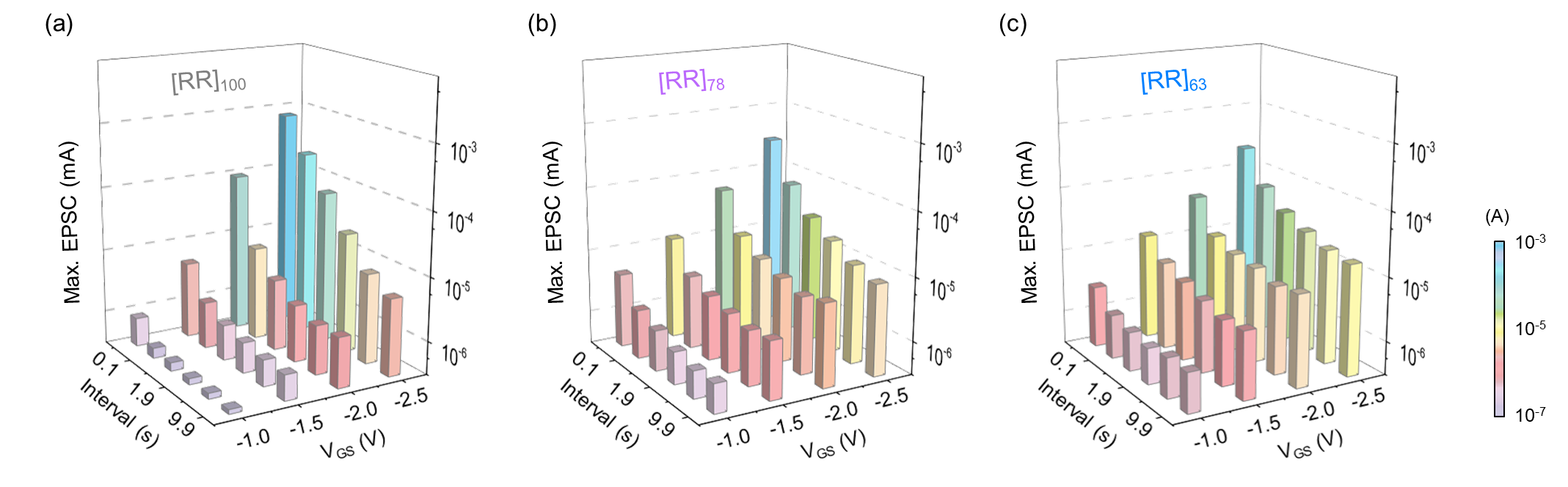
*

Figure S7. Maximum EPSC values of (a) [RR]₁₀₀-, (b) [RR]₇₈-, and (c) [RR]₆₃-based STrs under 25 s potentiation conditions, with varying presynaptic pulse strengths (-1 to -2.5 V) and pulse interval times (0.1 to 9.9 s).

Table S2. STP-to-LTP switching trends in RR-b-RRa P3HT STrs, as a function of presynaptic input potential and pulse interval time

*
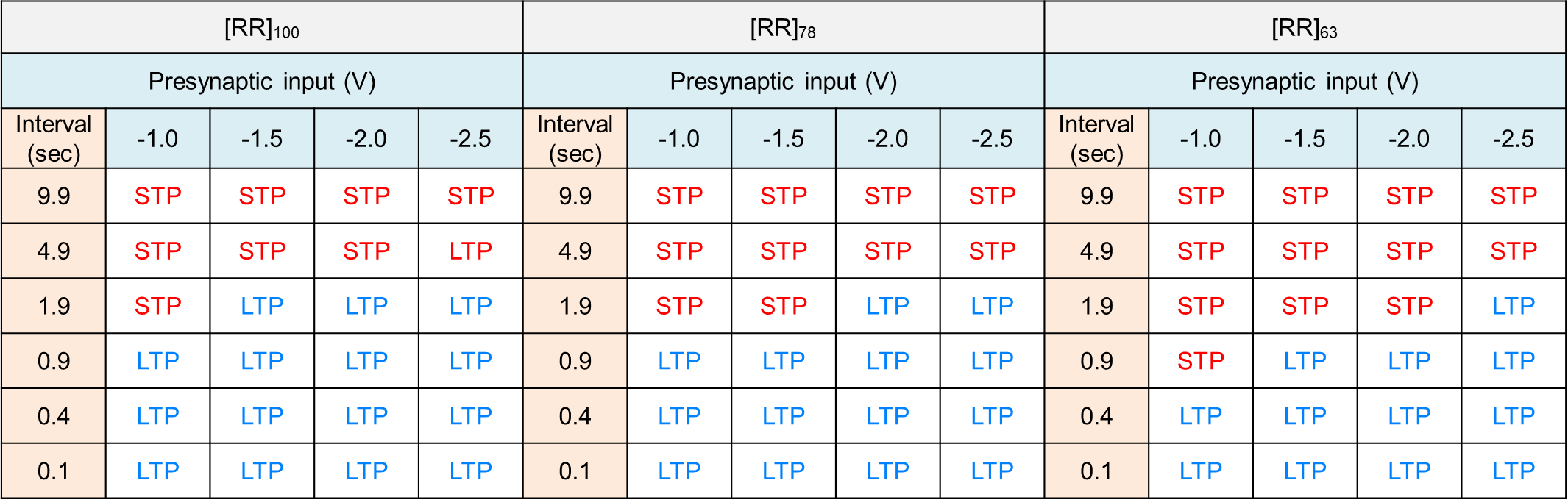
*

Note S3: Moving front experiment with hyperspectral imaging camera/software

Figure S8a shows the analysis setup for investigating ionic mobility using a hyperspectral imaging system. First, we captured 2048 × 2048 resolution images with a hyperspectral camera and extracted 800 nm absorbance images using the program (HSI Studio, IMEC). Through proportional calculations between full-size images and sample dimensions, we specified the pixel dimension as 44 μm. The magnified images (right) show three assigned regions (blue, green, and red squares) that exhibit distinct spectral differences as designated by the program. Prominent 800 nm absorbance was observed in region 1 (blue), which arose from electrochemical oxidation of RR-b-RRa P3HTs, while it gradually decreased with increasing distance from the electrode. Comparably weak 800 nm absorbance was observed in the boundary region (region 2, green), and it was not found in region 3 (red).

Figure S8b illustrates the TFSI⁻ anion drift mechanism in RR-b-RRa P3HT samples. The movement of ionic species in the polymer channel is primarily governed by applied bias, which induces ionic drift from the electrolyte toward the polymer channel. In this experiment, a negative bias (-2.5 V) was applied to the electrode where the ion-gel was deposited. As a result, TFSI⁻ anions became depolarized and began drifting through the ion-reservoir channel (RR-b-RRa P3HT) under the influence of the electric field. Since the ion-gel and RR-b-RRa P3HT channel form a lateral junction, TFSI⁻ anions drift from regions near the ion-gel toward more distant areas within the channel. However, their drift behavior is strongly influenced by the microstructure of the RR-b-RRa P3HT channel, as densely packed crystalline phases create structural barriers that hinder ion transport. In contrast, amorphous phases provide less resistance to ion drift, allowing ions to bypass these crystalline obstacles more easily. As TFSI⁻ anions travel through the channel, the applied electric field induces electrochemical oxidation of the polymer, leading to a distinct absorption peak at 800 nm.

To characterize sample-scale absorbance transitions, we analyzed line-cut images along the x-axis (black line in image) and profiled absorbance differences (ΔAbs₈₀₀ₙₘ) of respective pixels. The plots in Figure S8c present ΔAbs₈₀₀ₙₘ versus distance depending on bias application time. The ΔAbs₈₀₀ₙₘ of respective pixels was obtained by subtracting the absorbance at pristine conditions. With increasing bias application time from 0 to 10 min, ΔAbs₈₀₀ₙₘ expands across the sample. Bias application time-dependent ion (TFSI⁻ anion) drift distance was analyzed by extrapolating the slope of drastic ΔAbs₈₀₀ₙₘ changes and extracting x-intercepts. Summarized results and calculated ionic velocities of RR-b-RRa P3HT samples are plotted in Figure S8d.


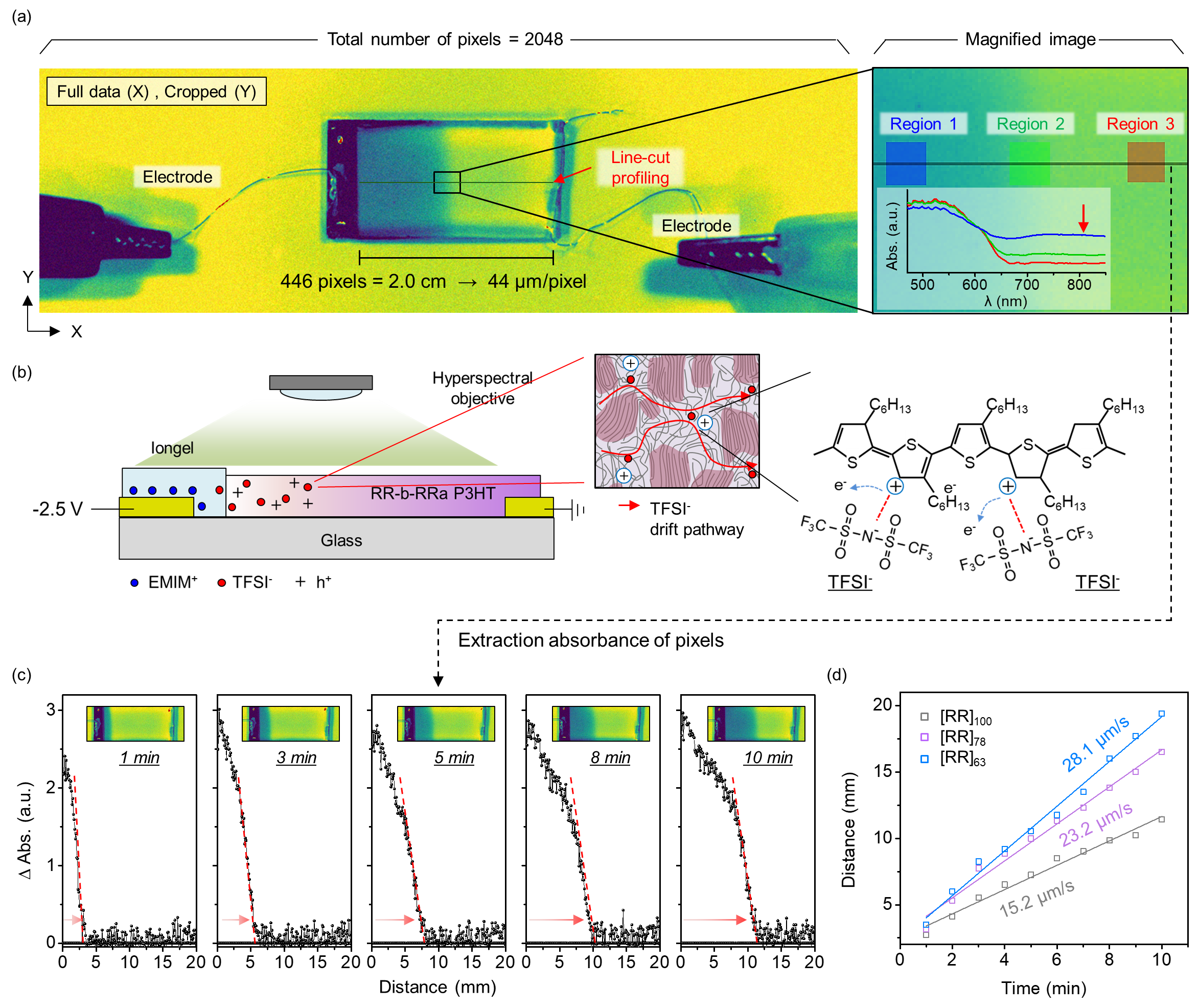


Figure S8. (a) Hyperspectral analysis setup with distance quantification method (left). Inset spectra from magnified images show absorbance differences between assigned regions (right). (b) Cross-sectional schematic illustration of moving front experiment sample and setup system; magnified illustration depicts ion drift pathways and electrochemical doping behavior in RR-b-RRa P3HT channels. (c) Regional ΔAbs at 800 nm for RR-b-RRa P3HT samples as a function of bias application time. (d) Distance versus bias application time plots and calculated ionic velocities for RR-b-RRa P3HTs.

*
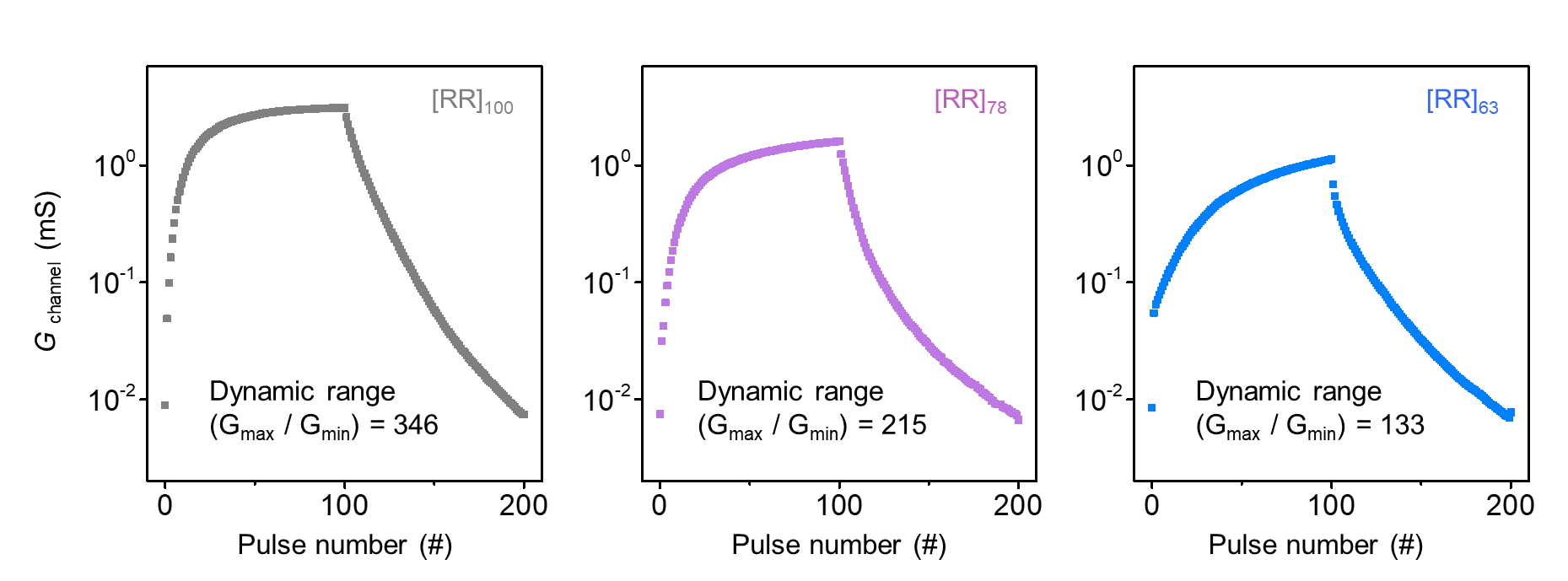
*

Figure S9. Log-scale LTP/LTD curves of RR-b-RRa P3HT STrs and calculated dynamic range.


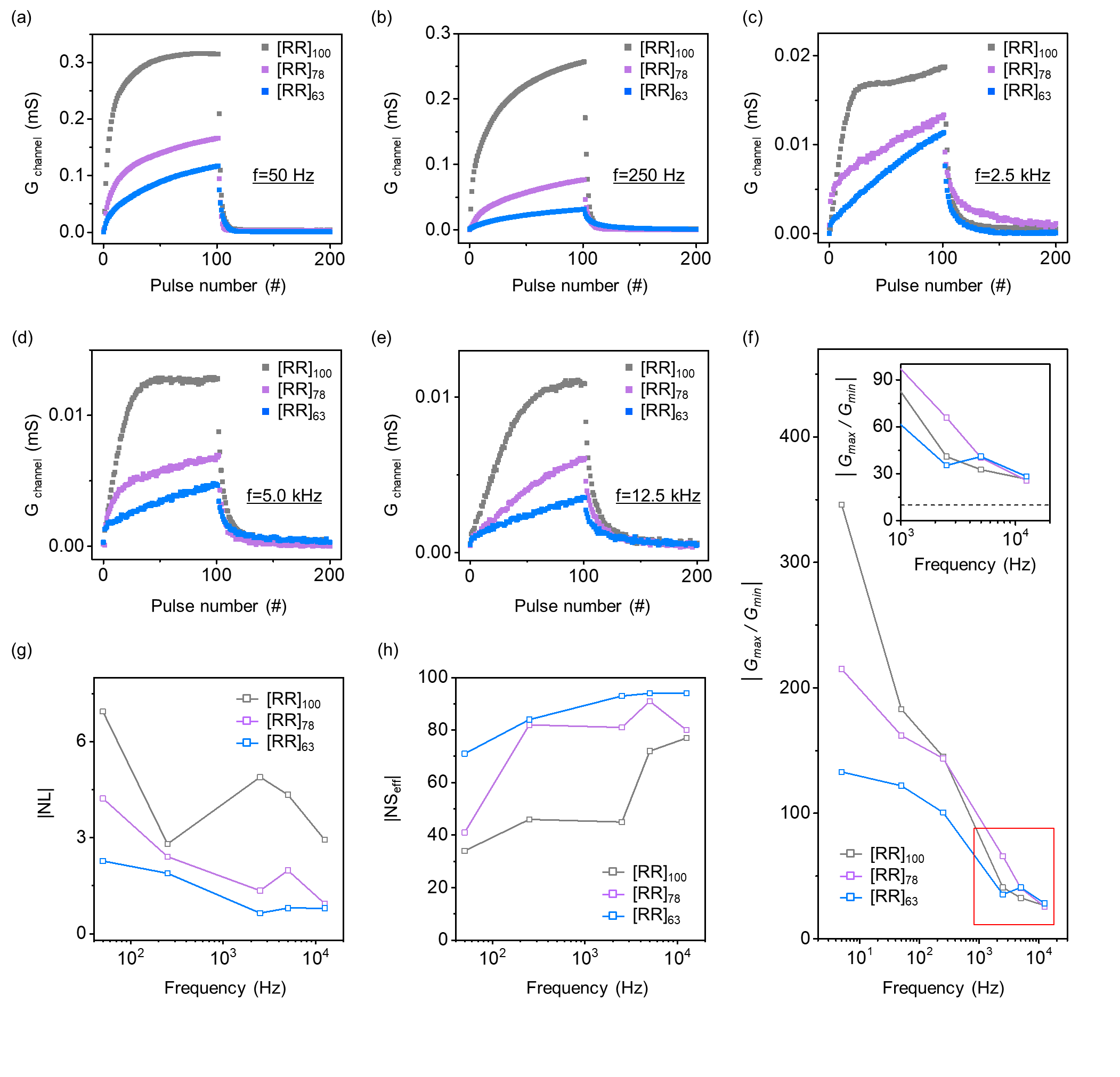


Figure S10. (a-e) LTP/LTD curves of RR-b-RRa P3HT-based STrs with varying programming/erasing pulse rates from 50 Hz to 12.5 kHz (programming pulse width from 10 ms to 40 μs). The amplitudes of programming and erasing pulses were set to -2.5 V and +1.0 V, respectively. (f) Programming pulse rate-dependent dynamic range (Gₘₐₓ/Gₘᵢₙ) of RR-b-RRa P3HT STrs. The inset plots show a magnified view of the 2.5 kHz to 12.5 kHz range. The horizontal dotted line indicates a value of 10. (g) Calculated nonlinearity (NL) values and (h) effective number of states (NSₑ_ff_) in potentiation curves, both plotted as functions of programming pulse rate.

**Note S4. Device performance comparison under identical maximum conductance**

To ensure a fair and rigorous comparison of synaptic performance across RR-b-RRa P3HT STrs, we conducted two independent sets of experiments in which the maximum conductance was aligned across devices by modulating the potentiation input conditions.

First, we fixed the pulse number (100/100 for potentiation/depression) and width (100 ms) while adjusting the potentiation pulse amplitude to tune the final conductance level. As shown in Figure S11a, this allowed us to achieve comparable maximum conductance values (~1.3 ± 0.1 mS) using -2.2 V for [RR]₁₀₀-STr, -2.4 V for [RR]₇₈-STr, and -2.5 V for [RR]₆₃-STr. The corresponding LTP/LTD curves (Figure S11b) showed notable differences in potentiation linearity, despite similar conductance ceilings. Specifically, [RR]₆₃-STr exhibited the smoothest and most gradual conductance modulation. Extracted device performance parameters (Figures S11c-d) showed that [RR]₆₃-STr had the lowest nonlinearity (NL = 0.98) and the highest effective number of states (NS_eff_ = 85), outperforming [RR]₇₈-STr (NL = 1.99, NS_eff_ = 76) and [RR]₁₀₀-STr (NL = 3.61, NS_eff_ = 56).

To further test the generality of these trends, we performed a second experiment by fixing the pulse amplitude (-2.5 V for potentiation, +1.0 V for depression) and number (100/100), while varying the pulse width to achieve equivalent maximum conductance levels. Based on results shown in Figure S10 (Supporting Information), representative curves achieving ~12.5 ± 0.8 μS were selected and plotted in Figure S12a. As before, [RR]₆₃-STr demonstrated the most linear and gradual potentiation, while [RR]₁₀₀- and [RR]₇₈-STrs displayed steeper, more nonlinear conductance changes. The extracted values of NL and NS_eff_ (Figures S12b-c) again confirmed the consistent trend: [RR]₆₃-STr showed superior performance (NL = 0.65, NS_eff_ = 82), followed by [RR]₇₈-STr (NL = 1.84, NS_eff_ = 77) and [RR]₁₀₀-STr (NL = 4.34, NS_eff_ = 62).

These results clearly demonstrate that even when maximum conductance is equalized using different pulse control strategies, the synaptic characteristics—specifically NL and NS_eff_ —are dominantly governed by the intrinsic microstructural properties of the channel material and their electrochemical doping kinetics.


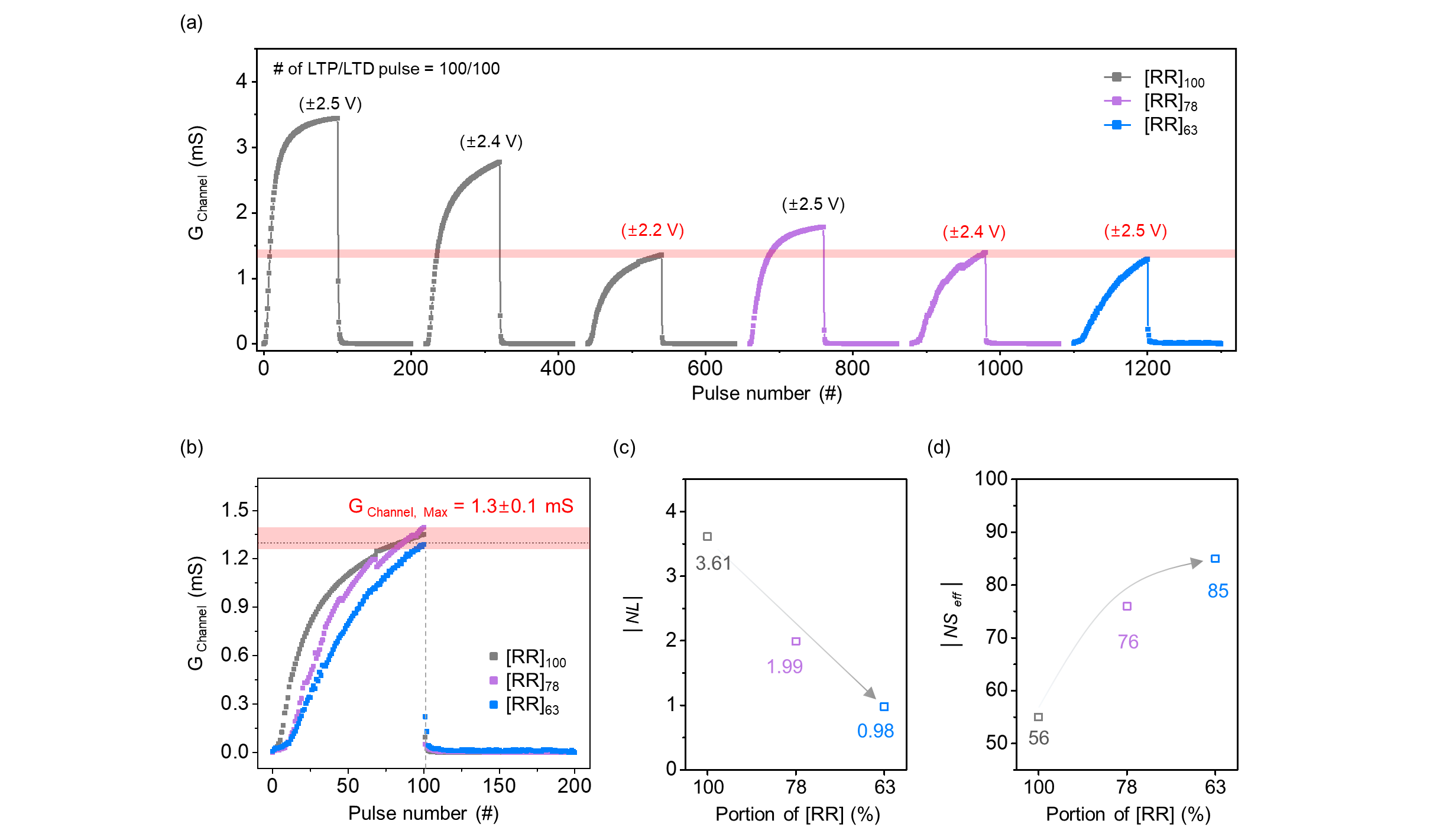

Figure S11. Pulse amplitude-dependent RR-b-RRa P3HT STr performance with fixed maximum conductance states. (a) Pulse amplitude-dependent LTP/LTD characteristics of RR-b-RRa P3HT STrs. [RR]₁₀₀-, [RR]₇₈-, and [RR]₆₃-STrs were potentiated and depressed using ±2.2 V, ±2.4 V, and ±2.5 V, respectively, with maximum G_channel_ set to 1.3 ± 0.1 mS. (b) LTP/LTD curves of RR-b-RRa P3HT STrs. (c) Extracted nonlinearity (NL) values and (d) effective number of states (NS_eff_) in potentiation curves of devices.


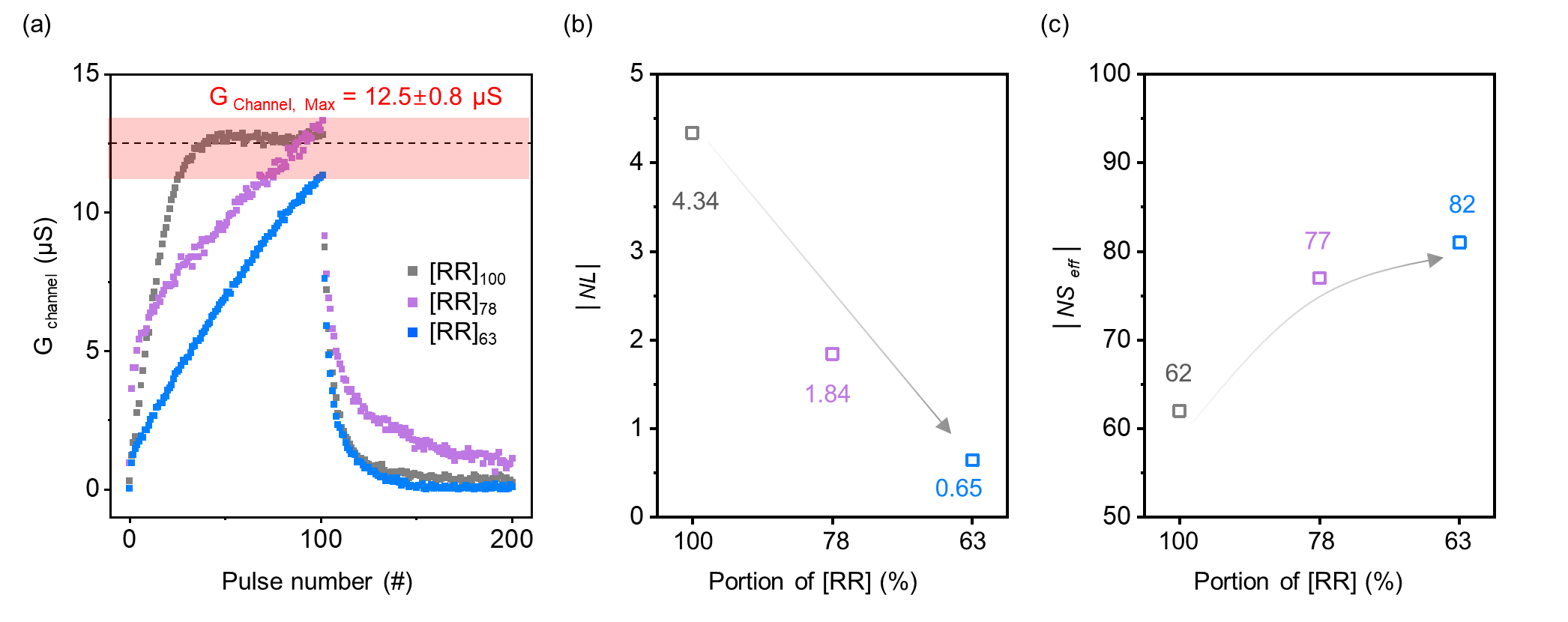


Figure S12. Pulse width-dependent RR-b-RRa P3HT STr performance with fixed maximum conductance states. (a) LTP/LTD curves of RR-b-RRa P3HT STrs with maximum G_channel_ set to 1.25 ± 0.8 μS. The pulse width for [RR]₁₀₀-STr was 100 μs, while that for [RR]₇₈- and [RR]₆₃-STrs was 200 μs at a fixed pulse amplitude of -2.5 V. (b) Extracted nonlinearity (NL) values and (c) effective number of states (NS_eff_) in potentiation curves of the devices.

*
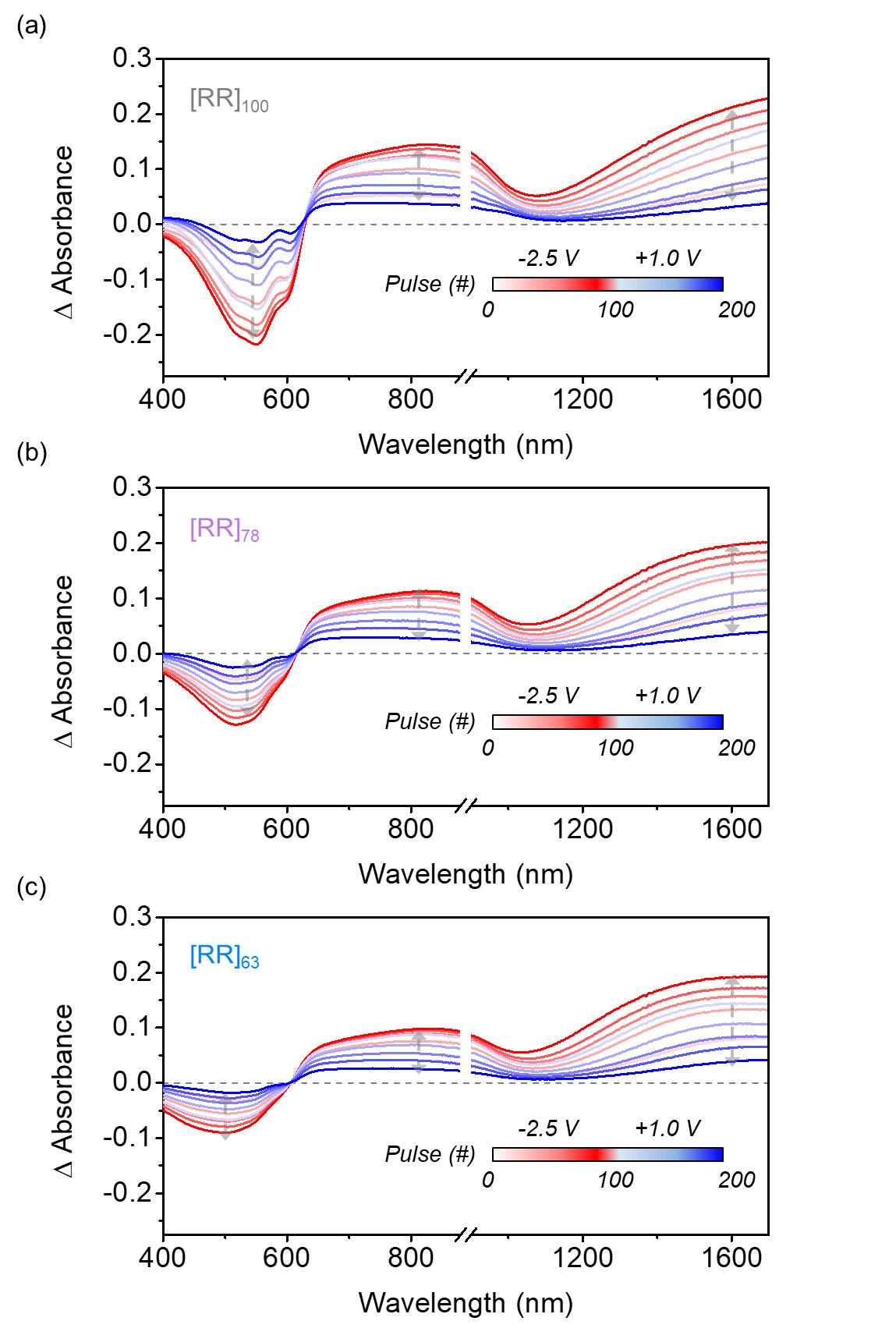
*

Figure S13. UV-Vis-NIR spectrum variations for (a) [RR]_100_, (b) [RR]_78_, and (c) [RR]_63_, respectively. Electro-spectroscopic analysis was conducted under ex situ potentiation and depression bias conditions.

**Note S5: Tracing the doping condition depending on the amount of TFSI^-^ anion trapped in RR-b-RRa P3HT channels**

Since infiltrated TFSI⁻ anions lead to oxidative doping of RR-b-RRa P3HT layers, the amount of TFSI⁻ anions in the channel needs to be investigated during potentiation and depression stimuli by X-ray photoelectron spectroscopy (XPS). As shown in Figure S14, the S 2p core-level XPS spectra of the channels present prominent emergence of S 2p peaks corresponding to TFSI⁻ anions from 172 eV to 168 eV.^[5]^ The S 2p peak of TFSI⁻ anions rises gradually with repeated potentiation pulses. Changes in the intensity of the S 2p peak (ΔS_TFSI_) were traced as a function of pulse number during a cycle of potentiation. ΔS_TFSI_ was determined by subtracting the S 2p peak intensity from that of the pristine sample, as presented in Figure S14. The results show that a greater amount of TFSI⁻ anions are trapped in the channels of STrs containing more amorphous phases, i.e., [RR]₆₃. This observation strongly supports the findings described in association with Figure 2d: [RR]₆₃ STr can provide higher C* values and dominant capacitive current responses than STrs with [RR]₁₀₀ and [RR]₇₈. Trapped TFSI⁻ anions can be effectively extracted from the channels by depression pulses, thus LTD behavior can be emulated in STrs with decreases of G_channel_.

*
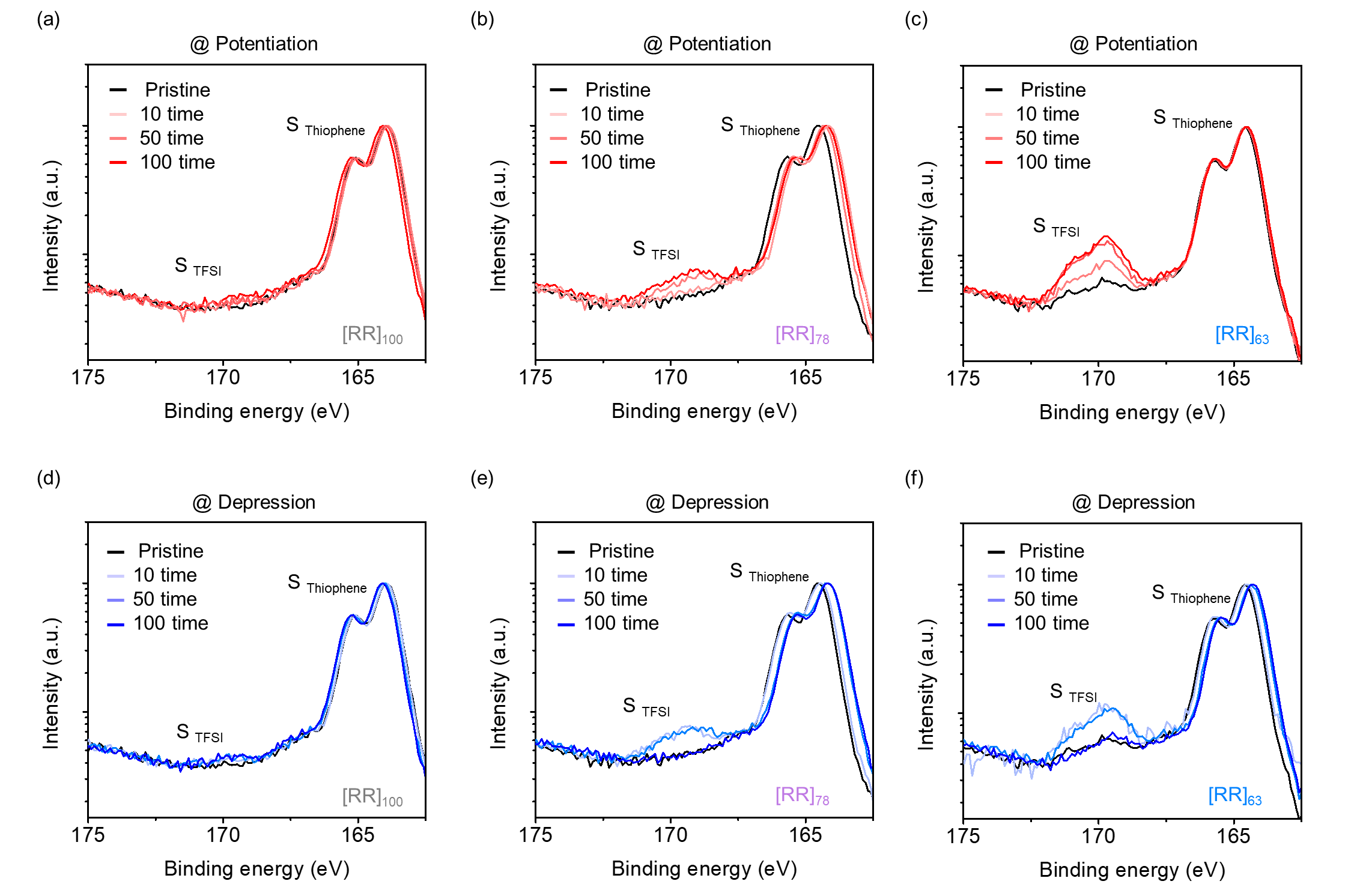
*

Figure S14. Analysis of the S 2p core-level peaks in RR-b-RRa P3HT STrs by XPS: (a-c) potentiation conditions and (d-f) depression conditions for [RR]_100_, [RR]_78_, and [RR]_63_, respectively.


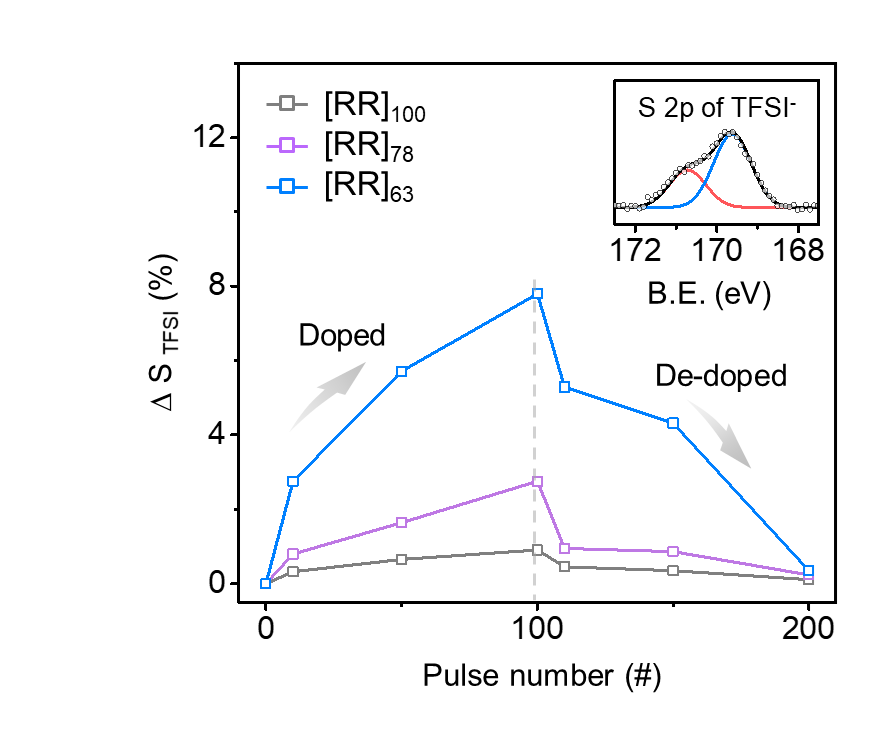


Figure S15. Summary of S 2p peak results representing TFSI⁻ anion variation in RR-b-RRa P3HT channels under gradual potentiation and depression. The inset shows the magnified binding energy (B.E.) region where S_TFSI_ appears. Potentiation pulses (-2.5 V, 100 ms) were applied to RR-b-RRa P3HT channels during doping behavior (pulse numbers 1 to 100), while depression pulses (+1.0 V, 100 ms) were sequentially applied (pulse numbers 101 to 200) for channel de-doping.

Note S6: Gaussian deconvolution of Raman spectra to identify the present position of charge states in RR-b-RRa P3HT films

Raman spectra of RR-b-RRa P3HT films were acquired at 633 nm as the excitation wavelength. 633 nm is known to have great resonance with the absorption of the conjugated polythiophene backbone.^[6]^ As shown in the Raman spectra in Figure S16, all films show typical resonant peaks corresponding to the polythiophene backbone, for example, peaks at 1448 and 1383 cm⁻¹ correspond to the intra-ring vibrational modes of *C_α_=C_β_* and *C_β_-C_β_*_._ ^[7]^ While no variations are observed in peak positions, the full width at half maximum of *C_α_=C_β_* differs among RR-b-RRa P3HT films, which is related to their ordering characteristics. A disordered chain vibrates at a higher frequency than an ordered chain due to its shorter conjugation length. As presented in Figure S17, oxidized chain conformations are created under potentiation progress. The scattering peak of *C_α_=C_β_* in all RR-b-RRa P3HT films exhibits a red shift (towards lower wavenumbers) with obvious growth of quinoid chain conformations. Since signals of various oxidized chain conformations from both crystalline and amorphous phases were combined in this peak, we separated them using six signature peaks by Gaussian deconvolution such as bipolarons (1495 and 1465 cm⁻¹), neutral polymer chains (1442 and 1455 cm⁻¹), and polarons (1425 and 1402 cm⁻¹) in the respective crystalline and amorphous phases, following the literature.^[7-8]^

*
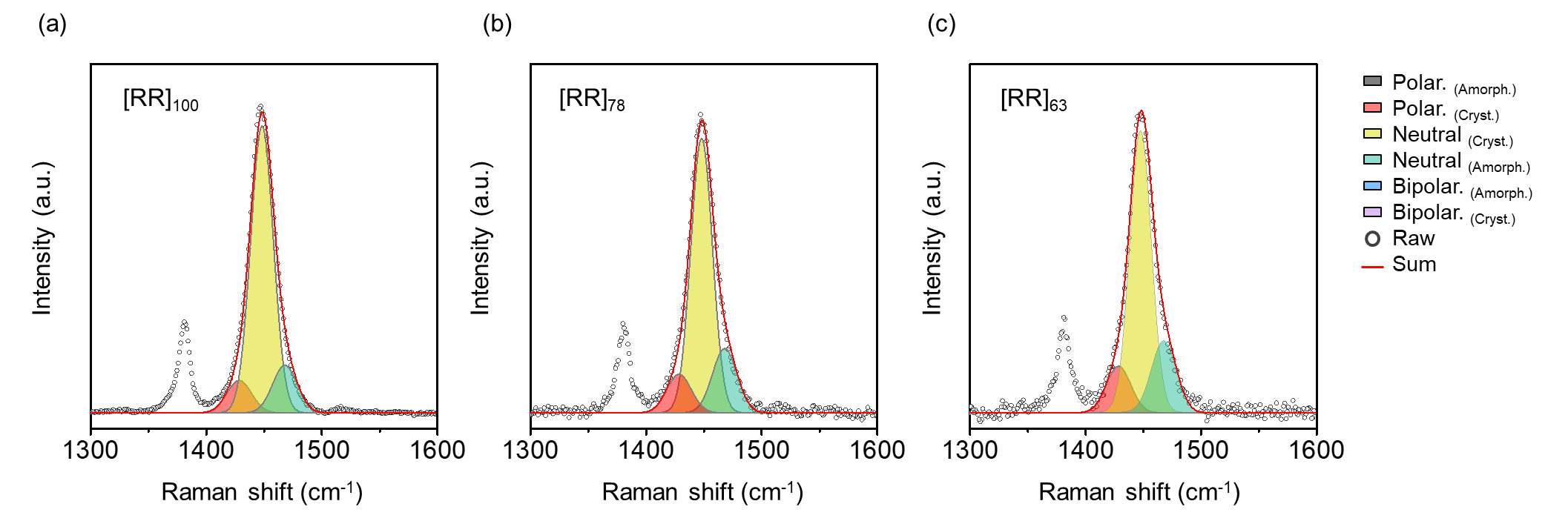
*

Figure S16. Raman spectra of pristine RR-b-RRa P3HT channels overlaid with their deconvolution results: (a) [RR]₁₀₀, (b) [RR]₇₈, and (c) [RR]₆₃.

*
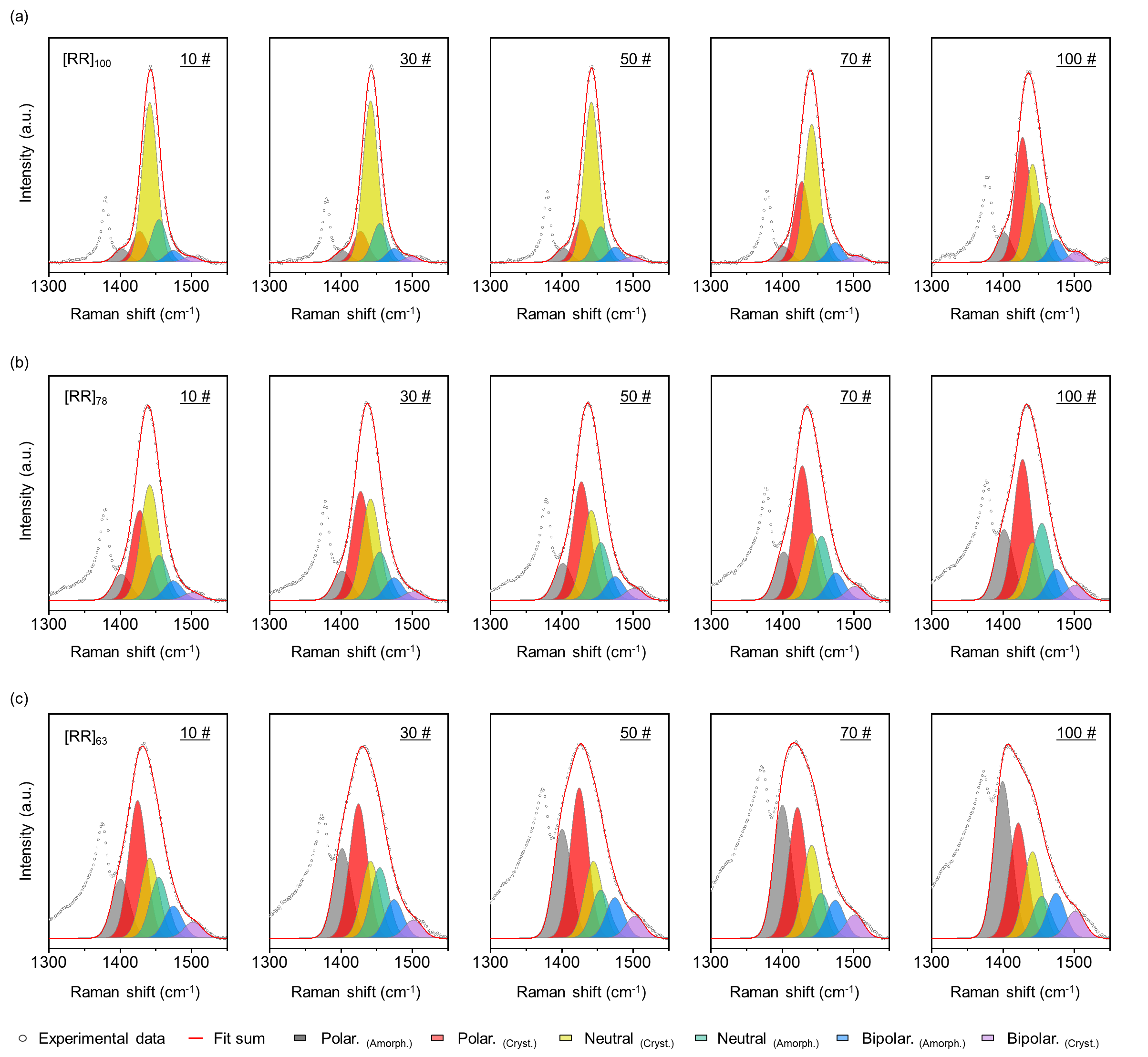
*Figure S17. Raman spectra of RR-b-RRa P3HT channels under sequential potentiation bias conditions, overlaid with their deconvolution results: (a) [RR]₁₀₀, (b) [RR]₇₈, and (c) [RR]₆₃.

Table S3. Deconvolution results of Raman peaks under sequential potentiation conditions

*
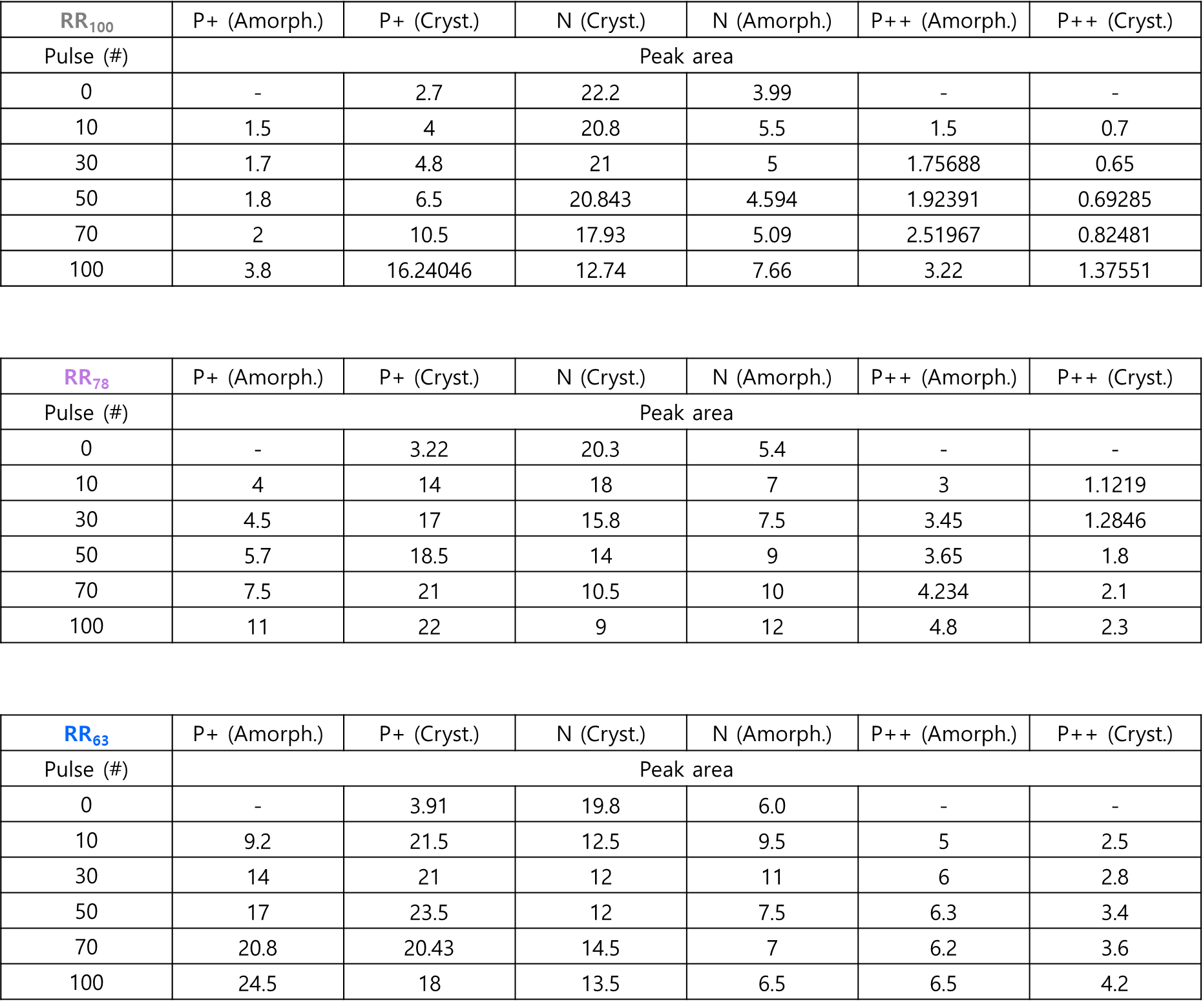
*

Note S7: Comparison of long-term retention of STrs with decay behaviors of charge states.

Long-term state retention behaviors between RR-b-RRa P3HT STrs were compared by analyzing the G_channel_ decay time constants (τ). Figure S18a shows the retention curves of STrs under various conductance states. For this experiment, [RR]₁₀₀-, [RR]₇₈-, and [RR]₆₃-STrs were programmed to match G_channel_ values of approximately 0.25, 0.6, and 1.1 mS, representing low, intermediate, and high doping states, respectively. The τ values were estimated based on the following equation.^[9]^

$G_{Channel, norm} =G_{Channel,norm}^{\mathrm{base}}+A_{1}exp(-t/\tau_{1})+A_{2}exp(-t/\tau_{2})+A_{3}exp(-t/\tau_{3})$

where *A*_i_ are the relative contributions of relaxation type, *t* is time, and $\tau_{i}$ are relaxation times (1 = depolarization of electron double layer (EDL), 2 = rapid back-diffusion of doped anions, and 3 = slow back diffusion of doped anions).

As shown in Figure S18b, τ₁, which corresponds to spontaneous ionic relaxation at the electrical double layer (EDL) near the channel/electrolyte interface, remains relatively constant (1.7-3.2) across all devices and doping levels. However, slower decay components (τ₂ and τ₃), which reflect the back-diffusion dynamics of doped TFSI⁻ anions, show significant differences depending on the channel rDoC. At low doping states, the [RR]₁₀₀-STr with high crystallinity exhibits τ₂ and τ₃ values that are 1.85 and 1.92 times higher, respectively, than those of [RR]₆₃-STr. This indicates slower ion back-diffusion in more crystalline films under weakly doped conditions. However, as the doping level increases, a distinct trend emerges: devices with higher amorphous content begin to exhibit superior retention behavior. At intermediate doping states, the τ₂ and τ₃ values of [RR]₆₃-STr increase markedly—becoming 2.6 and 1.8 times greater, respectively, than those observed under low doping. In contrast, the corresponding values for [RR]₁₀₀-STr decrease with increasing doping level, indicating reduced retention stability. These results reflect substantial suppression of ion back-diffusion in amorphous-rich channels at moderate doping levels. As the doping level further increases, the τ₂ and τ₃ values for all devices exhibit a slight decrease compared to those at the intermediate state, likely due to saturation of available trapping sites or increased structural relaxation. Nevertheless, [RR]₆₃-STr still maintains significantly larger τ₂ and τ₃ values than [RR]₁₀₀-STr, demonstrating that even under high doping conditions, the amorphous-rich channel continues to impede ion back-diffusion more effectively. This delayed relaxation, attributed to disordered lamellar structures and strong Coulombic interactions, results in the longest τ₃ observed among all devices at elevated conductance states. A similar trend is observed during long-term retention measurements over 1000 seconds. As shown in Figure S19, [RR]₆₃, which has the lowest channel rDoC, exhibits τ₂ and τ₃ values that are 25% and 82% higher, respectively, than those of [RR]₁₀₀. These results suggest that the enhanced retention behavior in devices with lower rDoC originates from pronounced retardation of the back-diffusion of trapped anions by strong Coulombic interactions.

Furthermore, we investigated the retention characteristics of doped charge carriers by monitoring the decay of optical absorption associated with polaron and bipolaron states (Figure S22). To ensure a fair comparison, the doping bias was individually optimized for each RR-b-RRa P3HT sample so that the initial ΔAbs at 800 nm and 1600 nm were matched across all samples. For polaronic states, the 800 nm absorption was monitored after removal of the doping bias. As shown in Figure S22a, the [RR]₆₃ film exhibited the slowest decay, followed by [RR]₇₈ and [RR]₁₀₀. Single-exponential fitting of the decay curves revealed τ values of 239.3 s for [RR]₆₃, 106.8 s for [RR]₇₈, and 57.9 s for [RR]₁₀₀, clearly demonstrating that higher amorphous content significantly retards the de-doping process (Figure S22b). In addition, bipolaron retention was also evaluated by analyzing the decay of 1600 nm absorption. As shown in Figures S22c and S22d, although the overall decay was faster than in the polaronic case—consistent with the less stable nature of bipolarons—the same trend persisted. The [RR]₆₃ film maintained the longest τ, again confirming the stabilizing effect of amorphous phases on doped states. This trend is attributed to stronger ionic trapping and structural disorder in amorphous-rich channels, which hinder the back-diffusion of TFSI⁻ anions.


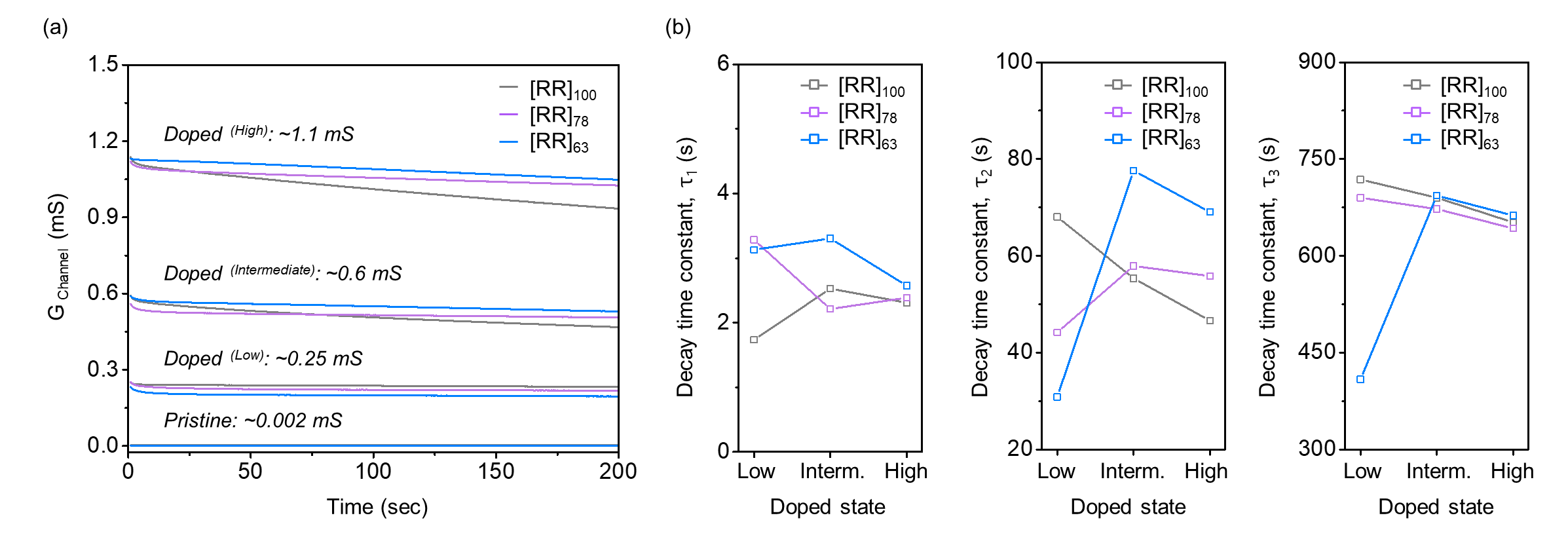


Figure S18. Comparison of retention behaviors of RR-b-RRa P3HT STrs, where all devices were programmed to identical conductance states. (a) State retention curves of RR-b-RRa P3HT STrs programmed to three target conductance values: 0.25, 0.6, and 1.1 mS. (b) Extracted decay time constants (τ₁, τ₂, and τ₃) of each device as a function of programmed conductance state.


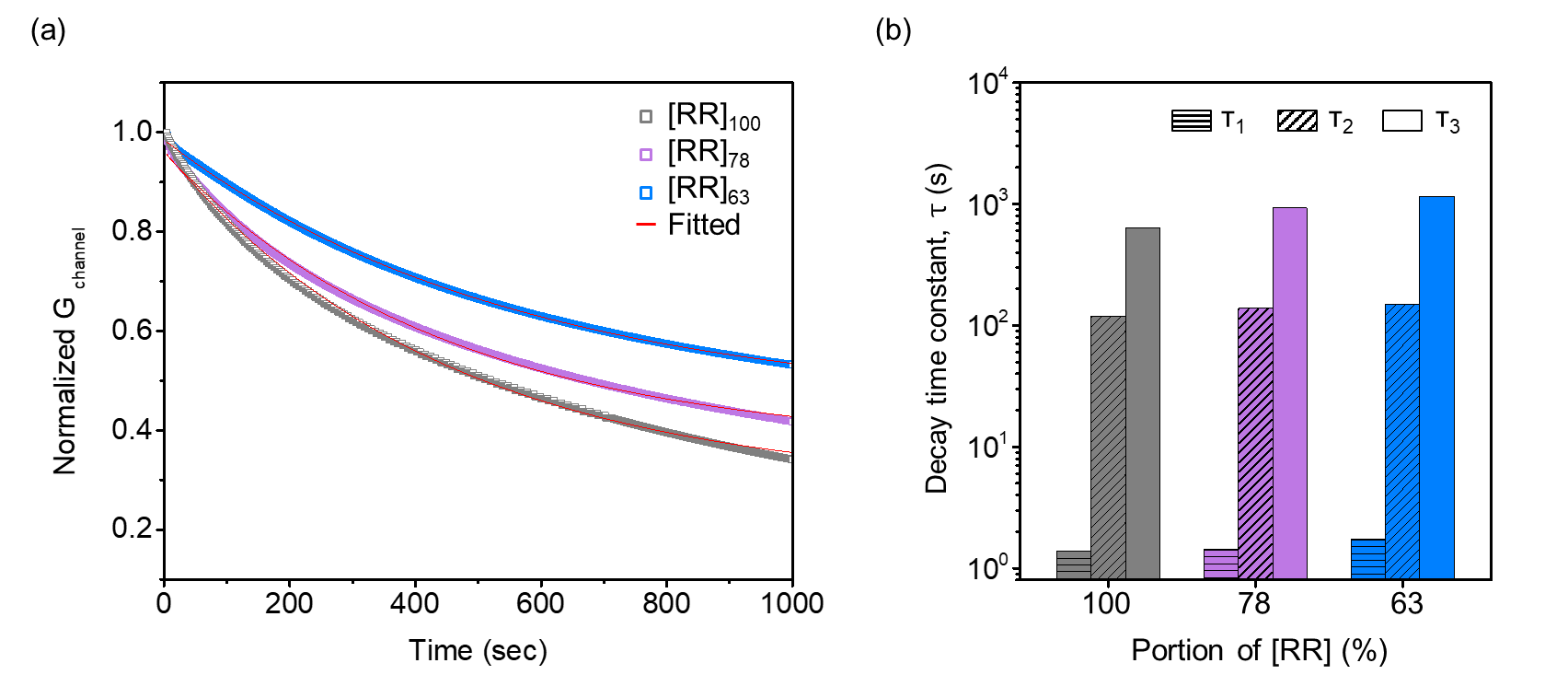


Figure S19. (a) Fitted curves of normalized retention at highly doped STr states. Retention behaviors of all devices were observed after potentiation with 100 pulses (-2.5 V, 100 ms). (b) Extracted decay time constants τ for RR-b-RRa P3HT STrs.


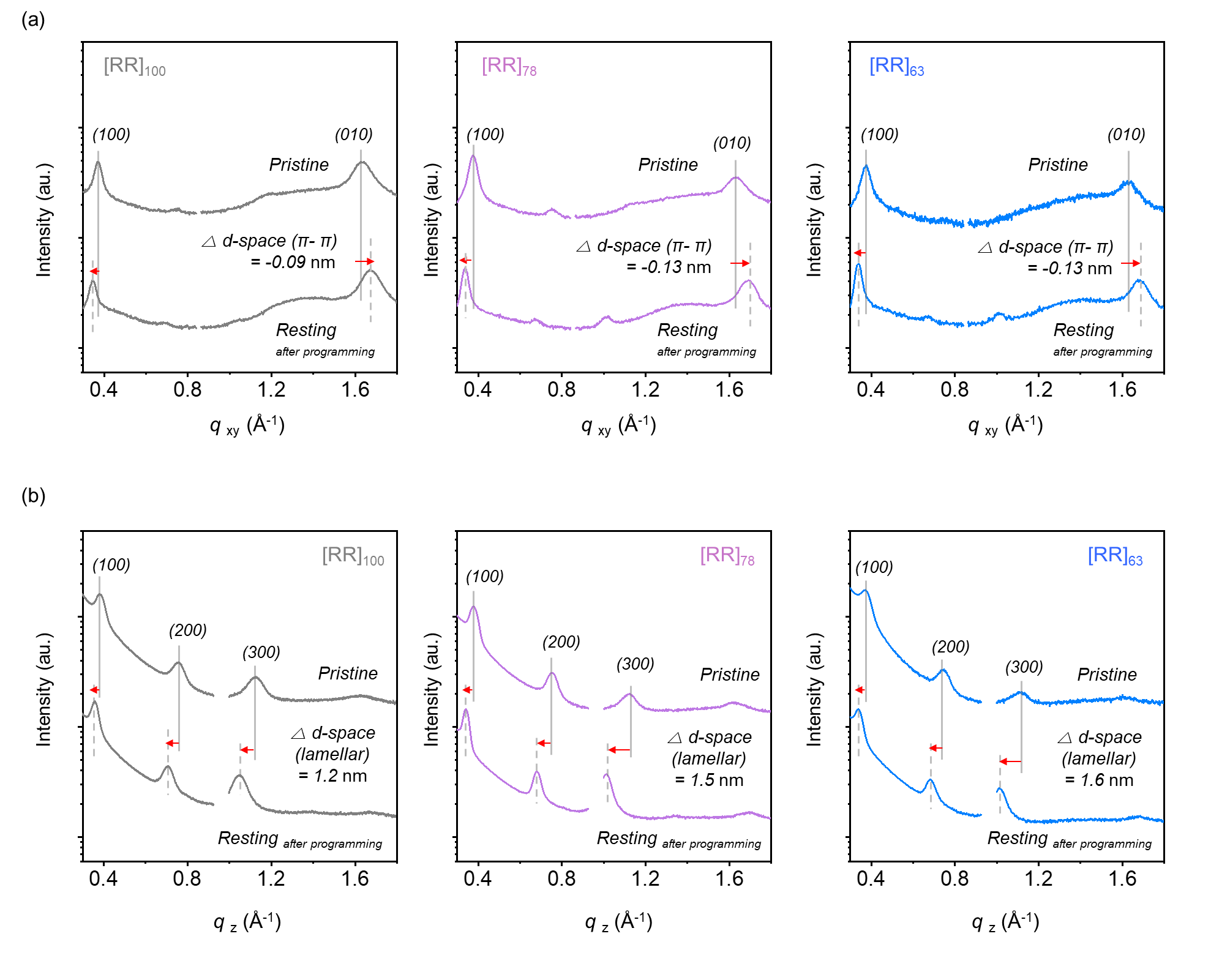


Figure S20. Comparison of the 1D line-cut profiles from GIWAXS results in the (a) in-plane and (b) out-of-plane directions. The variation in lamellar and π-π stacking distances due to electrochemical doping was calculated using the (200) and (010) diffraction peaks, respectively.


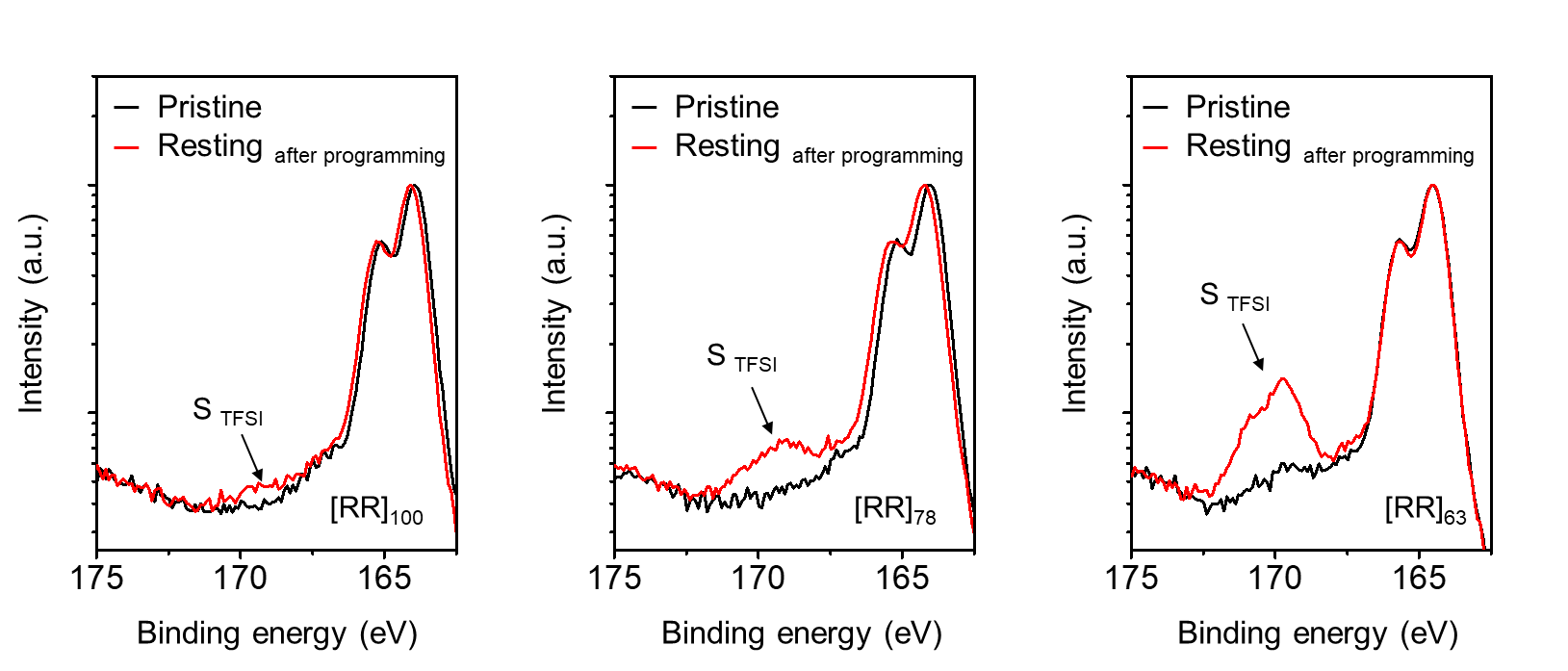


Figure S21. Comparison of S 2p core-level spectra of RR-b-RRa P3HT films. The black line represents the pristine state, while the red line corresponds to the doped state. The doped-state sample was prepared by applying 100 programming pulses (-2.5 V, 100 ms), followed by a rest period of over 15 minutes under open-circuit conditions before measurement.


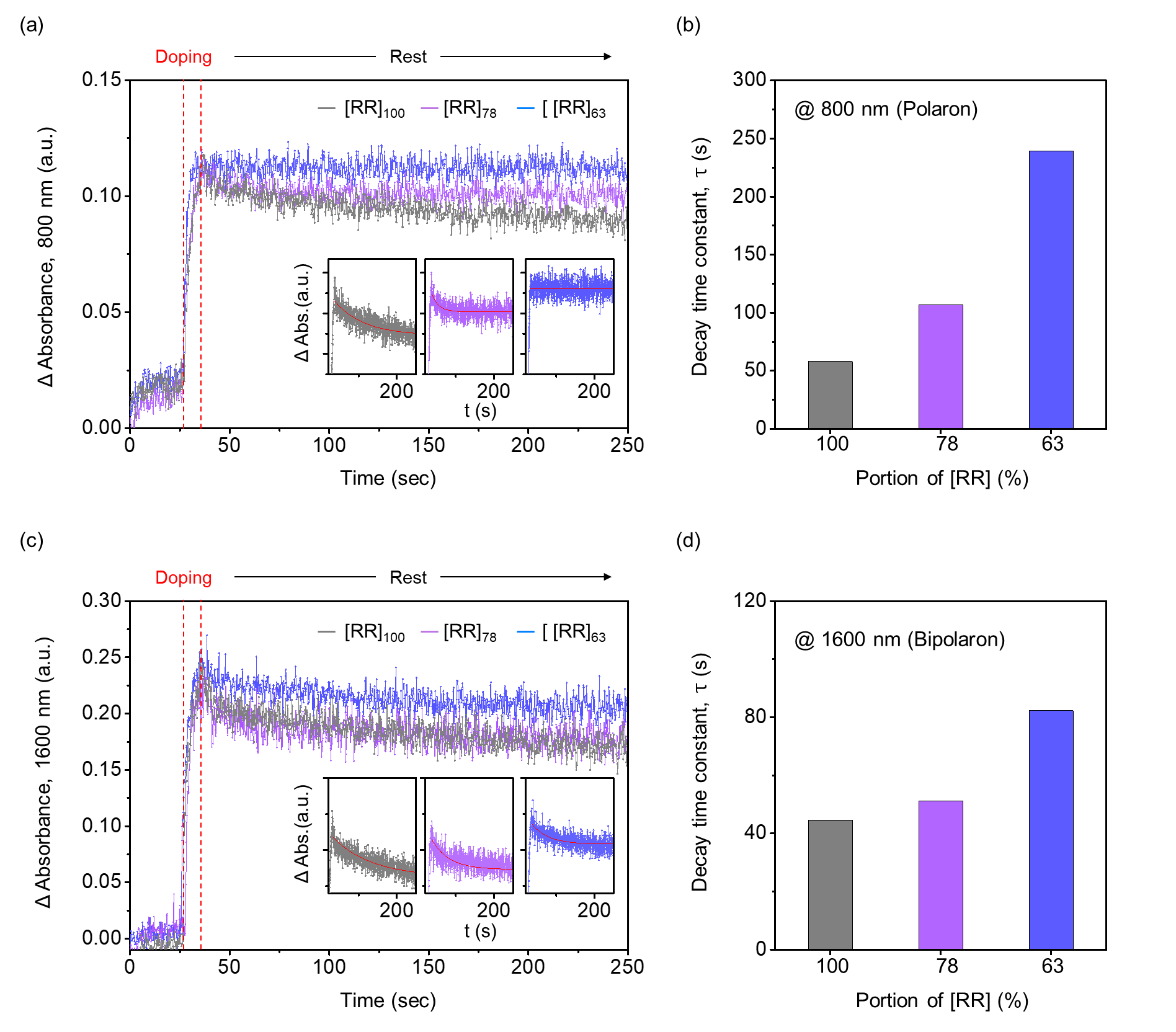


Figure S22. Time-resolved decay behaviors of electrochemically generated charge states in RR-b-RRa P3HT films. (a) Time-resolved decay of polaron absorption (800 nm) after doping. To match initial ΔAbs₈₀₀_nm_ across films, doping bias amplitudes were adjusted to -1.5 V ([RR]₁₀₀), -2.0 V ([RR]₇₈), and -2.5 V ([RR]₆₃), respectively. (b) Extracted τ values of RR-b-RRa P3HT films from respective polaron decay curves. (c) Time-resolved decay of bipolaron absorption (1600 nm) after doping. Initial ΔAbs₁₆₀₀_nm_ was fixed at 0.23 by adjusting doping bias amplitudes to -2.4 V ([RR]₁₀₀), -2.2 V ([RR]₇₈), and -2.1 V ([RR]₆₃), respectively. (d) Extracted τ values of RR-b-RRa P3HT films from respective bipolaron decay curves.


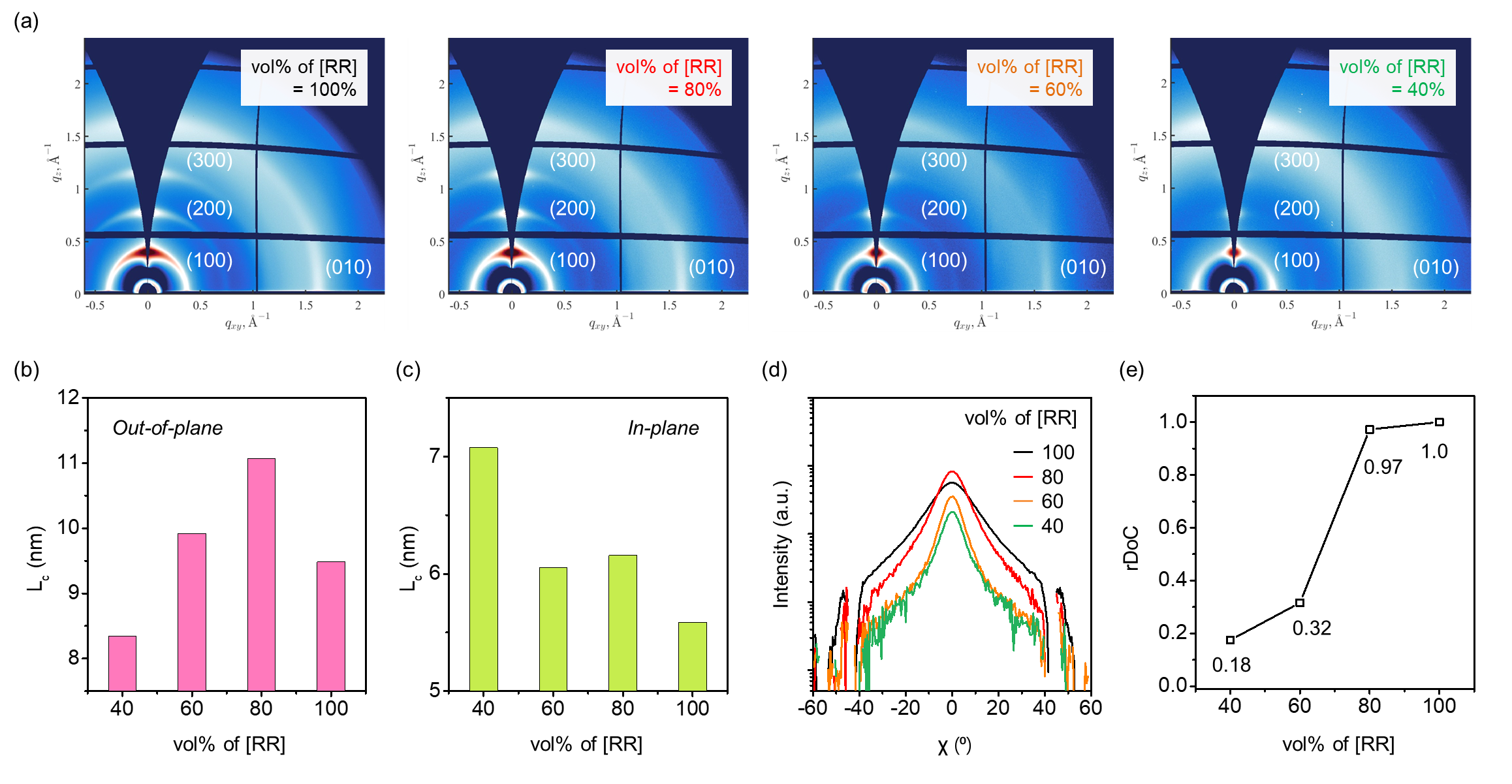


Figure S23. Microstructural characterization of RR:RRa P3HT blend films: (a) 2D-GIWAXS images of blend films, (b,c) L_c_ of crystalline domains as a function of [RR] fraction, (d) pole figures of (200) scattering peaks in the angular range of ±60°, and (e) rDoC for RR:RRa P3HT blend films.


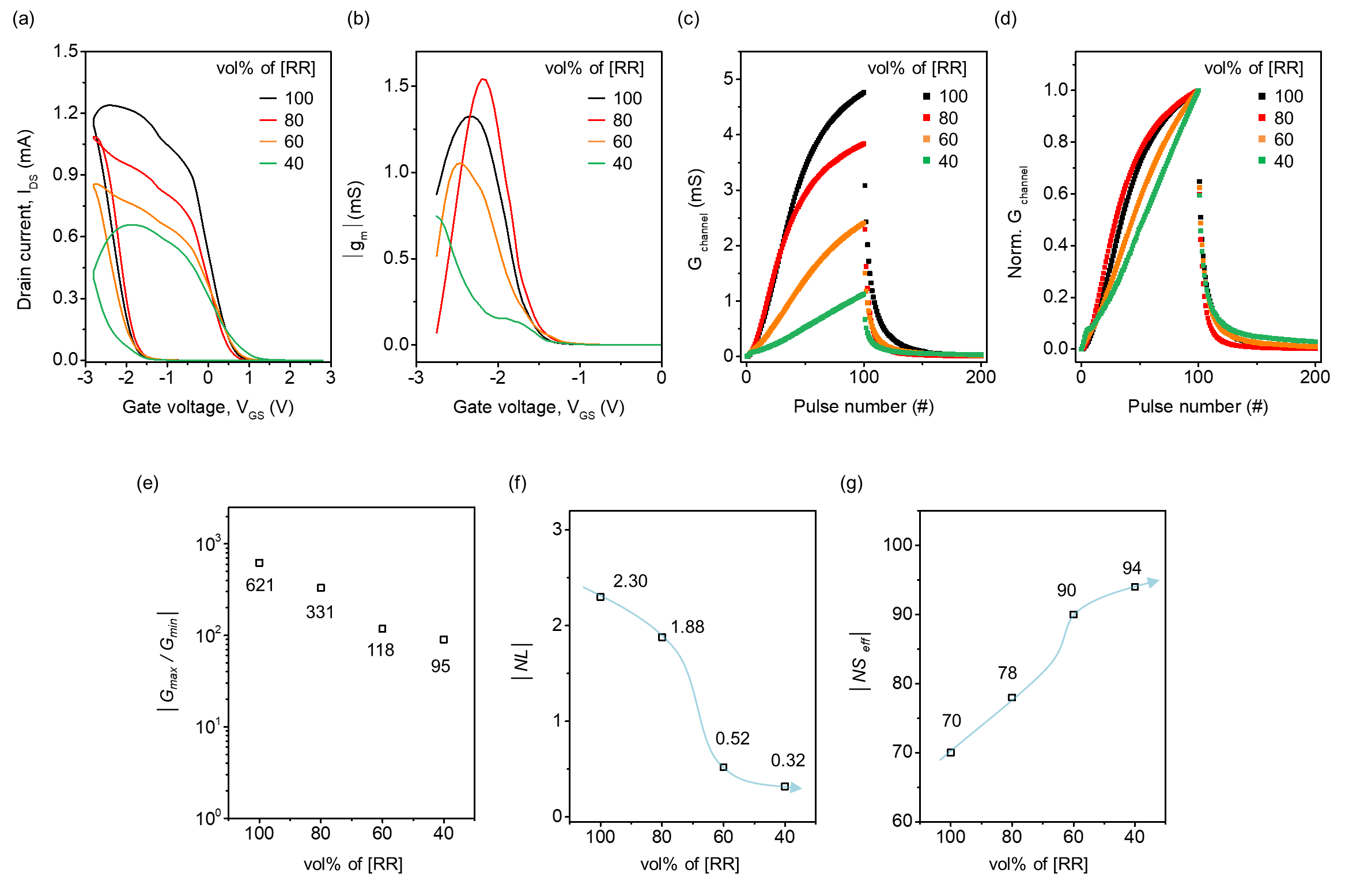
 Figure S24. Synaptic behaviors of RR:RRa P3HT blend-based STrs: (a) Transfer curves and (b) calculated gm values. (c) Typical LTP/LTD curves. (d) Normalized LTP/LTD curves. (e) Calculated dynamic range, (f) NL values, and (g) NS_eff_ values.


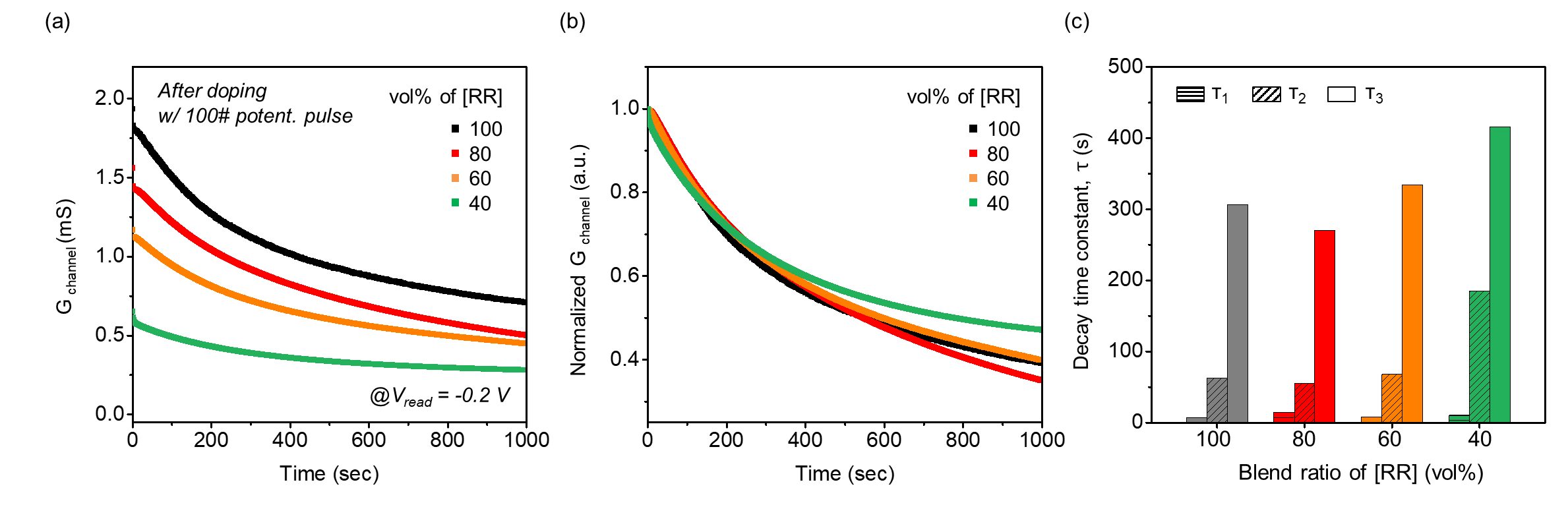


Figure S25. Retention behavior of RR:RRa blend P3HT STrs: (a) G_channel_ retention over 10³ seconds, (b) normalized G_channel_ as a function of elapsed time, and (c) decay time constant τ.


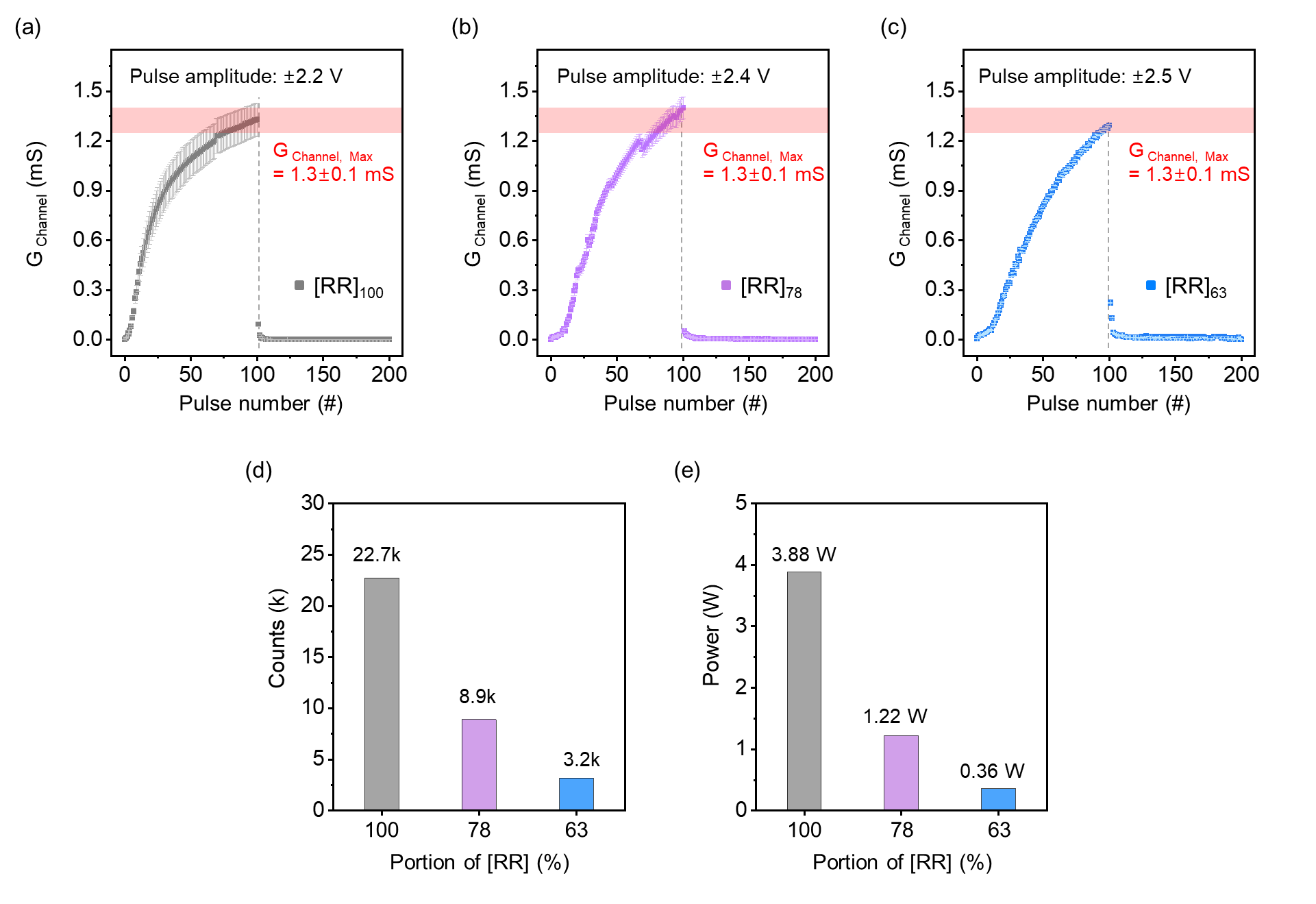


Figure S26. Validation of the performance of RR-b-RRa P3HT STrs and neural network under a fixed conductance range. (a–c) The averaged G_c_ₕₐₙₙₑₗ values for repetitive 10-cycle LTP/LTD curves of [RR]_100_-, [RR]_78_-, and [RR]_63_-STrs. Each device was programmed and erased using different pulse amplitudes to set a fixed maximum conductance level. (d) Simulated program/erase (P/E) counts and (e) power consumption during the HAR recognition task.


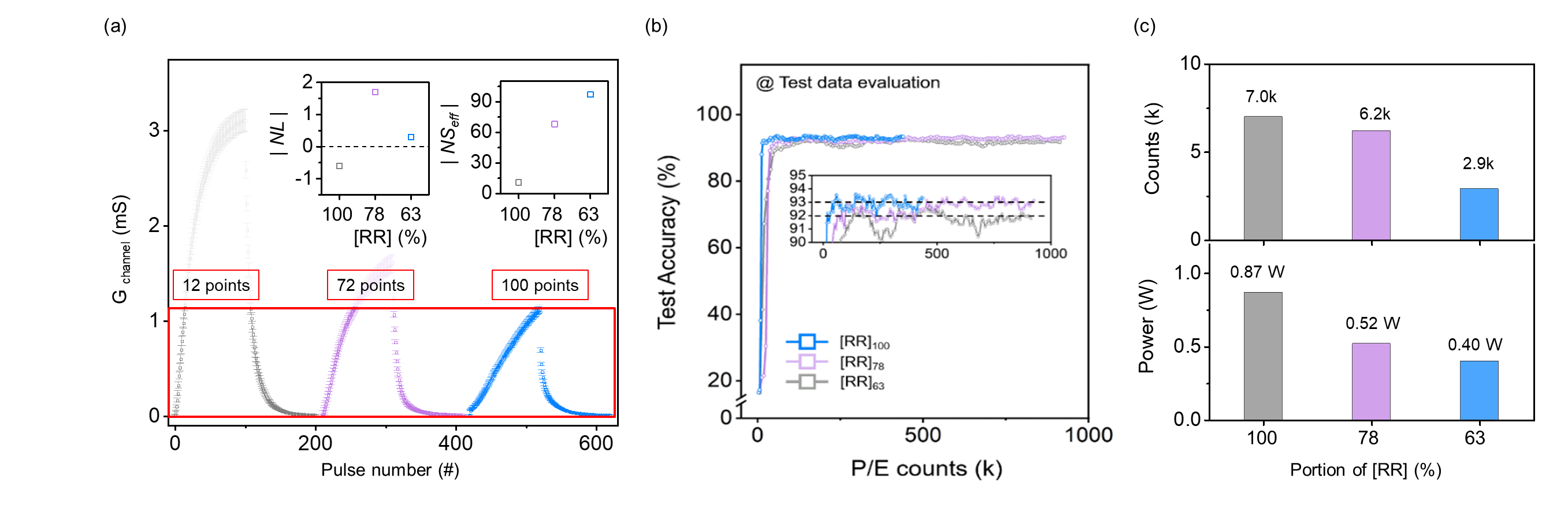


Figure S27. Deep neural network (DNN) simulation under limited conductance range conditions. (a) The utilized conductance ranges of each RR-b-RRa P3HT STr, extracted from their respective LTP curves, are highlighted with red boxes. The number of conductance states used for the simulation were as follows: [RR]_100_ (12 points), [RR]_78_ (72 points), and [RR]_63_ (100 points). The inset plots were calculated NL and NS_eff_ values, extracted from potentiation curves in limited conductance range. (b) Test accuracy evaluation performed with the conductance range limited to approximately 1.1 mS. (c) Reduction in program/erase (P/E) cycles and decrease in power consumption observed as the [RR] content decreases.


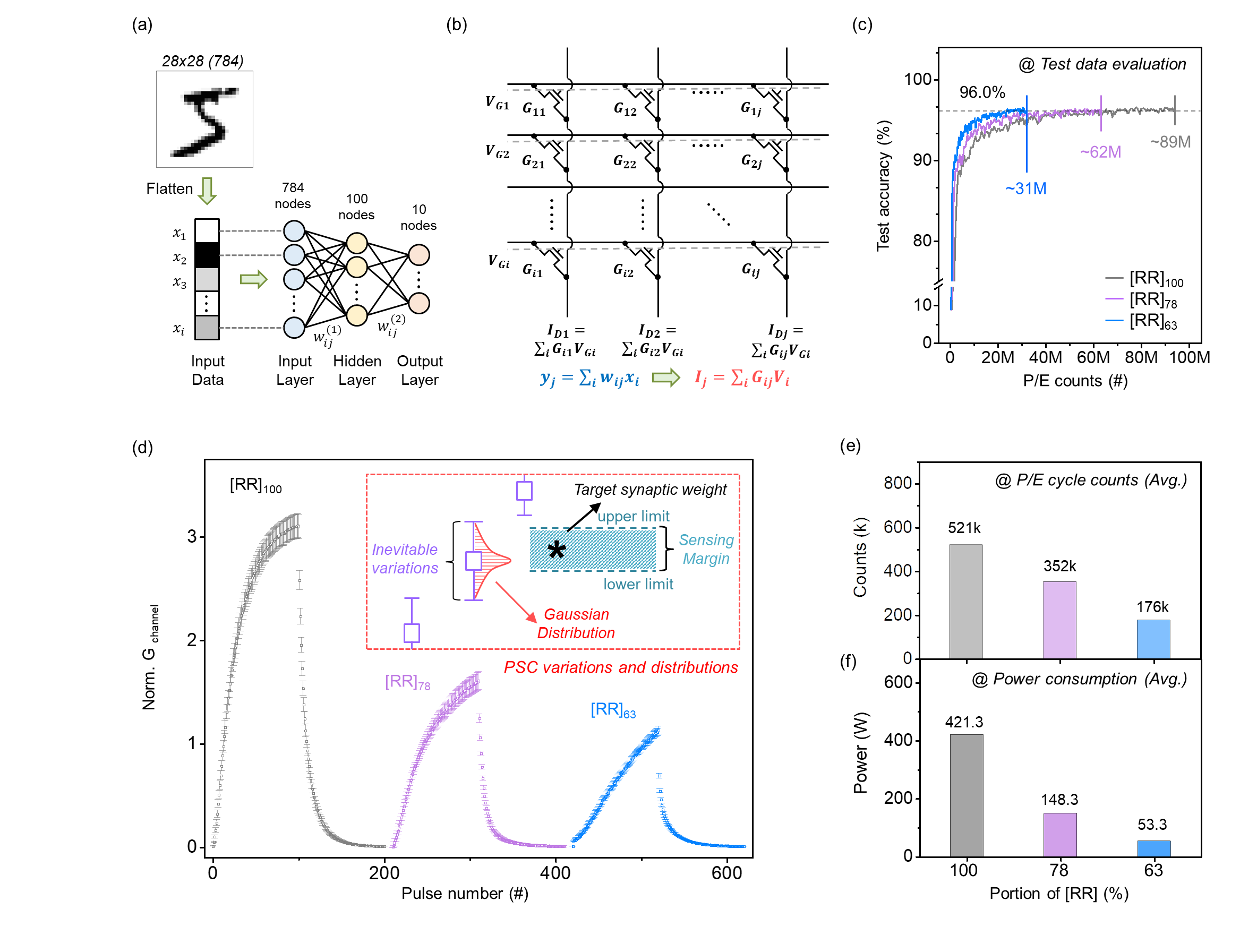


Figure S28. RR-b-RRa P3HT STrs for Deep Neural Network (DNN) applications. (a) Configuration of a MLP for MNIST classification using RR-b-RRa P3HT for synaptic weights. (b) Crossbar-array structure for vector-matrix multiplication. (c) Classification accuracy results demonstrating the effectiveness of RR-b-RRa P3HT STrs. (d) Schematic of modeled target PSC values with sensing margins, accounting for characteristic variation in RR-b-RRa P3HT STrs. (e) Reduction in program/erase cycles and (f) decreased power consumption, with decreasing [RR] portion.

**Supplementary references**

[1] J.-S. Kim, J.-E. Choi, H. Park, Y. Kim, H. J. Kim, J. Han, J. M. Shin, B. J. Kim, *Polymer Chemistry* **2019**, 10, 3030.

[2] Y. Choi, S. Oh, C. Qian, J. H. Park, J. H. Cho, *Nat Commun* **2020**, 11, 4595.

[3] G. Strobl, *The Physics of Polymers; concepts for understanding their structures and behavior*, SPRINGER NATURE, **2007**.

[4] P. Kohn, S. Huettner, H. Komber, V. Senkovskyy, R. Tkachov, A. Kiriy, R. H. Friend, U. Steiner, W. T. Huck, J. U. Sommer, M. Sommer, *J Am Chem Soc* **2012**, 134, 4790.

[5] V. Shutthanandan, M. Nandasiri, J. Zheng, M. H. Engelhard, W. Xu, S. Thevuthasan, V. Murugesan, *J Electron Spectros Relat Phenomena* **2019**, 231, 2.

[6] W. C. Tsoi, D. T. James, J. S. Kim, P. G. Nicholson, C. E. Murphy, D. D. Bradley, J. Nelson, J. S. Kim, *J Am Chem Soc* **2011**, 133, 9834.

[7] J. Nightingale, J. Wade, D. Moia, J. Nelson, J.-S. Kim, *J Phys Chem C* **2018**, 122, 29129.

[8] P. Cavassin, I. Holzer, D. Tsokkou, O. Bardagot, J. Rehault, N. Banerji, *Adv Mater* **2023**, 35, e2300308.

[9] G.-T. Go, Y. Lee, D.-G. Seo, M. Pei, W. Lee, H. Yang, T.-W. Lee, *Adv Intell Syst* **2020**, 2, 2000012.
